# HNF4α controls growth, identity, and KRAS inhibitor response in invasive mucinous adenocarcinoma of the lung

**DOI:** 10.1172/JCI198282

**Published:** 2026-05-12

**Authors:** Headtlove Essel Dadzie, Yangsook Song Green, Soledad A. Camolotto, Henry U. Arnold, Matthew Gumbleton, Minzhe Guo, Mari Mino-Kenudson, Yutaka Maeda, Benjamin T. Spike, Eric L. Snyder

**Affiliations:** 1 Huntsman Cancer Institute, and; 2 Department of Oncological Sciences, University of Utah, Salt Lake City, Utah, USA.; 3 Department of Internal Medicine, University of Utah, Salt Lake City, Utah, USA.; 4 Perinatal Institute, Division of Neonatology, Perinatal and Pulmonary Biology, Cincinnati Children’s Hospital Medical Center and the University of Cincinnati College of Medicine, Cincinnati, Ohio, USA.; 5 Department of Pathology, Massachusetts General Hospital and Harvard Medical School, Boston, Massachusetts, USA.; 6 Department of Pathology, University of Utah, Salt Lake City, Utah, USA.

**Keywords:** Oncology, Pulmonology, Lung cancer

## Abstract

Cellular plasticity is a hallmark of cancer, enabling tumor cells to alter identity and evade therapeutic pressure. In invasive mucinous adenocarcinoma of the lung (IMA), NK2 homeobox 1 (NKX2-1) loss triggers a pulmonary to gastric switch marked by aberrant activation of hepatocyte nuclear factor 4 alpha (HNF4α), a master regulator of gastrointestinal/hepatic differentiation. We show that HNF4α promoted IMA growth and activated a gastric pit cell–like program. Loss of HNF4α enabled forkhead box A1 and A2 (FoxA1/2) transcription factors to bind de novo sites and activate alternative, nongastric identities in IMA. HNF4α also established a mucinous program associated with tolerance to KRAS blockade, and loss of HNF4α enhanced response to KRAS^G12D^ inhibition. Mechanistically, HNF4α blocked cell-cycle exit in drug-tolerant persister cells and promoted activity of the antioxidant transcription factor nuclear factor erythroid 2–related factor 2 (NRF2). NRF2 activation partially rescued the effects of *Hnf4a* deletion on KRAS^G12D^ inhibition, whereas NRF2 inhibition enhanced sensitivity to KRAS^G12D^ blockade. Thus, HNF4α is a key regulator of growth, identity, and primary response to KRAS^G12D^ inhibition in IMA.

## Abstract 2

HNF4 plays a key role in the biology of invasive mucinous adenocarcinoma, a unique subtype of lung adenocarcinoma.

## Graphical abstract

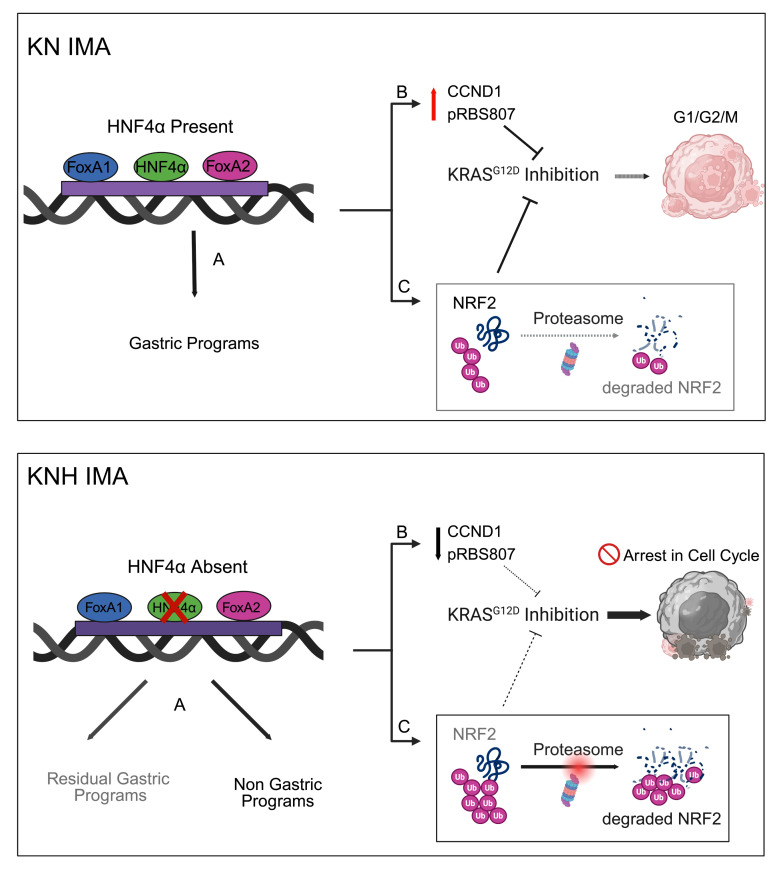

## Introduction

Cancer remains a leading cause of mortality worldwide, driven by its inherent molecular and cellular heterogeneity. Despite significant advances in molecular profiling and targeted therapies, treatment outcomes are still suboptimal across multiple cancer types (1). A key challenge in oncology is the remarkable plasticity of cancer cells, which enables dynamic shifts in cellular identity in response to genetic, epigenetic, and transcriptional cues (2, 3). This plasticity limits the durability of therapeutic responses and promotes tumor progression, metastasis, and recurrence (4).

Lung adenocarcinoma (LUAD), the most prevalent subtype of lung cancer (1), exemplifies these challenges due to its molecular heterogeneity and lineage plasticity (5). Although therapies targeting driver oncogenes such as EGFR (6), KRAS (particularly G12C) (7), and ALK (8) have transformed clinical care, resistance remains common (9). LUAD cells can transition from AT2-like states to alternative identities, including neuroendocrine, squamous, or gastric-like states, particularly under therapeutic pressure (10, 11). These transitions, driven by epigenetic and transcriptional reprogramming, increase intratumoral heterogeneity and pose barriers to effective treatment (4).

Invasive mucinous adenocarcinoma (IMA) of the lung is a distinct LUAD subtype (~5%–10% of cases) (12) that provides a compelling model to study transcription factor–driven lineage plasticity. Most IMA cases arise through loss of NKX2-1, a master regulator of pulmonary epithelial identity, and acquisition of gastric identity via forkhead box A1 and A2–mediated (FoxA1/2-mediated) transcriptional reprogramming (11, 13, 14).

The adult gastric epithelium consists of multiple specialized cell types (e.g., pit, neck, chief, parietal, tuft, and enteroendocrine) derived from isthmus progenitors at steady state (15, 16). IMA tumor cells partially recapitulate gastric differentiation, including a major pit-like population with abundant mucin production and a minor tuft-like cell population (17). Tuft cells are rare chemosensory sentinels with emerging roles in cancer (18), and tuft-like transcriptional states have been described in other epithelial malignancies (19–21). We have previously shown that levels of MEK/ERK and WNT signaling dictate the specific gastric cell types that IMA cells resemble (17), consistent with their roles in normal gastric differentiation (22).

The nuclear receptor HNF4α (23) is highly expressed in human IMA and is required for expression of the gastric pit cell marker gastrokine 1 (GKN1) in a genetically engineered mouse model (GEMM) of IMA (14, 24). Although best characterized in the liver, HNF4α also regulates epithelial cell differentiation and homeostasis in the stomach and intestine and controls pancreatic β cell function (25–27). In the lung, HNF4α is normally absent but becomes aberrantly induced in IMA through the activity of the pioneer factors FoxA1/2, which are broadly required to establish gastrointestinal (GI) chromatin landscapes (14, 28). HNF4α has context-dependent tumor-suppressive and oncogenic roles across distinct cancer subtypes (29, 30).

Up to 75% of IMAs harbor *KRAS* mutations (31), with the G12D variant being the most common (32). KRAS^G12D^-targeted therapies are in preclinical and clinical evaluation for multiple cancers (33–35). However, the mechanisms of primary response and acquired resistance to KRAS inhibition remain poorly defined (36). Emerging evidence implicates the antioxidant transcription factor NRF2 as a modulator of response to KRAS inhibitor in LUAD (37). Under basal conditions, KEAP1 targets NRF2 for degradation, whereas oxidative stress or *KEAP1* mutation stabilizes NRF2 (38). High NRF2 activity is linked to KRAS inhibitor resistance in WT *KEAP1* LUAD, suggesting that genetic and nongenetic mechanisms can activate NRF2 and blunt the therapeutic response (37, 39, 40). Recent work in renal epithelial cells showed a functional physical interaction between NRF2 and HNF4α, raising the possibility that HNF4α might contribute to NRF2 activation and therapeutic resistance in IMA (41).

Using a GEMM that recapitulates key features of human IMA, we previously showed that *Hnf4a* deletion partially impairs IMA initiation (14). Complementary studies in human cell lines have proposed mechanisms by which HNF4α supports IMA growth (42, 43), and recent work demonstrated enrichment of HNF4α and other GI lineage markers in drug-tolerant persister (DTP) cells arising in LUAD GEMMs treated with RAS inhibitors (44). Despite these observations, key mechanistic questions remain. Is HNF4α required for the maintenance of established IMA growth in vivo? Does HNF4α cooperate with lineage specifiers such as FoxA1/2 to regulate IMA cell identity? And does HNF4α directly modulate responses to KRAS inhibition, or is its expression simply associated with the DTP state?

Here, we addressed these questions using complementary IMA GEMMs, mouse-derived organoids (MDOs) and patient-derived organoids (PDOs) and integrative multiomics approaches (45) to define how HNF4α regulates IMA lineage fidelity and response to targeted therapy. Together, our findings establish HNF4α as a key regulator of IMA identity downstream of FoxA1/2 and implicate disruption of the HNF4α/NRF2 axis as a potential strategy to enhance primary responses to KRAS inhibition.

## Results

### HNF4α promotes in vivo growth of established IMA.

**Figure 1:**
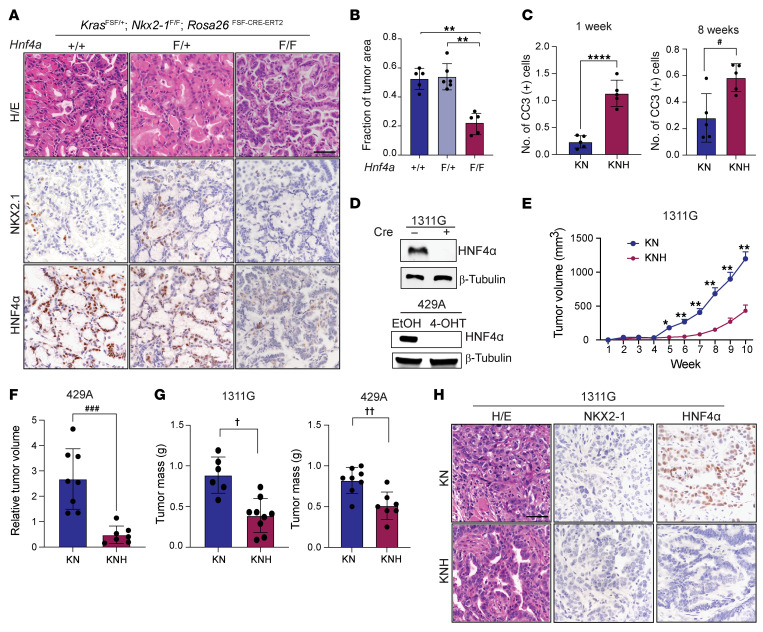
HNF4α is essential for in vivo growth of established IMA. (**A**) Representative H&E and IHC images for NKX2-1 and HNF4α in IMA GEMMs expressing *Hnf4a*^+/+^, *Hnf4a*^fl/+^, and *Hnf4a^fl/fl^*. Scale bar: 100 μm. (**B**) Tumor burden in *Hnf4a*^+/+^ (*n* = 5), *Hnf4a*^fl/+^ (*n* = 6), and *Hnf4a^fl/fl^* (*n* = 5) GEMMs 14 weeks PTI with Ad5mSPC-FlpO (1 × 10^8^ PFU/mouse). **P < 0.01, by Mann-Whitney *U* test. (**C**) Quantification of cleaved caspase 3 (CC3) in KN and KNH GEMMs 1 week (left) or 8 weeks (right) after first i.p. dose of tamoxifen. ^#^*P* = 0.0123; ****P < 0.0001, by unpaired, 2-tailed Student’s *t* test. (**D**) Representative immunoblots for the indicated proteins in KN and KNH of 1311G and 429A organoids. (**E**) Longitudinal s.c. tumor volume of 1311G organoids in NSG mice. *P < 0.05 and **P < 0.01, by unpaired, 2-tailed Student’s *t* test. (**F**) Fold change in 429A s.c. tumor volume in NRG mice after 10 days of tamoxifen. ^###^*P* = 0.0004, by unpaired, 2-tailed Student’s *t* test. (**G**) Endpoint tumor mass of KN and KNH tumors from NSG or NRG mice transplanted with 1311G (left, ^†^*P* = 0.0013) or 429A (right, ^††^*P* = 0.0030) organoids; unpaired, 2-tailed Student’s *t* test. (**H**) Representative H&E and IHC images of NKX2-1 and HNF4α staining in s.c. tumors generated from isogenic 1311G KN and KNH organoids in NSG mice. Scale bar: 100 μm.

To investigate the role of HNF4α in established IMA tumors in vivo, we developed an advanced GEMM utilizing sequential activation of Flp and Cre recombinases for the conditional deletion of *Hnf4a* in established tumors (Supplemental Figure 1A; supplemental material available online with this article; https://doi.org/10.1172/JCI198282DS1). Tumors were initiated by intratracheal delivery of murine surfactant protein C (SPC) promoter–driven Flpo adenovirus (11), activating *Kras^G12D^* and *Cre^ERT2^* in distal lung epithelial cells (46, 47). Tamoxifen induced efficient Cre^ERT2^-mediated recombination, resulting in near-complete deletion of *Nkx2-1*, in tumors harboring WT *Hnf4a* alleles (*Kras^FS-G12D/+^* *Rosa26^FSF-CreERT2^ Nkx2-1^fl/fl^ Hnf4a^+/+^* or *Kras^FSF-G12D/+^* *Rosa26^FSF-CreERT2^ Nkx2-1^fl/fl^ Hnf4a^fl/+^*, hereafter referred to as KN) and in those with homozygous deletion of floxed *Hnf4a* alleles (*Kras^FSF-G12D/+^* *Rosa26^FSF-CreERT2^ Nkx2-1^fl/fl^ Hnf4a^fl/fl^*, designated as KNH*)* (Figure 1A).

Morphologically, KNH GEMM tumors exhibited an increased nucleus-to-cytoplasm ratio and reduced cytoplasmic volume compared with KN tumors (Figure 1A). At 14 weeks post tumor initiation (PTI), KNH mice had approximately 50% lower tumor burden compared with KN controls (*P* < 0.01; Figure 1B). Because there were no differences in morphology or tumor burden between *Hnf4a^+/+^* and *Hnf4a^fl/+^* KN tumors, we used *Hnf4a^fl/+^* KN mice as controls in subsequent experiments. *Hnf4a* deletion increased apoptosis as early as 1 week after the first tamoxifen dose and persisted for 8 weeks (Figure 1C), without significantly affecting proliferation rates at either time point (Supplemental Figure 1, B–E).

To uncouple *Hnf4a* deletion from *Nkx2-1* deletion, we derived 2 isogenic organoid lines from KNH mice (1311G and 429A) that were NKX2-1 deficient and HNF4α proficient. These cell lines were subcloned and screened for stochastic retention of HNF4α expression (48, 49) (Supplemental Figure 1F and Supplemental Methods). We then deleted *Hnf4a* in 1311G and 429A cell lines using Ad5CMVCre and 4-hydroxy-tamoxifen (4-OHT), respectively (Figure 1D). *Hnf4a* deletion did not affect the proliferation of either organoid line in vitro (Supplemental Figure 1, G–J), but it significantly impaired the growth of both organoid lines in vivo (Figure 1, E–G). Histological analysis confirmed that tumors derived from both organoid lines retained their expected morphology and protein expression (Figure 1H and Supplemental Figure 1K). These findings show that HNF4α promoted IMA growth and suppressed apoptosis in vivo.

### HNF4α directly activates a gastric differentiation program in IMA.

To understand how HNF4α governs identity in IMA, we systematically mapped its direct targets and downstream transcriptional programs across multiple models. To enable high-purity isolation of tumor nuclei for chromatin profiling, we incorporated a *Sun1*-*sfGFP* Cre reporter allele (50, 51) into KN and KNH IMA GEMMs, allowing efficient isolation of GFP^+^ nuclei or live cells by FACS (28).

**Figure 2:**
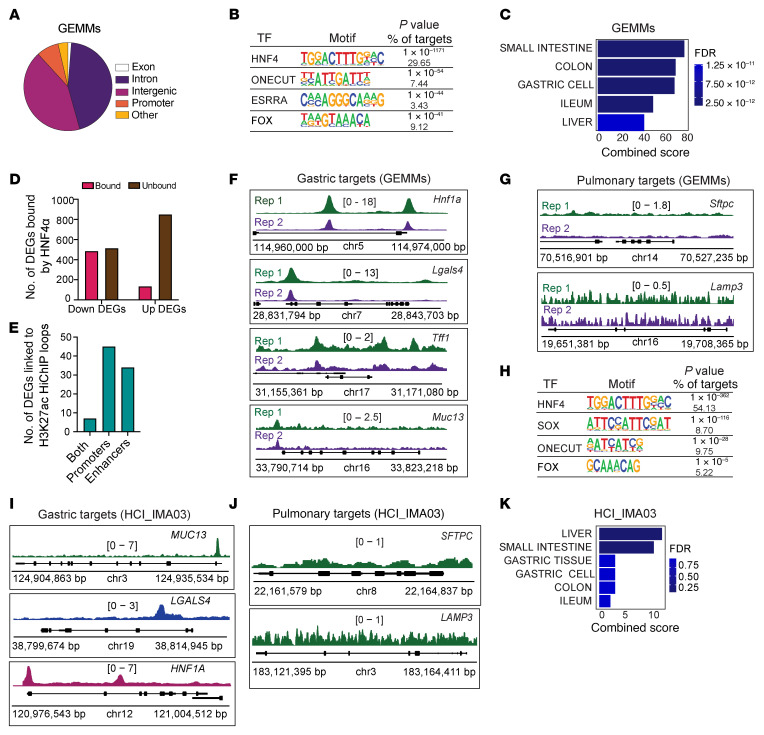
HNF4α directly binds and activates gastric lineage programs in IMA. (**A**) Genome-wide HNF4α ChIP-seq occupancy in KN tumors. (**B**) HOMER motif enrichment of HNF4α-bound peaks in KN tumors. (**C**) ENRICHR ARCHS4 tissue enrichment of genes annotated from HNF4α peaks in KN tumors. (**D**) Overlap between bulk RNA-seq DEGs and genes annotated from HNF4α peaks in GEMM tumors. Down, downregulated; Up, upregulated. (**E**) Distribution of HNF4α-bound peaks defined by integration of HNF4α ChIP-seq and H3K27ac HiChIP and annotated to the 86 genes shared with in vivo downregulated DEGs, stratified by looping to promoters, enhancers, or both. (**F** and **G**) Integrative Genome Visualization (IGV) tracks showing HNF4α occupancy at gastric lineage genes (**F**) and lack of occupancy at pulmonary lineage genes (**G**) in KN tumors. (**H**) HOMER motif enrichment of HNF4α-bound peaks in HCI_IMA03. (**I** and **J**) IGV tracks showing HNF4α occupancy at gastric lineage genes (**I**) and lack of occupancy at pulmonary lineage genes (**J**) in HCI_IMA03. (**K**) ENRICHR ARCHS4 tissue enrichment of genes annotated from HNF4α peaks in HCI_IMA03.

ChIP-seq for HNF4α in GFP^+^ nuclei sorted from KN GEMM tumors identified 4,021 high-confidence binding sites across 2 biological replicates (Supplemental Table 1). These peaks were predominantly located within intronic, intergenic, and promoter regions (Figure 2A). Motif analysis of these binding sites revealed expected enrichment for HNF4, as well as motifs bound by transcription factor families that regulate endodermal differentiation such as ONECUT, ESRRA, and FOX (Figure 2B). FoxA1 and FoxA2 regulate GI differentiation (52, 53), and the ONECUT family is crucial for embryonic development of liver, pancreas, and neurons (54). ERRα (encoded by *ESRRA*) modulates metabolic processes in GI tissues in cooperation with HNF4α (55, 56). Furthermore, ENRICHR analysis (57, 58) using ARCHS4 tissue annotations demonstrated that HNF4α-bound genes were enriched in signatures of GI tissues (Figure 2C).

To assess the functional consequences of HNF4α binding, we performed bulk RNA-seq on GFP^+^ tumor cells isolated from KN and KNH GEMMs (*n* = 4 mice per genotype), identifying 1,903 differentially expressed genes (DEGs) (log_2_ fold change [FC] > 0.585, adjusted *P* [*P*adj] < 0.05; Supplemental Table 2). Integration of ChIP-seq and RNA-seq revealed that approximately 50% of genes downregulated (Down DEGs) and approximately 15% of genes upregulated (Up DEGs) in KNH tumors were direct HNF4α targets, consistent with HNF4α functioning predominantly as a direct transcriptional activator in IMA (Figure 2D).

To more precisely link HNF4α binding sites with target genes, we intersected these data with H3K27ac HiChIP datasets from KN GEMM tumors (28), enabling identification of HNF4α-bound regulatory regions that physically looped to promoters, enhancers, or both. Of the 437 unique genes annotated from the H3K27ac HiChIP dataset, 275 were associated with HNF4α-bound regions in vivo, and 86 of these genes were significantly downregulated(log2 fold change [FC] > 0.585, adjusted P [Padj] < 0.05) following Hnf4a deletion, as determined by bulk RNA-seq (Figure 2E and Supplemental Table 3). These findings provide direct evidence that HNF4α regulates gene expression through enhancer-promoter interactions at lineage-specific regulatory elements in IMA.

Inspection of individual genes from KN GEMM tumors showed strong HNF4α binding at regulatory elements of canonical gastric genes including *Hnf1a*, *Lgals4*, *Tff1*, and *Muc13* but not pulmonary targets including *Sftpc* and *Lamp3* (Figure 2, F and G). Importantly, HNF4α ChIP-seq in an IMA patient-derived organoid (HCI_IMA03) recapitulated gastric-associated motif enrichment and target occupancy (Figure 2, H–K, and Supplemental Figure 2, A–C). Similar HNF4α binding profiles were also observed in 2 mouse-derived IMA organoid lines (429A and 1311G) (Supplemental Figure 2, D–L, and Supplemental Table 1). Together, these data demonstrate that HNF4α chromatin occupancy in murine IMA mirrors the human disease, with no detectable binding at pulmonary marker genes. This gastric-restricted occupancy pattern contrasts with hybrid identity LUAD, where HNF4α colocalizes with NKX2-1 at pulmonary loci (59).

**Figure 3:**
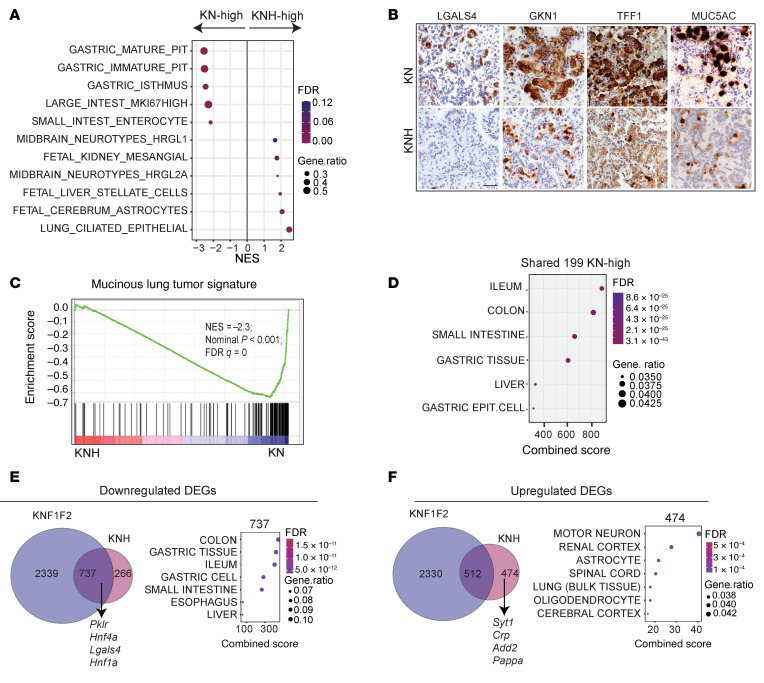
HNF4α maintains gastric differentiation and suppresses transcriptional plasticity in IMA. (**A**) GSEA of C8 cell-type signatures based on DEGs in KNH versus KN GEMM tumors. (**B**) Representative IHC of downstream effectors of HNF4α in KN and KNH GEMM tumors at 14 weeks PTI. Scale bar: 100 μm. (**C**) GSEA of the IMA mucinous tumor signature (PMID: 28255028) using DEGs from bulk RNA-seq of GEMM tumors. The normalized enrichment score (NES) and FDR are shown. (**D**) ENRICHR ARCHS4 analysis of 199 genes shared between KN-high DEGs and HNF4α-induced genes in H2122 cells. (**E** and **F**) Venn diagrams showing an overlap of downregulated (**E**) and upregulated (**F**) DEGs between KNF1F2 and KNH GEMM tumors. (**E**) Downregulated DEGs (KNF1F2, *n* = 3,076; KNH, *n* = 1,003) with 737 shared genes (hypergeometric test, *P* < 1 × 10^–15^; ~5-fold enrichment) and ENRICHR ARCHS4 tissue enrichment of shared genes. (**F**) Upregulated DEGs (KNF1F2, *n* = 2,842; KNH, *n* = 986) with 512 shared genes (*P* < 1 × 10^–194^; ~3.8-fold enrichment) and 474 genes uniquely upregulated in KNH with ENRICHR ARCHS4 enrichment.

To identify HNF4α-regulated identity programs in IMA, we performed gene set enrichment analysis (GSEA) (60, 61) of GEMM tumor–derived DEGs against cell-type signatures. Gastric lineage signatures, including immature and mature pit cells and isthmus cells, were strongly depleted in KNH tumors relative to KN tumors, whereas nongastric programs, including astrocytic, neuronal, and liver stellate cell signatures (nonepithelial), were upregulated (Figure 3A, Supplemental Figure 3A, and Supplemental Table 4). Consistent with these transcriptional changes, IHC analysis revealed reduced expression of the pan-gastric marker galectin 4 (LGALS4) and the pit cell markers GKN1, trefoil factor 1 (TFF1) and mucin 5 subtype AC (MUC5AC) in KNH tumors (Figure 3B). *Hnf4a* deletion caused a significant decline in an IMA gene expression signature derived from human tumors (62) (Figure 3C). We also compared KN-high DEGs (defined as genes expressed at significantly higher levels in KN tumors relative to KNH; log_2_FC > 0.585, *P*adj < 0.05, Supplemental Table 2) with genes induced by exogenous HNF4α expression in the NKX2-1/HNF4α dual-negative H2122 human lung carcinoma cells (43). We identified 199 shared genes, which were enriched for gastric-related cell lineages when compared with the All RNA-seq and ChIP-seq sample and signature search ARCHS4 resource via ENRICHR analysis (Figure 3D). Taken together, these data show that DEGS identified in GEMMs are relevant to human IMA.

Bulk RNA-seq of KN and KNH organoids demonstrated significant overlap with GEMM tumors. Bulk RNA-seq of 429A and 1311G organoids revealed 818 and 2,323 DEGs, respectively, between KN and KNH genotypes (log_2_FC > 0.585, *P*adj < 0.05, Supplemental Tables 5 and 6). The 429A organoid model showed the highest concordance with GEMM tumor–derived DEGs, with 107 KNH-high genes (39%) and 202 KN-high genes (60%) overlapping with upregulated genes in KNH and KN GEMM tumors, respectively. The 1311G organoid line showed more modest overlap, with 131 KNH-high (12%) and 284 KN-high (23%) shared with GEMM tumor–derived DEGs (Supplemental Figure 3, B–F). GSEA of organoid DEGs from both murine models mirrored GEMM tumors, showing depletion of gastric pit cell identity and activation of nongastric signatures in the KNH tumors (Supplemental Figure 4, A–B, and Supplemental Table 7).

Although intestine-associated transcripts such as *Vil1*, *Tff3*, and *Pdx1* were detected in KN tumors, IHC did not support overt intestinal differentiation. CDX2 was undetectable, and PDX1, which is physiologically expressed in the gastric antrum in addition to the duodenum (63), was restricted to a minor tumor subset that declined following *Hnf4a* deletion (Supplemental Figure 4, C and D). Moreover, the HNF4α isoform expression pattern of IMA (P2-high) (42) is more similar to normal stomach than intestines, which coexpress P1 and P2 (64). These findings support a predominantly gastric identity with limited, CDX2-independent intestinal features rather than bona fide intestinal differentiation and establish HNF4α as a central regulator of gastric lineage fidelity in IMA, maintaining pit cell identity while constraining alternative lineage programs.

Our prior work showed that FoxA1/2 are required for the gastric differentiation in IMA, including activation of *Hnf4a* expression in IMA (28). To define the subset of FoxA1/2-dependent genes that are HNF4α dependent, we compared DEGs in KNH versus KN tumors with those in *Foxa1/2* double-KO tumors (KNF1F2) verses KN tumors (28). The 737 shared downregulated genes were enriched in GI pathways (Figure 3E), and this overlap was highly significant by hypergeometric test (*P* < 1 × 10^–15^; ~5-fold enrichment) (65). Taken together with prior studies showing that FoxA1/2 bind the *Hnf4a* locus in KN tumors and are required for *Hnf4a* expression and activation of the gastric program (11, 14, 28), these data support a model in which HNF4α serves as a major downstream effector of FoxA1/2 in gastric identity specification in IMA. Intriguingly, we identified 474 genes uniquely upregulated in KNH but not in KNF1F2, many of which were associated with nongastric lineages, suggesting that FoxA1/2 are required to activate nongastric identity programs in KNH tumors (Figure 3F).

Finally, pathway analysis of GEMM tumor–derived DEGs showed that loss of HNF4α also altered metabolic gene programs. KN tumors were enriched for pathways related to xenobiotic metabolism, steroid biosynthesis, glycolysis, oxidative phosphorylation, and bile acid metabolism (Kyoto Encyclopedia of Genes and Genomes [KEGG], Hallmark; Supplemental Figure 4, E and F, and Supplemental Table 8), whereas KNH tumors were enriched for cilium assembly and organelle biogenesis (Reactome; Supplemental Figure 4G and Supplemental Table 8). These patterns were consistent with gene ontology of organoid-derived DEGs (Supplemental Table 9) and align with established roles of HNF4α in regulating metabolic programs in GI tissues and liver (66–68). Together, these data indicate that HNF4α is a central regulator of gastric identity in IMA through coordinated control of differentiation-associated and metabolic gene programs.

### HNF4α restricts cellular heterogeneity and prevents lineage deviation in IMA.

Intercellular heterogeneity contributes to lineage plasticity and therapeutic resistance in LUAD, including IMA (5, 17, 69). Although bulk RNA-seq revealed that *Hnf4a* deletion suppresses gastric identity and activates nongastric programs, it lacks single-cell resolution and obscures transcriptional heterogeneity.

**Figure 4:**
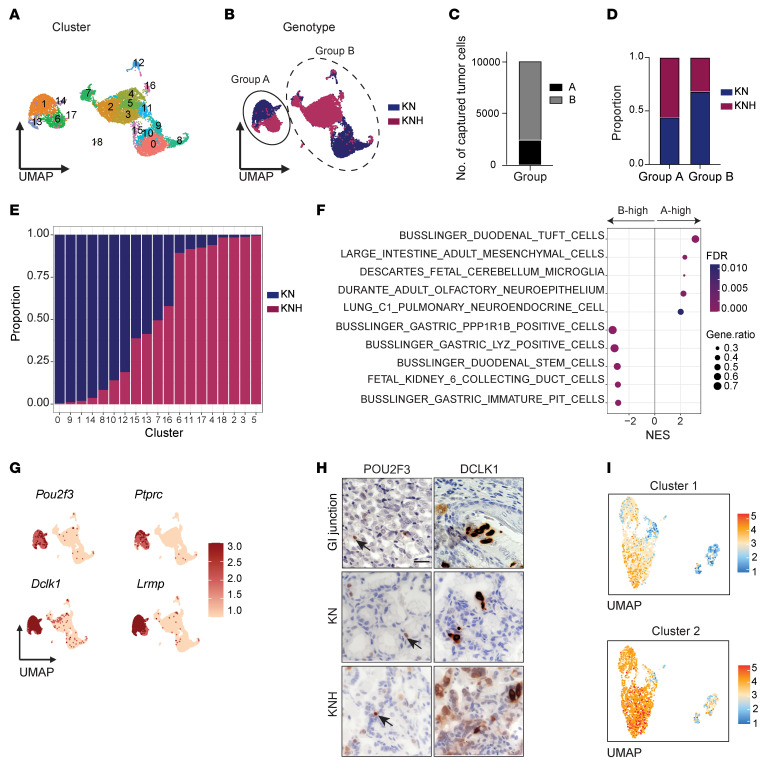
HNF4α constraints cellular heterogeneity in IMA at single-cell resolution. (**A**) Uniform manifold approximation and projection (UMAP) of malignant KN (5,001 cells) and KNH (5,024 cells) GEMM tumors (*n* = 2 mice per genotype, multiple tumors per mouse) colored by Seurat clusters. (**B**) UMAP of KN and KNH tumor cells colored by genotype. (**C**) Total tumor cells in groups A and B. (**D**) Proportion of KN and KNH tumor cells in groups A and B. (**E**) Distribution of tumor cells from each genotype across Seurat clusters. (**F**) GSEA of C8 cell-type signatures using DEGs comparing groups A and B. (**G**) UMAPs of tuft cell marker genes (*Pou2f3*, *Ptprc, Dclk1,* and *Lrmp*). (**H**) Representative IHC images of DCLK1 and POU2F3 staining in KN and KNH GEMM tumors at 14 weeks PTI. Scale bar: 50 μm. Arrows indicate POU2F3-positive tumor cells. (**I**) UMAP showing enrichment of neuron-like (cluster 1) and immune-like (cluster 2) tuft-like cells in KN and KNH tumors in group A (PMID: 29144463).

To define the role of HNF4α across IMA subpopulations, we performed scRNA-seq on sorted GFP^+^ tumor cells from KN and KNH GEMMs at 14 weeks PTI (*n* = 2 mice per genotype, Supplemental Figure 5, A–D). Analysis of all high-quality cells demonstrated that most were bona fide tumor cells, defined by coexpression of *CreERT2* and *sfGFP* (Supplemental Figure 5, E and F). After excluding normal cells (*sfGFP/CreERT2*^–^), we identified 18 clusters comprising 5,003 KN and 5,024 KNH cells (Figure 4A, and Supplemental Table 10). These segregated into 2 major groups: group A, with equal contributions from KN and KNH cells, and group B (~80% of tumor cells) enriched for KN cells (Figure 4, B–E). A small fraction (< 2%) of unrecombined *Nkx2-1* cells retained an AT2-like state (70), expressing canonical markers including *Sftpa1*, *Sftpc*, and *Nkx2-1* (Supplemental Figure 5, G and H). Quantification of exon-specific reads confirmed efficient *Hnf4a* deletion in KNH cells (Supplemental Figure 5I and Supplemental Table 10).

Differential expression analysis identified 2,958 DEGs between groups A and B (1,580 group A–high, and 1,378 group B–high; log_2_FC > 0.25; Supplemental Table 10). Group A exhibited a tuft-like program marked by *Pou2f3*, *Dclk1*, *Ptprc*, and *Lrmp* with similar expression levels in KN and KNH tumors confirmed by scRNA-seq and IHC (Figure 4, F–H). Consistent with this, *Hnf4a* deletion did not alter the expression of neuronal or immune-associated tuft markers (Figure 4I), indicating preservation of this subpopulation in IMA.

Despite similar tuft marker expression levels, KN and KNH cells in group A clustered distinctly, with 975 DEGs between the genotypes (log_2_FC > 0.25; Supplemental Table 10). After excluding genes that were also differentially expressed in group B, we identified 467 KN-high and 281 KNH-high genes unique to group A (Supplemental Figure 5J). Enrichment analysis revealed reduced tuft, microglia, and neuronal signatures in KNH cells, driven by decreased expression of tuft-associated genes including *IL17rb* (71), *Hpgds* (72), *Alox5* (73), and *Plcg2* (72) (Supplemental Figure 5, K–M). These data suggest that HNF4α regulates a subset of tuft-associated genes without altering tuft-like identity.

**Figure 5:**
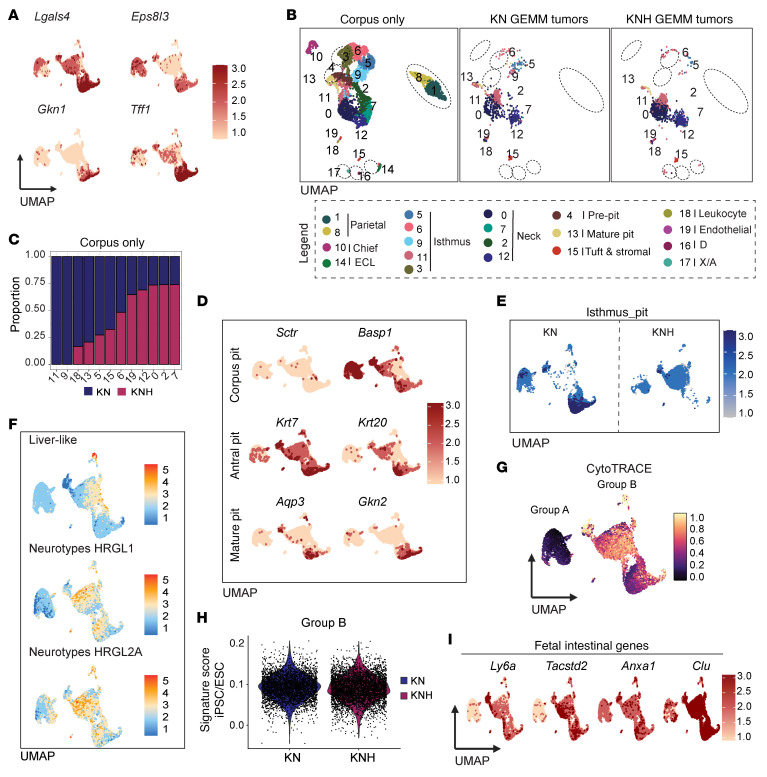
HNF4α sustains a stomach-like gastric lineage program in IMA at single-cell resolution. (**A**) UMAPs of representative HNF4α target genes (*Lgals4*, *Eps8l3*, *Gkn1*, and *Tff1*). (**B**) UMAPs of the Takada et al. corpus dataset (PMID: 37386010), with KN and KNH tumor cells mapped by Seurat label transfer. (**C**) Proportion of KN and KNH tumor cells mapping to corpus clusters. (**D**) Feature plots of corpus pit, antral pit, and mature pit cell markers in IMA tumor cells. (**E**) UMAPs of gene module scores along the isthmus-to-pit trajectory. (**F**) UMAPs of gene modules associated with alternative cell types in KNH tumors. (**G**) UMAP of CytoTRACE1 scores (0–1), with lower values indicating greater differentiation. (**H**) Violin plot of iPSC and ESC gene signatures in KN and KNH tumor cells (group B). (**I**) UMAPs of fetal intestinal stem cell markers (*Ly6a*, *Tacstd2*, *Anxa1*, and *Clu*).

We next evaluated group B, which was enriched for gene signatures associated with gastric pit cell differentiation. HNF4α target genes identified by bulk RNA-seq analysis, including the gastric markers Lgals4, Eps8l3, Gkn1, and Tff1, were significantly downregulated in group B KNH cells (log2 fold change [FC] > 0.585, adjusted P [Padj] < 0.05; Figure 5A). To assess the extent to which KN and KNH cells recapitulate normal gastric differentiation, we projected our scRNA-seq data onto reference atlases of gastric epithelial cell types (22). In the corpus-only atlas, KN cells mapped primarily to mature pit (cluster 13), neck (clusters 0, 2, 7, and 12), and isthmus (clusters 5, 6, 9, and 11) cell types, with limited representation of tuft cells (cluster 15) (Figure 5, B and C), and showed minimal mapping to parietal, chief, or enteroendocrine cells. In contrast, KNH cells were underrepresented in pit and isthmus cell populations and preferentially mapped to neck cell clusters. Similar shifts in cell-type representation were observed using a second atlas incorporating both corpus and antrum lineages (Supplemental Figure 6, A and B). Although we previously observed rare PDX1^+^ KN tumor cells, these cells lacked CDX2 expression, arguing against bona fide duodenal or intestinal differentiation. Consistent with this, unbiased comparison of tumor scRNA-seq profiles with murine reference cells showed that KN cells did not map to duodenal cell populations (Supplemental Figure 6, A and B).

Analysis of lineage-specific marker genes and cell-type signatures (74) demonstrated a marked loss of pit and isthmus identity in group B KNH cells (Figure 5D and Supplemental Figure 6, C and D). To further assess differentiation dynamics, we examined established markers of the isthmus-to-pit transition (22), which revealed a reduction in genes associated with progression toward mature pit cell states, indicating a requirement for HNF4α in this lineage commitment step (22, 75) (Figure 5E). Although KNH cells were more frequently assigned to neck cell clusters, the overall neck cell signature was comparable between genotypes, likely reflecting shared expression of select markers such as *Tff2* in both neck and pit cells. Together, these findings suggest that KNH cells primarily undergo loss of pit and isthmus identity rather than a bona fide switch to a neck cell fate (Supplemental Figure 6E).

Because mucous neck cell gene expression overlaps substantially with spasmolytic polypeptide–expressing metaplasia (SPEM), a metaplastic lineage associated with gastric injury repair (76), we evaluated whether the transcriptional changes following *Hnf4a* deletion were consistent with SPEM activation, rather than reflecting a shift among gastric epithelial cell lineages. We identified no enrichment of SPEM-associated markers or gene signatures in KNH cells (Supplemental Figure 6, F and G), indicating that loss of HNF4α does not induce a SPEM-like transcriptional program in IMA.

Finally, we asked whether the nongastric identities observed in KNH cells by bulk RNA-seq data are detectable at the single-cell level. Group B KNH cells exhibited enrichment of curated nongastric signatures, including neuronal and liver-like programs (Figure 5F). These signatures encompassed both canonical marker genes (*Fgfr3*, *Kcnh7*, and *Rora* for neuronal; *Mgst1*, *Trf*, and *Cp* for liver) and broader cell-type–associated transcriptional networks (Supplemental Figure 6H). Moreover, group B KNH cells exhibited a greater number of distinct expressed genes per cell than did group B KN cells by CytoTRACE analysis (77), a computational metric that correlates with cellular differentiation potential (Figure 5G). In contrast, group A (tuft-like) cells had low transcriptional diversity that was unaffected by *Hnf4a* deletion. This analysis shows that HNF4α loss caused increased transcriptional diversity, which typically correlates with loss of differentiation, specifically in pit-/isthmus-like IMA cells, but not in tuft-like IMA cells.

To test whether this change in differentiation reflected reactivation of fetal or pluripotent transcriptional programs in group B KNH cells, we analyzed the expression of induced pluripotent stem cell/embryonic stem cell (iPSC/ESC) gene signatures (78) and canonical fetal intestinal stem cell markers (*Ly6a*, *Tacstd2*, *Anxa1*, *Clu*) (79). All were similarly expressed in KN and KNH cells in group B, indicating that the altered differentiated state in KNH cells likely does not reflect activation of a fetal-like or pluripotent program (Figure 5, H and I).

Taken together, these data show that HNF4α regulated shared and distinct transcriptional programs in major (pit-/isthmus-like) and minor (tuft-like) IMA subpopulations. *Hnf4a* deletion disrupted pit-like identity and induced nongastric lineage signatures in the major subpopulation, while the tuft-like subpopulation remained relatively stable despite loss of a subset of HNF4α-dependent, tuft-associated transcripts.

### HNF4α loss reprograms FoxA1/2 binding to drive nongastric states in IMA.

Having established that HNF4α promotes IMA growth, activates gastric differentiation, and restricts single-cell transcriptional plasticity, we next investigated how its loss promotes nongastric lineage programs, including neuronal and liver-like states. FoxA1/2 are diffusely expressed in both KN and KNH cells (Supplemental Figure 7, A and B), and bulk RNA-seq across IMA models demonstrated that nongastric program activation after HNF4α loss was partially FoxA1/2 dependent (Figure 3F). We therefore hypothesize that, in the absence of HNF4α, FoxA1/2 engage de novo chromatin sites to activate nongastric programs and rewire cell identity.

To test this, we performed ChIP-seq for FoxA1/2 in GFP-positive nuclei sorted from lung tumors of KN and KNH GEMMs. HOMER motif analysis of FoxA1 peaks identified enrichment of the FOXA motif in both KN and KNH tumors, whereas HNF4α motifs were enriched only in KN tumors (Supplemental Figure 7C and Supplemental Table 1). A similar pattern was observed for FoxA2 peaks (Supplemental Figure 7D and Supplemental Table 1). FoxA1/2 co-occupied HNF4α-bound sites in KN tumors, whereas in KNH tumors they remained co-occupied with each other (Supplemental Figure 7, E–G). At the *Lgals4* locus, FoxA1, FoxA2, and HNF4α co-bound in KN tumors (Supplemental Figure 7H). FoxA1 ChIP-seq in 1311G KN and KNH organoids recapitulated these in vivo binding patterns (Supplemental Figure 7, I–L).

**Figure 6:**
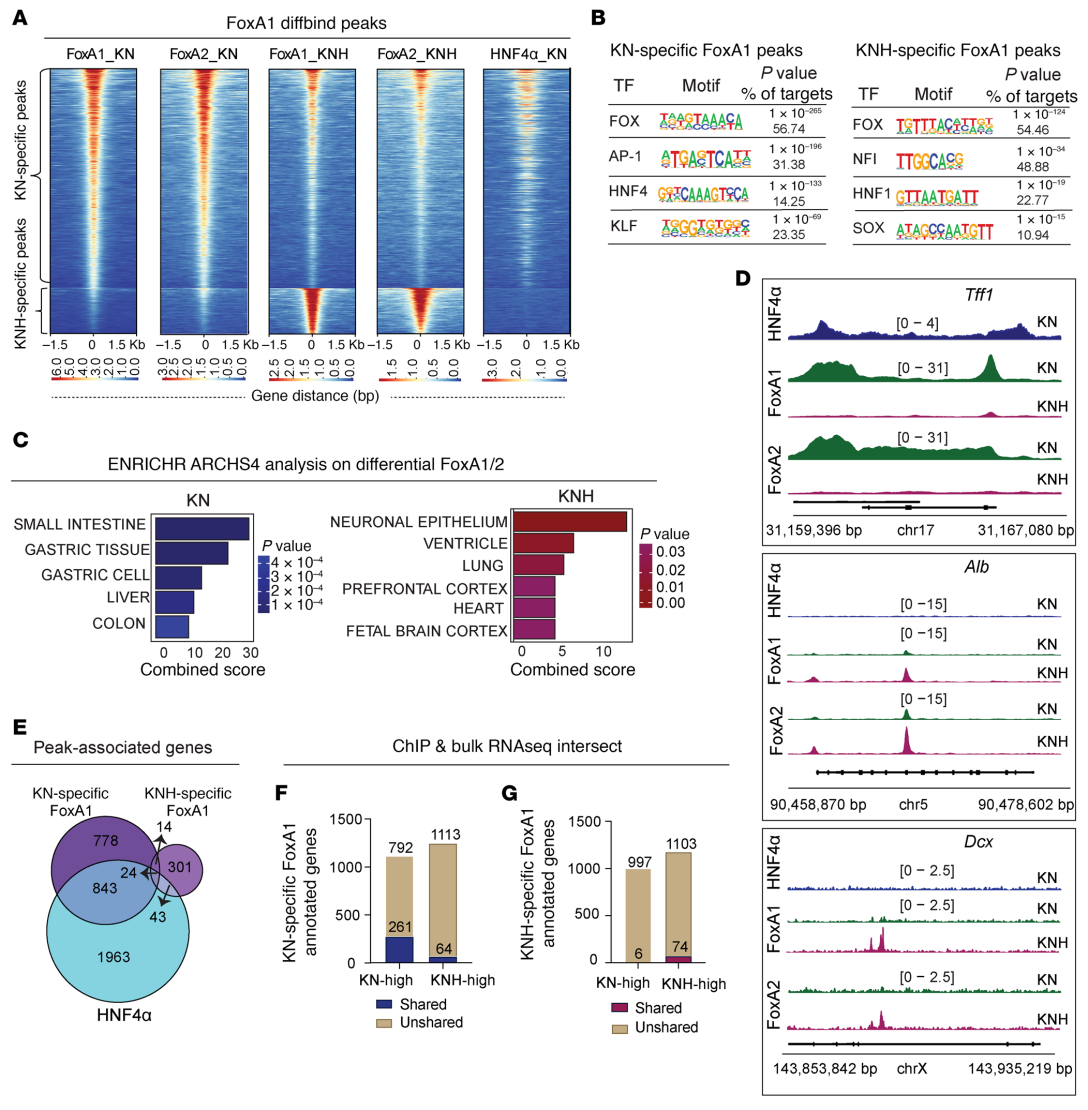
HNF4α loss reprograms FoxA1/2 binding to drive nongastric states in IMA. (**A**) Heatmap of differential FoxA1 binding between KN and KNH GEMM tumors (adjusted *P* < 0.05). (**B**) HOMER motif enrichment at differential FoxA1 peaks in KN and KNH GEMM tumors. (**C**) ENRICHR ARCHS4 tissue enrichment of genes annotated from shared differential FoxA1/2 peaks (left, KN-specific; right, KNH-specific). (**D**) IGV tracks showing reduced FoxA1/2 binding at the gastric gene locus *Tff1* and increased binding at nongastric loci (*Dcx* and *Alb*) in KNH GEMM tumors. (**E**) Overlap between genes associated with KN-specific FoxA1 peaks and HNF4α-bound regions in KN GEMM tumors. (**F** and **G**) Overlap of genes annotated from KN-specific (**F**) or KNH-specific (**G**) FoxA1 peaks and DEGs identified by bulk RNA-seq in GEMM tumors. *P* values in **B** and **C** were calculated by HOMER using a hypergeometric test for motif enrichment relative to background sequences.

Using DiffBind, we identified 2,369 KN-specific and 494 KNH-specific in vivo FoxA1 peaks, and 1,443 KN-specific and 983 KNH-specific in vivo FoxA2 peaks (Supplemental Table 11). Mapping these differential FoxA1 peaks onto the merged binding sets for HNF4α (in KN GEMM tumors) and FoxA1/2 (in KN and KNH GEMM tumors) revealed striking patterns: KN-specific FoxA1 peaks overlapped substantially with FoxA1, FoxA2, and HNF4α sites in KN tumors, while KNH-specific peaks overlapped with FoxA1 and FoxA2 in KNH tumors but did not overlap with HNF4α (Figure 6A). We made similar observations for FoxA2 (Supplemental Figure 8A and Supplemental Table 11).

HOMER motif analysis revealed that KN-specific FoxA1 peaks were enriched for FOX, AP-1, and HNF4 motifs, whereas KNH-specific peaks were enriched for FOX, NFI, HNF1, and SOX motifs (Figure 6B). The presence of HNF4, AP-1, and KLF motifs in KN-specific peaks suggests that FoxA1 cooperates with these factors to drive gastric identity in IMA (27, 80, 81). In contrast, the enrichment of NFI and SOX motifs in KNH-specific peaks suggest that, in the absence of HNF4α, FoxA1/2 may cooperate with alternative transcriptional partners to drive lineage plasticity and activate nongastric programs (82–84). FoxA2 motif analysis showed a similar pattern (Supplemental Figure 8, B and C).

ENRICHR cell-type enrichment analysis of genes annotated from KN- or KNH-specific FoxA1 or FoxA2 peaks showed that genes bound by KN-specific FoxA1 or FoxA2 peaks were enriched for gastric epithelial cell lineages, whereas those bound by KNH-specific FoxA1 or FoxA2 peaks were linked to neuronal and other nongastric cell types (Supplemental Figure 8, D and E). Similar results were obtained when we analyzed genotype-specific shared differential FoxA1/2 binding sites (Figure 6C). For example, FoxA1/2 binding at the pit cell marker *Tff1* was lost in KNH tumors, whereas binding increased at representative neuronal doublecortin (*Dcx)* and hepatic albumin (*Alb)* marker genes (Figure 6D). Notably, *Alb* expression was maintained in KNH tumors despite HNF4α loss, indicating that its activation in this context was not strictly dependent on HNF4α. This intriguing result is consistent with prior work demonstrating that in hepatocytes, FoxA2 can sustain *Alb* expression when HNF4α is lost through alternative transcriptional factors supporting a context-dependent transcriptional rewiring (85). Together, these data indicate that HNF4α loss reshapes enhancer selection, enabling FoxA1/2-driven activation of nongastric lineage programs in KNH tumors.

These binding changes were accompanied by corresponding shifts in gene expression: genes associated with KN-specific FoxA1 peaks were significantly downregulated in KNH tumors (|log2FC| > 0.585, Padj < 0.05 by bulk RNA-seq), while those linked to KNH-specific FoxA1 peaks were upregulated (Figure 6, E–G). Similar transcriptional correlations were observed for FoxA2 (Supplemental Figure 8, F–H).

During endodermal development, FoxA recruitment to a subset of enhancers with weaker, less abundant motifs is dependent on other tissue-specific transcription factors (86). We therefore asked whether differences in motif strength or abundance could account for the dependence of a subset of FoxA1/2 binding sites on HNF4α in IMA. Dynamic FoxA1 sites in both KN and KNH tumors exhibited reduced motif abundance and weaker motif strength compared with static peaks, whereas dynamic KNH FoxA2 sites showed reduced motif abundance without significant changes in motif strength (Supplemental Figure 9, A–D, and Supplemental Table 12). Canonical HNF4 motifs were more abundant and stronger within KN-specific FoxA1/2 regions compared with static or KNH-specific FoxA1/2 regions (Supplemental Figure 9, E–H, and Supplemental Table 12). These data suggest that differences in FoxA motif strength and abundance may contribute to the subset of FoxA1/2 binding sites in IMA that depend on HNF4α expression.

DiffBind analysis of FoxA1 ChIP-seq in 1311G KN and KNH organoids revealed extensive FoxA1 relocalization in KNH tumors, from canonical gastric loci, including HNF4α target genes bound in KN, to nongastric loci associated with neuronal cell identities (Supplemental Figure 10, A–D). These changes in FoxA1 occupancy mirrored the enrichment of nongastric programs observed in KNH tumors across both scRNA-seq and bulk RNA-seq datasets (Figure 3A and Figure 5F).

### HNF4α loss alters chromatin accessibility at lineage-specific sites in IMA.

**Figure 7:**
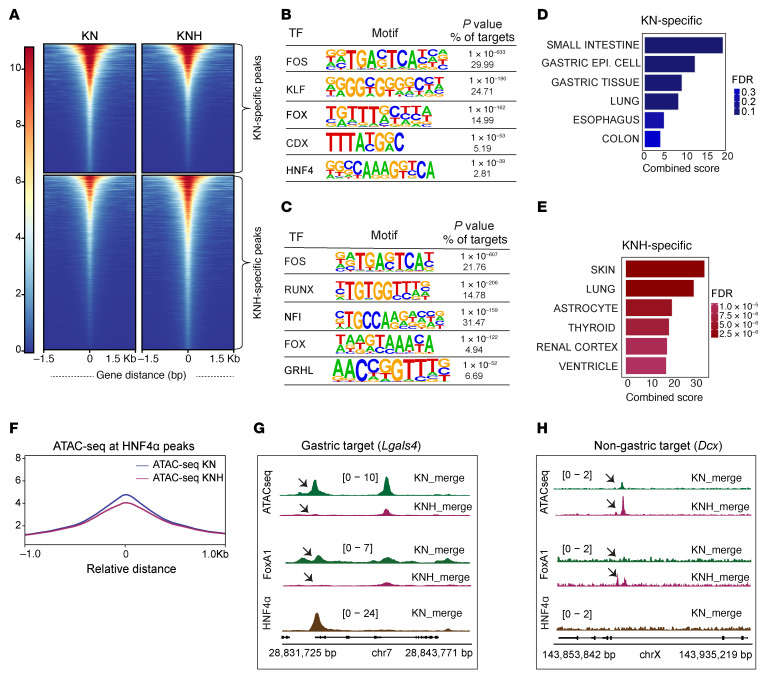
HNF4α loss induces selective chromatin remodeling and FOXA redistribution in IMA organoids. (**A**) Heatmap of differential chromatin accessibility between 1311G KN and KNH organoids (adjusted *P* < 0.05). (**B** and **C**) HOMER motif enrichment at KN-specific (**B**) and KNH-specific (**C**) ATAC-seq peaks in 1311G. (**D** and **E**) ENRICHR ARCHS4 tissue enrichment of genes annotated from KN-specific (**D**) and KNH-specific (**E**) ATAC-seq peaks in 1311G. (**F**) Mean ATAC-seq signal in 1311G KN and KNH centered on HNF4α summits defined in 1311G KN. (**G** and **H**) IGV tracks at the *Lgals4* (**G**) and *Dcx* (**H**) loci showing ATAC-seq and HNF4α/FoxA1 ChIP-seq signal in 1311G KN and KNH organoids. *P* values in **B** and **C** were calculated by HOMER using a hypergeometric test for motif enrichment relative to background sequences.

To determine whether HNF4α regulates chromatin accessibility in IMA, we performed the assay for transposase-accessible chromatin using sequencing (ATAC-seq) in 1311G organoids. We identified 2,984 KN-specific and 3,530 KNH-specific accessible regions (Figure 7A), indicating structured chromatin remodeling following *Hnf4a* deletion. Motif analysis revealed enrichment of KLF, FOX, CDX, and HNF4 motifs in KN-specific regions and RUNX, NFI, FOX, and GRHL motifs in KNH-specific regions (Figure 7, B and C). The presence of the FOX motif in both KN- and KNH-specific sites likely reflects redistribution of FoxA1 following HNF4α loss. GSEA of peak-annotated genes further demonstrated that KN-specific regions were enriched for gastric epithelial cell types, whereas KNH-specific regions mapped predominantly to nongastric cell lineages (Figure 7, D and E).

We next evaluated changes in accessibility at the summits of the 3,057 HNF4α binding sites in 1311G KN organoids. Overall accessibility only declined slightly at these sites upon *Hnf4a* deletion (Figure 7F). Only 98 summits overlapped differentially accessible regions, the majority of which (91 sites) corresponded to KN-specific elements that lost accessibility in KNH tumors, while 2,959 sites remained stable (Supplemental Figure 11A). Consistent with these observations, the HNF4 motif was only observed in 3% of KN-specific sites.

These observations suggest that transcription factors other than HNF4α are the primary drivers of chromatin accessibility changes following *Hnf4a* deletion. Notably, FOX motifs were present in a greater fraction of differentially accessible regions than HNF4 motifs. Integration of ATAC-seq with FoxA1 ChIP-seq and bulk RNA-seq data further supported a role for FoxA1 in coordinated regulatory remodeling after HNF4α loss. KNH-specific accessible regions preferentially overlapped KNH-specific FoxA1 binding and genes upregulated in KNH tumors, whereas KN-specific accessible regions aligned with KN-specific FoxA1 binding and genes upregulated in KN tumors (Supplemental Figure 11, B and C). Cross-platform integration of peak-associated genes from ATAC-seq, ChIP-seq, and bulk RNA-seq identified 106 high-confidence KN targets and 24 KNH targets, defining distinct lineage-specific regulatory programs (Supplemental Figure 11, D and E). Representative loci further illustrated this remodeling, showing concomitant loss of HNF4α binding, FoxA1 binding, and chromatin accessibility at the gastric gene *Lgals4* in KNH tumors, together with increased accessibility and FoxA1 binding at the neuron-associated gene *Dcx* (Figure 7, G and H). Together, these findings suggest that HNF4α loss reshaped the chromatin landscape in IMA, in part by enabling FoxA1 redistribution from gastric to nongastric regulatory elements.

### HNF4α dampens the response of IMA to KRAS inhibition.

**Figure 8:**
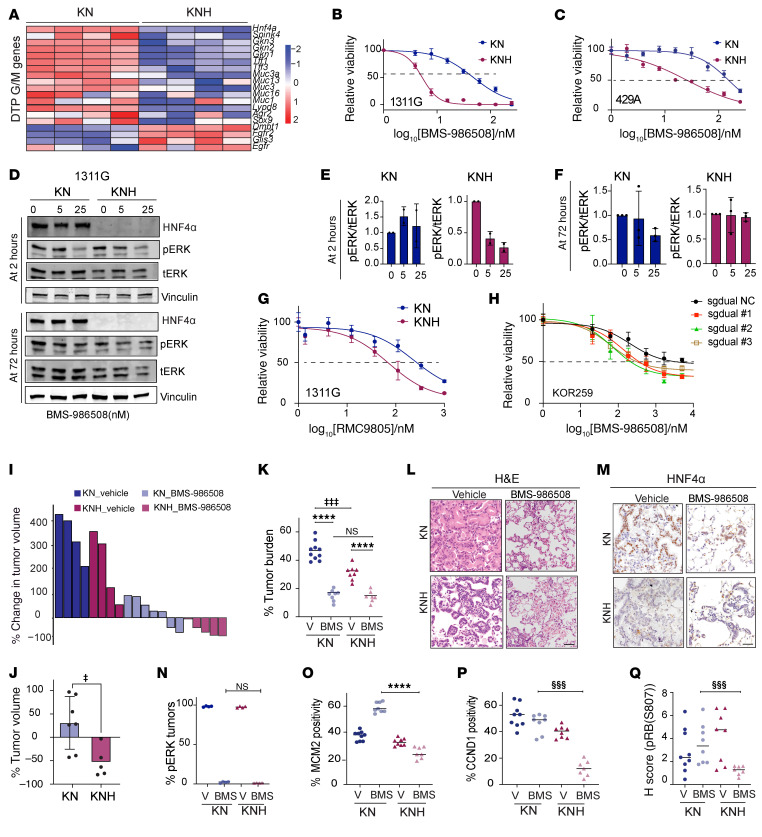
HNF4α sustains DTP cells and limits the response to KRAS^G12D^ inhibition. (**A**) Heatmap showing log_2_-normalized expression for G/M genes associated with DTP cells in IMA in vivo. (**B** and **C**) Dose-response curves for IMA organoids treated with BMS-986508. (**B**) 1311G (IC_50_: KN = 47.77 nM; KNH = 5.27 nM) and (**C**) 429A (IC_50_: KN = 152.3 nM; KNH = 25.61 nM). IC_50_ values were calculated by nonlinear regression. Data represent 1 of 3 independent biological replicates and are presented as the mean ± SEM. (**D**–**F**) Representative immunoblot analysis of the indicated proteins in 1311G organoids treated with BMS-986508 for 2 hours (*n* = 2 independent biological replicates) or 72 hours (*n* = 3), with quantification of pERK normalized to total ERK (tERK). Data are presented as the mean ± SD. (**G**) Dose-response curves for 1311G organoids treated with RMC-9805 (IC_50_: KN = 234.3 nM; KNH = 71.17 nM). Data represent 1 of 3 independent biological replicates and are presented as the mean ± SEM. (**H**) Human-derived organoids (KOR259) transduced with lentiviral dual gRNAs (sgdual) targeting HNF4A P1/P2 isoforms or a nontargeting control (NC), followed by 4 days of selection and treatment with BMS-986508 for 72 hours. IC_50_ values were 20.5 nM, 13.8 nM, 7.3 nM, and 6.4 nM for the NC and sgdual 1–3, respectively. Data represent 1 of 3 independent biological replicates and are presented as the mean ± SEM. (**I**) Waterfall plot showing the fold change in tumor volume in 1311G KN and KNH allografts treated with vehicle or BMS-986508. (**J**) Endpoint tumor volumes from **I**. ^‡^*P* = 0.0126, by unpaired, 2-tailed Student’s *t* test. (**K**) Tumor burden in KN and KNH GEMM tumors after 2 weeks of treatment with vehicle or BMS-986508. NS, *P* = 0.6943; ^‡^^‡^^‡^*P* = 0.0002; ****P < 0.0001, by Mann-Whitney *U* test). (**L** and **M**) Representative H&E and HNF4α IHC images. Scale bar: 100 μm. (**N**–**Q**) Quantification of pERK (**N**), MCM2 (**O**), CCND1 (**P**), and pRB(S807) (**Q**) by IHC. ****P < 0.0001 (**O**); ^§^^§^^§^*P* = 0.0003 (**P** and **Q**), by Mann-Whitney *U* test.

DTP cells (87) represent a major barrier to durable responses in oncogene-driven cancers, including LUAD (88), where they facilitate survival under targeted therapy pressure. Recent work has shown that GI and mucinous (G/M) gene programs, including *Hnf4a* itself, are enriched in DTP states following RAS inhibition (44). We found that approximately 80% of these DTP-enriched genes were downregulated in KNH GEMM tumors, showing that HNF4α maintained transcriptional programs associated with the DTP phenotype in IMA (Figure 8A). This prompted us to test whether HNF4α directly modulates the response of IMA to KRAS^G12D^ inhibition.

Treatment of 1311G and 429A organoids with the KRAS^G12D^ inhibitor BMS-986508 (also called MRTX1133) demonstrated that *Hnf4a* deletion reduced the IC_50_ of BMS-986508 in 1311G organoids by 9-fold and in 429A organoids by 5-fold (Figure 8, B and C). BMS-986508 inhibited phosphorylated ERK (pERK) in both genotypes (KN and KNH), although the effect was slightly more pronounced in KNH organoids at 2 hours (Figure 8, D–F, and Supplemental Figure 12, A and B). Similar results were obtained using the independent KRAS^G12D^ inhibitor RMC-9805 (Figure 8G and Supplemental Figure 12, C and D). Consistent with these findings, the LUAD patient– derived organoid KOR259 (89), which has morphologic and molecular features of IMA, also demonstrated increased sensitivity to BMS-986508 upon *HNF4A* knockdown by CRISPR interference. (Figure 8H and Supplemental Figure 12, E and F). Extended pharmacologic testing revealed enhanced sensitivity of KNH organoids to the MEK inhibitor cobimetinib, whereas no differential response was observed with cisplatin, and both KN and KNH organoids were largely insensitive to the ERK inhibitor GDC-0994 (Supplemental Figure 12, G–I).

To evaluate in vivo responses, we subcutaneously implanted 1311G KN and KNH organoids into NSG mice and treated them with vehicle or BMS-986508. Because KNH tumors grew more slowly than KN tumors under untreated conditions (Figure 1E), we initiated treatment when tumor volumes were equivalent across genotypes to ensure a comparable baseline for assessing drug response (Supplemental Figure 12J). Consistent with the in vitro findings, most KNH tumors regressed following BMS-986508 treatment, whereas 5 of 7 KN tumors continued to grow, albeit more slowly than did vehicle controls (Figure 8, I and J, and Supplemental Figure 12K). Together, these data demonstrate that *Hnf4a* deletion sensitizes IMA to KRAS^G12D^ inhibition in vitro and in vivo.

To further assess therapeutic responses in an immune-competent setting, we treated KN and KNH GEMMs harboring autochthonous tumors with vehicle or 30 mg/kg BMS-986508 for 14 days (Supplemental Figure 13A). Histologic analysis revealed a significant reduction in tumor burden in GEMMs of both genotypes following KRAS^G12D^ inhibition (Figure 8, K and L). HNF4α expression remained comparable in vehicle- and drug-treated KN tumors, and residual tumors in KNH mice did not show selective enrichment of HNF4α^+^ cells (Figure 8M), indicating that treatment did not select for an HNF4α-expressing subpopulation. Consistent with effective pathway suppression, pERK levels were comparably reduced in both KN and KNH tumors after treatment (Figure 8N and Supplemental Figure 13B). Although residual tumor burden did not differ significantly between genotypes at this time point, cell-cycle behavior diverged markedly. Consistent with a targeted therapy response in a BRAF^V600E^-driven IMA GEMM (17), BMS-986508 paradoxically increased the percentage of MCM2^+^ DTP cells and did not reduce cyclin D1 or pRB(S807) levels in KN tumors (Figure 8, O–Q, and Supplemental Figure 13, C–E). Thus, despite ERK inhibition and tumor regression, DTP cells in KN tumors failed to exit the cell cycle. In contrast, DTP cells in KNH tumors showed significantly lower MCM2, cyclin D1, and pRB(S807) positivity, indicating that HNF4α loss promoted cell-cycle exit in DTPs in response to KRAS inhibition.

Together, these findings support a model in which HNF4α prevented quiescence in IMA DTP cells by maintaining cyclin D1 expression and Rb phosphorylation, despite KRAS and ERK inhibition. Notably, one BMS-986508-treated KN tumor exhibited histologic features consistent with mucinous-to-squamous transdifferentiation, aligning with emerging reports of lineage plasticity under KRAS inhibition (9, 90); however, this was an isolated observation (Supplemental Figure 13F).

### HNF4α impairs the response to KRAS inhibition by maintaining NRF2 activity in IMA.

**Figure 9:**
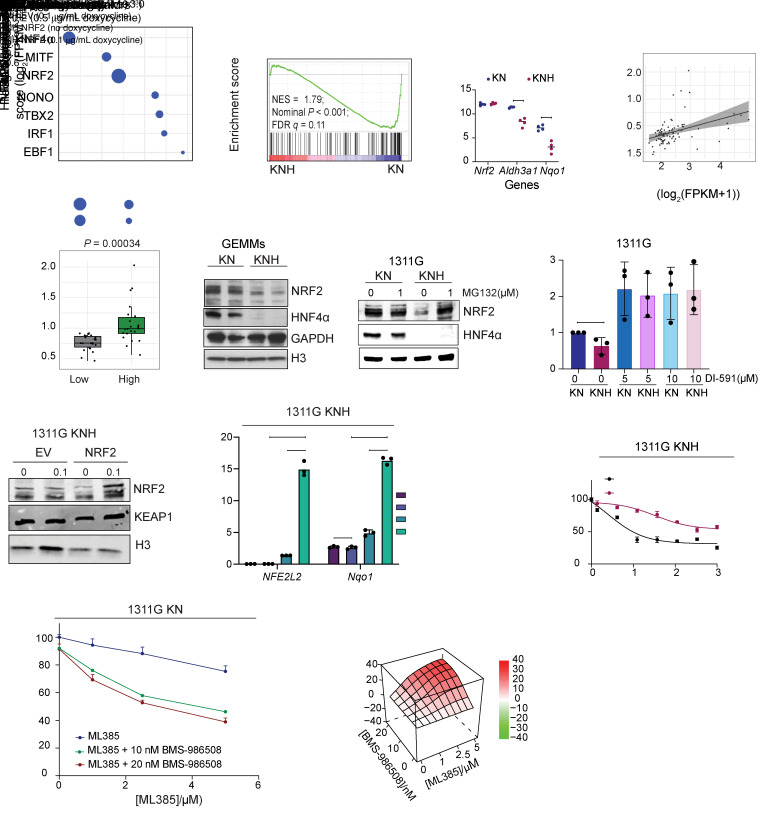
NRF2 mediates HNF4α-dependent resistance to KRAS inhibition in IMA. (**A**) IPA-predicted upstream transcriptional regulators from DEGs in KNH versus KN GEMM tumors. (**B**) GSEA showing depletion of the NRF2 gene signature in KNH GEMM tumors. (**C**) qRT-PCR of *Nrf2*, *Nqo1*, and *Aldh3a1*. ***P < 0.001, by unpaired, 2-tailed Student’s *t* test. log_2_Norm, log_2_-normalized. (**D**) Spearman correlation between NRF2 activity and HNF4A signature 1 scores in TCGA KRAS-mutant NSCLC; linear regression with 95% CI. Spearman *R* and *P* values are shown. (**E**) HNF4A signature 1 scores in NRF2-low versus NRF2-high tumors. Significance was determined by unpaired, 2-tailed Student’s *t* test. (**F**) NRF2 immunoblot of FACS-sorted IMA GEMM tumors. (**G**) Immunoblot of the indicated proteins in 1311G organoids treated with 1 μM MG132 or vehicle for 2 hours. (**H**) NRF2 quantification normalized to the loading control following 2 hours of DI-591 treatment. *n* = 3 independent biological replicates; data indicate the mean ± SD. *P* = 0.0486, by unpaired, 2-tailed Student’s *t* test. (**I** and **J**) 1311G KNH organoids expressing dox-inducible FLAG-NFE2L2 (NRF2) or empty vector (EV) ± 0.1 μg/mL doxycycline: (**I**) immunoblot of the indicated proteins (72 hours). (**J**) qRT-PCR of *NFE2L2* and *Nqo1* expression (48 hours). Data represent 1 of 3 independent biological replicates and are shown as the mean ± SD. ****P < 0.0001; NS = 0.5102, by 2-way ANOVA with Tukey’s multiple-comparison test. (**K**) Dose-response curves for 1311G KNH organoids expressing EV or dox-inducible NRF2 treated with BMS-986508 with or without 0.5 μg/mL dox for 72 hours (IC_50_: EV = 2.5 nM; NRF2 = 33.7 nM). Data represent 1 of 3 biological replicates and are shown as the mean ± SEM. (**L**) Cell viability of 1311G KN organoids treated with increasing concentrations of ML385 alone or with 10 or 20 nM BMS-986508. Data indicate the mean ± SEM from 1 of 3 biological replicates. **P < 0.01 and ***P < 0.001, by 2-way ANOVA with Tukey’s multiple-comparison test. (**M**) Bliss synergy score calculated using SynergyFinder. FPKM, fragments per kilobase of transcript per million mapped reads.

Given that BMS-986508 suppressed pERK in both genotypes, we sought to identify alternative mechanisms underlying differential sensitivity to KRAS inhibition. Pathway analysis revealed that *Hnf4a* deletion led to a marked reduction in xenobiotic metabolism, with significant suppression of the NRF2 activity in vivo, as identified by IPA upstream regulator analysis and GSEA (Figure 9, A and B, and Supplemental Figure 4, E and F). This was accompanied by reduced transcript levels of canonical NRF2 target genes, such as *Nqo1* and *Aldh3a1*, without a change in *Nfe2l2* mRNA expression, the gene encoding NRF2 (Figure 9C).

NRF2 transcriptional activity was recently associated with a poor response to KRAS inhibition in the KRYSTAL-1 trial, even in tumors lacking *KEAP1* mutations (37). We therefore asked whether HNF4A activity positively correlates with NFE2L2 (NRF2) activity in KRAS-mutant, WT *KEAP1* tumors. We obtained gene expression profiles from 88 LUAD samples harboring *KRAS* mutations but lacking *KEAP1* mutations based on data generated by The Cancer Genome Atlas (TCGA) Research Network, reasoning that these tumors maintain physiologic control of NRF2. Using this dataset, we analyzed gene expression profiles, stratified by an established NRF2 signature (37, 91) (Supplemental Table 13). Across all tumors, HNF4A Signature 1 (*R* = 0.49, *P* = 1.8 × 10^–6^), Signature 2 (*R* = 0.48, *P* = 4.1 × 10^–6^), and Signature 3 (*R* = 0.29, *P* = 0.006) showed moderate positive correlations with NRF2 activity (Figure 9D and Supplemental Figure 14A). Consistently, expression of all 3 signatures was significantly elevated in *NRF2-*high compared with *NRF2-*low tumors (*P* < 0.001 for all; Figure 9E and Supplemental Figure 14B). These results confirm that HNF4A transcriptional programs co-occurred with high NRF2 activity even in the absence of *KEAP1* mutations. In the KRYSTAL-1 cohort, in which some tumors carried *KEAP1* mutations and thus had constitutively active NRF2, we again observed moderate, significant positive correlations across all three HNF4A signatures and higher signature expression in NRF2-high cases (Supplemental Figure 14, C–F). Together, these findings support a conserved, clinically relevant relationship between HNF4A and NRF2 in KRAS-mutant LUAD.

To determine whether HNF4α regulates NRF2 at the protein level, we examined NRF2 protein expression following *Hnf4a* deletion. NRF2 protein was reduced in sorted KNH GEMM tumors and in 1311G KNH organoids. Proteasome inhibition restored NRF2 levels in vitro, indicating regulation at the level of protein stability (Figure 9, F and G). Consistent with enhanced KEAP1–cullin 3–dependent ubiquitination, pharmacologic inhibition of cullin 3 with DI-591 restored NRF2 protein levels in KNH organoids in a dose-dependent manner (Figure 9H and Supplemental Figure 15A). In contrast, chloroquine-mediated autophagy inhibition did not rescue NRF2 despite effective pathway blockade, arguing against autophagic degradation (Supplemental Figure 15, B and C). Although PPIA (92) and GSK3β (93) have been reported to modulate NRF2 stability, their protein levels (total and phosphorylated) were unchanged following *Hnf4a* deletion (Supplemental Figure 15D). Together, these data are consistent with the possibility that HNF4α maintains NRF2 stability primarily through KEAP1/cullin 3–dependent proteasomal regulation.

Given the central role of NRF2 in redox homeostasis (94), we next evaluated whether *Hnf4a* deletion alters ROS levels in IMA using 2 independent assays. The CellROX assay detected no significant differences between 1311G KN and KNH organoids, whereas the signal from the H2DCFDA assay was significantly lower in KNH, indicating that ROS differences were modest and assay dependent (Supplemental Figure 15, E and F). Consistent with a limited functional effect, exogenous H_2_O_2_ did not differentially affect viability, and antioxidant treatment failed to rescue the enhanced sensitivity of KNH organoids to KRAS inhibition (Supplemental Figure 15, G and H). Treatment with *N*-acetylcysteine (NAC) reduced NRF2 levels in both genotypes, supporting redox- sensitive control of NRF2 turnover (Supplemental Figure 15B). Together, these data indicate that KNH organoids did not have elevated overall ROS levels despite reduced NRF2 expression. Rather, they suggest that the lower NRF2 levels in KNH may have been associated with changes in specific ROS species, a possibility that will require further investigation using assays with greater chemical specificity.

We next asked whether the sensitivity of KNH organoids to BMS-986508 was due in part to reduced NRF2 levels. Pharmacologic activation of NRF2 with KI696 (95) stabilized NRF2 protein and increased *Nqo1* transcript levels, as confirmed by immunoblotting and quantitative reverse transcription PCR (qRT-PCR) (Supplemental Figure 15, I and J). KI696 also reduced sensitivity to BMS-986508 in 1311G KNH organoids in a dose-dependent manner (Supplemental Figure 15K). To complement this approach, we generated 1311G KNH organoids expressing a doxycycline-inducible *NFE2L2* construct. Doxycycline treatment induced robust NRF2 expression, as confirmed by qRT-PCR and immunoblotting (Figure 9, I and J). In cell viability assays, exogenous NRF2 expression conferred a dose-dependent rescue from BMS-986508 sensitivity compared with empty vector controls, increasing the IC_50_ by more than 10-fold (Figure 9K and Supplemental Figure 15, L and M). However, exogenous NRF2 expression did not rescue KRAS inhibitor–induced cell-cycle arrest (Supplemental Figure 15, N and O). Conversely, pharmacological inhibition of NRF2 with ML385 significantly sensitized 1311G KN organoids to BMS-986508 in a dose-dependent manner, reducing viability compared with either agent alone (Figure 9L). Synergy analysis (SynergyFinder) yielded Bliss and zero interaction potency (ZIP) scores of +21.87 and +22.07, respectively, supporting a synergistic interaction between ML385 and BMS-986508 in IMA (Figure 9M and Supplemental Figure 15P). Together, these data establish the HNF4α/NRF2 axis as a critical regulator of the KRAS inhibitor response in IMA and reveal a therapeutically targetable vulnerability.

## Discussion

Cancer progression is driven in part by the remarkable ability of malignant cells to rewire lineage programs in response to environmental and therapeutic pressures (2, 70). In LUAD, IMA exemplifies this phenomenon, adopting a gastric epithelial identity following loss of NKX2-1. However, the mechanisms that stabilize this alternative fate and constrain plasticity have remained incompletely defined (14, 32).

Our study identifies HNF4α as a central regulator of gastric lineage fidelity in IMA. Positioned within the transcriptional hierarchy downstream of NKX2-1 loss, HNF4α reinforces epithelial differentiation, constrains lineage plasticity, and maintains organized tissue architecture. Mechanistically, HNF4α cooperates with pioneer transcription factors such as FoxA1/2 at gastric regulatory elements, thereby stabilizing gastric programs and limiting FoxA1/2 redistribution to alternative lineage loci (13, 96). These findings parallel HNF4α’s known roles in developmental and adult tissues, where it serves as a guardian of epithelial identity (27, 52) and align with observations in other malignancies where loss of HNF4α correlates with loss of differentiation and lineage switching (97–99). In contrast to its dual regulation of gastric and pulmonary programs in a hybrid identity LUAD subtype (59), HNF4α binding in IMA is largely restricted to gastric loci, consistent with a more lineage-constrained transcriptional landscape.

Our integrative chromatin analyses provide mechanistic insight into how HNF4α enforces this lineage constraint. ATAC-seq revealed that HNF4α loss resulted in a redistribution of chromatin accessibility away from gastric regulatory regions toward nongastric transcriptional programs. FoxA binding shifted in parallel with these accessibility changes, with reduced occupancy at gastric loci and increased binding at nongastric loci, indicating that FoxA pioneer activity was redirected rather than abolished. Integration of ATAC-seq, ChIP-seq, and transcriptomics data demonstrated that this chromatin and transcription factor redistribution was coupled to coordinated transcriptional reprogramming toward alternative lineage states. Together, these findings support a sequential model in which FoxA1/2 establish permissive chromatin, HNF4α stabilizes gastric lineage-specific regulatory architecture, and sustained HNF4α activity is required to restrict FoxA1/2 engagement at nongastric regulatory regions and preserve lineage fidelity.

Recent studies in KRAS-driven pancreatic neoplasia have identified gastric neck and SPEM-like states as intermediates in tumor progression (100, 101). Although these findings suggest that gastric metaplasia–associated programs may represent a broader axis of KRAS-driven epithelial plasticity, we did not detect induction of a canonical SPEM-like signature after *Hnf4a* deletion in IMA. Instead, HNF4α loss led to loss of gastric pit identity with activation of multiple nongastric lineage programs, consistent with lineage plasticity rather than transition into a discrete injury-associated metaplastic state.

In addition to maintaining bulk epithelial programs, HNF4α preserves the transcriptional identity of a rare tuft-like cell population that combines gastric features with canonical neuronal and immune-associated gene signatures (102). Although canonical tuft markers remain detectable after *Hnf4a* deletion, other tuft-associated genes are reduced, suggesting that HNF4α is required to maintain the full transcriptional spectrum of tuft cells (71, 72, 103). Given emerging evidence linking tuft cells to tumor-immune interactions (71), the functional role of HNF4α-dependent tuft-like programs in IMA progression and immune modulation warrants further study.

Although our data establish HNF4α as a therapeutic vulnerability in IMA, its loss disrupts lineage constraints and permits FoxA1/2-mediated activation of alternative transcriptional programs, potentially enabling lineage switching under therapeutic pressure (49). Given the established role of FoxA1/2 in driving neuroendocrine transformation in prostate cancer (104), HNF4α-deficient IMA tumors may similarly escape KRAS blockade through transcriptional reprogramming, raising concerns about histologic evolution and acquired resistance. Thus, although HNF4α inhibition may enhance initial responses to KRAS inhibition, it may also promote relapse through activation of plasticity programs (Supplemental Figure 16), suggesting that downstream HNF4α effectors may represent more suitable therapeutic targets. Consistent with this, intact HNF4α prevents cell-cycle exit during in vivo KRAS^G12D^ inhibition by maintaining cyclin D1 levels and pRB phosphorylation in IMA (105).

NRF2 is a critical mediator of oxidative stress responses and therapeutic resistance in LUAD, and its activation is mostly attributed to mutations in KEAP1 or NRF2 itself (106–108). However, NRF2 pathway activation is not strictly restricted to *KEAP1*-mutant tumors, as elevated NRF2 transcriptional signatures have been observed in KRAS-mutant LUAD retaining WT *KEAP1*, suggesting the existence of alternative regulatory mechanisms capable of sustaining NRF2 stability and activity (107, 109). Our findings identify HNF4α as, to our knowledge, a previously unrecognized lineage-specific regulator of NRF2 stability and activity, as NRF2 and HNF4A gene signature scores were strongly correlated in human KRAS-mutant LUAD, and *Hnf4a* deletion was sufficient to reduce NRF2 protein levels in WT *KEAP1* models. These results suggest that lineage-defining transcription factors can sustain NRF2 activity independently of canonical *KEAP1* mutations, providing a mechanistic explanation for NRF2 activation in WT *KEAP1* tumors. Thus, this study identifies downstream effectors, including cyclin D1/CDK4–6 and NRF2, as candidate therapeutic vulnerabilities that may enable more selective targeting of DTP cells in IMA (87). Future studies should assess whether cotargeting cell-cycle and redox pathways can enhance the durability of KRAS-directed therapies while minimizing lineage plasticity and adaptive resistance.

Together, our findings position HNF4α as a master regulator of lineage fidelity, chromatin accessibility, and therapeutic response in IMA. By integrating developmental, transcriptional, and therapeutic axes, this study provides a conceptual framework for targeting lineage-defining transcription factors in cancer. Strategies that disrupt survival pathways while safeguarding differentiation states may be critical to achieving durable responses in lineage-plastic malignancies such as IMA and other cancer subtypes.

## Methods

### Sex as a biological variable.

Mice of both sexes were used in this study, although sex was not formally evaluated as a biological variable. Male and female mice were included in most experiments. For scRNA-seq analyses, only female mice were used to reduce potential sex-related variability, given the limited sample size (*n* = 2 per genotype). Although sex was not analyzed as an independent variable, the findings are expected to be broadly relevant across both sexes in this model.

### Statistics.

Data are presented as the mean ± SD or the mean ± SEM. For dose-response and viability assays, error bars represent the SEM of technical replicates within a representative experiment, unless otherwise specified. Graphing and statistical analyses were performed using GraphPad Prism (GraphPad Software) or R. Comparisons between 2 groups were performed using an unpaired, 2-tailed Student’s *t* test or a nonparametric Mann-Whitney *U* test, as appropriate, whereas paired comparisons were performed using a 2-tailed Wilcoxon matched-pairs, signed-rank test. Comparisons involving multiple groups were analyzed by 1-way or 2-way ANOVA with Tukey’s multiple-comparison test. Statistical details for each experiments are provided in the corresponding figure legends. For next-generation sequencing analyses, statistical testing was performed as described in the Supplemental Methods. An adjusted *P* value of less than 0.05 was considered statistically significant.

### Study approval.

All animal studies were reviewed and approved by the IACUC of the University of Utah (Salt Lake City, Utah, USA). All procedures were conducted in accordance with the Animal Welfare Act regulations and other applicable federal guidelines and adhered to the principles outlined in the *Guide for the Care and Use of Laboratory Animals* (National Research Council, National Academies Press, 2011). The University of Utah maintains an approved Public Health Service Assurance (A-3031-01). For patient-derived organoid studies, tumor acquisition and experimental use of human specimens were reviewed and approved by the IRB of the University of Utah (protocol nos. 89989 and 10924). Written informed consent was obtained from all participants prior to sample collection.

### Data availability.

Bulk RNA-seq, scRNA-seq, ChIP-seq and ATAC-seq data have been deposited in the Gene Expression Omnibus (GEO) database (GEO GSE294573 and GSE269689). Microscopy data reported in this work will be shared by the corresponding author upon request. This study does not report original code.

Values for all data points in graphs are reported in the Supporting Data Values file.

Additional detailed methods information can be found in the Supplemental Methods.

## Author contributions

HED and ELS designed experiments. HED, YSG, HUA, SAC, and M Gumbleton performed experiments. YM provided data. HED, YSG, HUA, M Guo, BTS, and ELS analyzed data. ELS and MMK performed histopathologic review. HED and ELS wrote the manuscript. All authors discussed results, reviewed and revised the manuscript.

## Conflict of interest

SC is an employee of Recursion. Matthew Gumbleton reports patents licensed to Alterna Therapeutics (US patent number US10702538B2, International patent number WO2015195812 and consulting fees or honoraria from AstraZeneca, Binaytara Foundation, Daiichi Sanyo, Dava Oncology, EMD Serono, IDEOlogy Health, Janssen Pharmaceuticals, MJH Life-Sciences, OMNI Health Media, and Total Health Oncology. ELS reports personal fees and other support from Revolution Medicines.

## Funding support

This work is the result of NIH funding, in whole or in part, and is subject to the NIH Public Access Policy. Through acceptance of this federal funding, the NIH has been given a right to make the work publicly available in PubMed Central. The content of this work is solely the responsibility of the authors and does not necessarily represent the official views of the NIH.

- NIH (R01CA212415, R01CA240317, R01CA237404, to ELS; R01CA240317, to YM; R01CA289704, to BTS).
- American Lung Association (LCD-821670, to ELS).
- Lung Cancer Research Foundation (to MG).
- National Cancer Institute (NCI), NIH (P30CA042014; core facility support).
- National Center for Research Resources, NIH (1S20RR026802-1; Flow Cytometry Core Facility).

## Supplementary Material

Supplemental data

Unedited blot and gel images

Supplemental table 1

Supplemental table 10

Supplemental table 11

Supplemental table 12

Supplemental table 13

Supplemental table 2

Supplemental table 3

Supplemental table 4

Supplemental table 5

Supplemental table 6

Supplemental table 7

Supplemental table 8

Supplemental table 9

Supporting data values

## Version 1 05/12/2026

In-Press Preview

## Version 2 07/01/2026

Electronic publication
