# Supplementary material for: HNF4α controls growth, identity, and KRAS inhibitor response in invasive mucinous adenocarcinoma of the lung
Source: J Clin Invest. 2026 May 12;136(13):e198282. doi: 10.1172/JCI198282 (PMC13318121; doi:10.1172/JCI198282)
Supplement: Supplemental data [file jci-136-198282-s176.pdf]

Supplemental Data for

HNF4α controls growth, identity, and KRAS inhibitor response in invasive mucinous adenocarcinoma of the lung

Headtlove Essel Dadzie[1,2], Yangsook Song Green[1], Soledad Camolotto[1,3], Henry U. Arnold[1,2], Matthew Gumbleton[1,4], Minzhe Guo[5], Mari Mino-Kenudson[6], Yutaka Maeda[5], Benjamin T. Spike[1,2], Eric L. Snyder[1,2,7,8*]

Contents: Supplemental Methods, Supplemental Tables and Supplemental Figures.

SUPPLEMENTAL METHODS

Experimental Models and Subject Details

Animal Studies

Mice harboring *Kras*$^{LSL\text{-}G12D}$ *(1), Kras*$^{FSF\text{-}G12D}$ (2)*, R26-*$^{FSF\text{-}CreERT2}$ (3), *Nkx2-1*^{flox} (4), *Hnf4a*^{flox} (5), *R26-CAG-LSL-Sun1-sfGFP-Myc (6)*, *p53*^{frt} *(7), NOD-Rag1-/-IL2RgammaC-null* (NRG) *(8),* and NOD*/LtSz-scid IL2R gamma null* (NSG) (9) mice have been previously described. All animals were maintained on a mixed 129/B6 background. All experimental mice were between 2 and 5 months of age at intubation.

Husbandry and housing conditions of experimental animals

Experimental mice were either bred in-house or obtained from external sources. Mice were maintained under controlled housing conditions, including regulated temperature, humidity, and light cycles, and were provided with a standardized diet. Genotyping was performed using DNA extracted from ear biopsies to confirm the presence of transgenic or knockout alleles. Mice lacking the relevant alleles were utilized as control littermates or humanely euthanized. Individual mice were identified using ear tags and assigned unique identifiers. To preserve the genetic fidelity of the models, strict handling protocols were implemented, and comprehensive records were maintained throughout the study.

Primary 3D Organoid cultures

All primary murine (1311G and 429A) and patient derived organoids including HCI_IMA03 and KOR259 organoid cultures were established within matrigel (growth factor–reduced matrigel was obtained from the Preclinical Research Shared Resource Core Facility at the University of Utah or prepared in-house) submerged in recombinant organoid medium (Advanced DMEM/F-12

supplemented with 1X B27 (Gibco), 1X N2 (Gibco), 1.25mM N-acetylcysteine (Sigma), 10mM Nicotinamide (Sigma), 10nM Gastrin (Sigma), 100ng/ml EGF (Peprotech), 100ng/ml R-spondin1 (Peprotech), 100ng/ml Noggin (Peprotech), and 100ng/ml FGF10 (Peprotech) for approximately two weeks. After establishment, organoid cultures were maintained in 50% L-WRN conditioned media (10) except KOR259, which were cultured in L-WRN medium supplemented as previously described (11). Organoid lines were tested periodically for mycoplasma contamination. To maintain organoid cultures mycoplasma free, all culture media were supplemented with 2.5 ug/ml Plasmocin. HEK293T cells were cultured in DMEM/10% FBS (Gibco).

METHOD DETAILS

Tumor initiation and tamoxifen administration in vivo

Autochthonous lung tumors were initiated by intratracheal intubation with adenoviral mSPC-FlpO virus (VVC-Snyder-6695). This virus was used for all tumor initiation experiments in the study and was obtained from the University of Iowa Viral Vector Core. Unless otherwise indicated, mice received 5×10^8 PFU per mouse. For tumor burden quantification experiments, mice received 1×10^8 PFU per mouse.

Tumor-specific activation of Cre^{ERT2} nuclear activity was achieved by intraperitoneal injection of tamoxifen (Sigma) dissolved in corn oil at a dose of 120mg/kg. Mice received 4 intraperitoneal injections over 5 days starting exactly 6 weeks after tumor initiation. Tamoxifen exposure was extended by providing tamoxifen-containing chow (Envigo) for an additional seven days. BrdU incorporation was performed by injecting mice at 40mg/kg (Sigma) intraperitoneally 1 hour prior to tissue collection.

Histology and immunohistochemistry

All tissues were fixed in 10% formalin overnight and when necessary, lungs were perfused with formalin via the trachea. Organoids were first fixed in 10% formalin overnight and then mounted in HistoGel (Thermo Fisher Scientific). Mounted organoids and tissues were transferred to 70% ethanol, embedded in paraffin, and four-micrometer sections were cut. Immunohistochemistry (IHC) was performed manually on Sequenza slide staining racks (Thermo Fisher Scientific). Sections were treated with Bloxall (Vector Labs) followed by Horse serum (Vector Labs) or Rodent Block M (Biocare Medical), primary antibody, and HRP-polymer-conjugated secondary antibody (anti-Rabbit, Goat and Rat from Vector Labs; anti-Mouse from Biocare). Slides were stained with antibodies against NKX2-1 (1:2000; Abcam; clone EP1584Y; ab76013), Cleaved Caspase-3 (CC3; 1:400; Cell Signaling Technology; clone 5A1E; #9664), CCND1 (1:200; Abcam; clone SP4; ab16663), DCAMKL1/DCLK1 (1:100; Abcam; clone EPR6085; ab109029), GFP (1:200; Cell Signaling Technology; clone D5.1; #2956), FoxA1 (1:4000; Abcam; clone EPR10881-14; ab173287), FoxA2 (1:1200; Abcam; clone EPR4466; ab108422), HNF4α (1:500; Cell Signaling Technology; clone C11F12; #3113), BrdU (1:400; Abcam; clone BU1/75 [ICR1]; ab6326), Galectin-4 (1:200; R&D Systems; AF2128), p63 (1:100; Cell Signaling Technology; clone D2K8X; #13109), ΔNp63 (1:100, Biocare; clone BC28], Cytokeratin 5 (1:200; Abcam; clone EP1601Y; ab52635), Gastrokine 1 (1:50; Abnova; clone 2E5), POU2F3 (1:200; Sigma-Aldrich; HPA019652), phospho-ERK1/2 (1:500; Cell Signaling Technology; clone D13.14.4E; #4370), TFF1 (1:100; Origene; TA382458), Pepsinogen C (1:100; Sigma-Aldrich; HPA031718), MCM2 (1:4000; Abcam; clone EPR4120; ab108935), phospho-Rb (Ser807/811) (1:1000; Cell Signaling Technology; clone D20B12; #8516), CDX2 (1:500; Cell Signaling Technology; clone D11D10; #12306), and PDX1 (1:1000; Abcam; clone EPR22002; ab219207). Images were taken on a Nikon Eclipse Ni-U microscope with a DS-Ri2 camera and NIS-Elements software. Using NIS-Elements software, tumor burden, BrdU quantification, and histologic analyses were performed on H&E- and IHC-stained slides. For tumor burden and all protein quantification analyses, measurements from all tumors within a given mouse were averaged, and the mean value per

mouse was plotted as a single data point. All histopathologic analysis was performed by a board-certified anatomic pathologist (E.L.S.).

Establishing Primary Murine and Human LUAD organoids

429A – 10 weeks after tumor initiation in KNH (*Kras*$^{FSF\text{-}G12D/+}$; *Rosa26*$^{FSF\text{-}CreERT2}$; *Nkx2-1*$^{F/F}$; *Hnf4a*$^{F/F}$) GEMM, tumor bearing mice were euthanized and lungs were isolated. Whole lung containing large numbers of microscopic tumors were minced under sterile conditions and digested at 37°C for 30 min with continuous agitation in a solution of Advanced DMEM/F12 containing the following enzymes: Collagenase Type I (Thermo Fisher Scientific, 450U/ml), Dispase (Corning, 5U/ml) and DNase I (Sigma, 0.25mg/ml). Enzymatic reactions were stopped by addition of cold Advanced DMEM/F-12 with 10% FBS. The digested tissue was repeatedly passed through a 20-gauge syringe needle, sequentially dispersed through 100 μm, 70 μm, and 40 μm cell strainers, and treated with erythrocyte lysis buffer (eBioscience) to remove residual red blood cells, yielding a single-cell suspension.

1311G – We established the organoid line from a harvested lung tumor from KNH (*Kras*$^{LSL\text{-}G12D/+}$; *Nkx2-1*$^{F/F}$; *Hnf4a*$^{F/F}$) GEMM using a lox-stop Cre viral delivery system. However, due to the poor recombination efficiency of viral Cre, we selectively harvested tumors that were NKX2-1 negative but HNF4α positive**,** as described above. By delivering Cre virus in vitro**,** we generated isogenic organoid lines that were either HNF4α-positive (KN) or HNF4α-negative (KNH)**.**

Organoid cultures were established by seeding 1×10^5 tumor cells in 50 μl of matrigel and plated in 24-well plates. Matrigel droplets were overlaid with recombinant organoid medium as described above (see "Primary 3D Organoid cultures"). Cultures were switched to 50% L-WRN conditioned media two weeks after organoid establishment. Organoid cultures were screened via immunohistochemistry and qPCR, and clones that expressed both HNF4α and NKX2-1 were selected for subsequent analysis (12).

The full names of the mouse derived 3D organoid lines mentioned in this paper are as follows: 1311G refers to "Mouse SC 1311, Tumor G, Subclone 5," and 429A denotes "Mouse HED 429 Tumor, Subclone A." These names have been abbreviated for clarity and brevity.

HCI_IMA03 – This patient-derived organoid line was established from a surgically resected primary invasive mucinous adenocarcinoma (IMA) obtained from a patient at Huntsman Cancer Institute under institutional review board (IRB)-approved protocols. Tumor tissue was minced under sterile conditions and digested at 37°C for 30 minutes with continuous agitation in Advanced DMEM/F12 containing Collagenase Type I (Thermo Fisher Scientific, 450 U/ml), Dispase (Corning, 5 U/ml), and DNase I (Sigma, 0.25 mg/ml). Enzymatic reactions were stopped with cold Advanced DMEM/F12 supplemented with 10% FBS. Tissue was further dissociated by repeated passage through a 20-gauge syringe needle, filtered through 100 µm, 70 µm, and 40 µm cell strainers, and erythrocyte lysis buffer (eBioscience) was added to lyse red blood cells. The resulting single-cell suspension was embedded in 50 µl matrigel and seeded into 24-well plates. After matrigel polymerization, wells were overlaid with recombinant organoid medium as described above (see "Primary 3D Organoid cultures"). Cultures were maintained in 50% L-WRN conditioned media following initial outgrowth. Organoids were screened via histology and qPCR for expression of HNF4α and NKX2-1, and positive clones were expanded for downstream analysis.

KOR259 – KOR259 is a patient-derived organoid line originally established by the group in Japan that first reported it and was cultured and maintained in our laboratory using previously published media conditions (11).

In vitro 4-hydroxytamoxifen (4OHT) treatment

429A organoid was transiently treated with 2mM 4OHT (Cayman Chemical Company, dissolved in 100% ethanol) or vehicle (100% ethanol) for 48hr to activate Cre^{ERT2} nuclear activity and generate isogenic pairs that are either positive or negative for HNF4α.

Generating a single cell suspension from organoid cultures

Matrigel droplets containing organoids were broken down via repeated pipetting in cell recovery solution (Corning, 500 µl per matrigel dome). Cell recovery solution containing organoids was transferred to sterile conical tubes and submerged in ice for 20-30 min before centrifugation at 4°C (300-500g). Supernatant was removed, and the cell pellet was washed in 1X PBS. Cells were then resuspended in pre-warmed TrypLE Express Enzyme (Thermo Fisher Scientific) and incubated for 10 min at 37°C. TrypLE reaction was quenched via dilution with cold Splitting Media (Advanced DMEM/F-12 [Gibco], 10 mM HEPES [Invitrogen], 1X Penicillin-Streptomycin-Glutamine [Invitrogen]). Cells were centrifuged and then resuspended in a pre-warmed DNase solution (L-WRN media supplemented to a final concentration of 200U/ml DNase [Worthington], 2.5 mM $MgCl_2$, 500 mM $CaCl_2$) and incubated for 10 min at 37°C. Cells were centrifuged and washed in PBS before use.

Immunoblotting

Organoids were treated with the indicated compounds prior to protein extraction. For proteasome inhibition studies, organoids were treated with MG132 (MedChemExpress, Cat. no. HY-13259) for the indicated duration. For Cullin neddylation inhibition, organoids were treated with DI-591 (MedChemExpress, Cat. no. HY-124602) as specified. For autophagy-lysosome inhibition, organoids were treated with chloroquine phosphate (Sigma-Aldrich, Cat. no. PHR1258) as specified. N-acetyl-L-cysteine (NAC; Sigma-Aldrich, Cat. no. A9165) was used where indicated.

Protein was extracted by lysing cells on ice for 20 mins in RIPA buffer (50 mM Tris HCl pH 7.4, 150 mM NaCl, 0.1% (w/v) sodium dodecyl sulfate, 0.5% (w/v) sodium deoxycholate, 1% (v/v) Triton X-100) plus Pierce Protease Inhibitor (PPI, Thermo Fisher Scientific, A32959). Cellular debris was pelleted for 15 min at 4°C and protein concentration was quantitated with the Pierce Bradford Protein Assay Kit (Thermo Fisher Scientific, 23200). A total of 20 µg of protein lysates were separated on Tris-Glycine precast gels (SMOBIO, QP4510) and transferred to nitrocellulose membrane (Thermo Fisher Scientific, 88018).

Antibodies used for immunoblotting included β-tubulin (1:1000; Developmental Studies Hybridoma Bank; clone E7), FoxA1 (1:1000; Abcam; ab23738), FoxA2 (1:1000; Abcam; ab108422), NKX2-1 (1:2000; Abcam; ab133638), HNF4α (1:1000; Cell Signaling Technology; clone C11F12; #3113, or 1:1000; Perseus Proteomics; clone H1415; PP-H1415-0C), phospho-ERK1/2 (1:1000; Cell Signaling Technology; #4370), total ERK1/2 (1:2000; Cell Signaling Technology; #4695 or #9107), total S6 ribosomal protein (1:1000; Cell Signaling Technology; #2317), phospho-S6 ribosomal protein (1:1000; Cell Signaling Technology; #2211), KEAP1 (1:1000; Cell Signaling Technology; #8047), GAPDH (1:1000; Cell Signaling Technology; #5174), NRF2 (1:1000; Cell Signaling Technology; clone D1Z9C; #12721), LC3A/B (1:1000; Cell Signaling Technology; clone D3U4C; #12741), p62/SQSTM1 (1:1000; Abcam; ab56416), phospho-GSK3β (Ser9) (1:1000; Cell Signaling Technology; #5558), total GSK3β (1:1000; Cell Signaling Technology; #9832), Cyclophilin A (PPIA) (1:1000; Cell Signaling Technology; clone D2Y4M; #51418), Histone H3 (1:1000; Cell Signaling Technology; #4499), and Vinculin (1:20000; Abcam; ab129002). After incubation with primary antibodies, membranes were incubated with either IRDye 800CW Goat anti-Rabbit IgG or IRDye 680RD Goat anti-Mouse IgG secondary antibodies (1:15,000 dilution; LI-COR Biosciences) and imaged using a LI-COR Odyssey CLx scanner and Image Studio software. Alternatively, HRP-conjugated secondary antibodies (1:5000; Cell Signaling Technology) and SuperSignal West Femto Maximum Sensitivity Substrate

(Thermo Fisher Scientific) were used and chemiluminescence signals were detected on autoradiography film (Santa Cruz Biotechnology, sc-201697). Densitometric quantification was performed using Image Studio or Image Lab software as appropriate.

Subcutaneous organoid allografts

KN and KNH organoids were collected and mixed with matrigel (1:1) by volume for subcutaneous allograft experiments. Organoids were subcutaneously injected into the flanks of NSG or NRG mice (~0.4 million cells per flank for 1311G and 1 million cells per flank for 429A). Tumors were allowed to grow, and their dimensions were measured using calipers. Tumor volume was calculated using the formula (L x W^2)/2 where (L) is the tumor's length and (W) is its width. Tumor volumes were monitored weekly, with measurements taken every other day. Mice were euthanized when any tumor within the cohort exceeded a volume of 1000 mm^3.

Lentiviral production and transduction

Lentivirus was produced by transfection of HEK293T cells with TransIT-293 (Mirus Bio). Packaging vectors Δ8.9 (gag/pol) and VSV-G were used for lentiviral production. Supernatant was collected at 48- and 72-hours post-transfection, centrifuged, and filtered using 0.45 μm filter units before storing long term at -80°C.

To achieve stable transduction of organoids, cultures were dissociated into single-cell suspensions as previously described (see "Generating a Single-cell Suspension from Organoid Cultures"). The cells were resuspended in a 1:1 mixture of 50% L-WRN and thawed lentivirus-containing supernatant. Polybrene (MedChemExpress, HY-112735) was added to a final concentration of 8 μg/mL, and the cells were incubated for 24 hours with gentle agitation. Following incubation, the cells were pelleted, embedded in matrigel, and seeded. After 72 hours,

antibiotic selection (puromycin = 5 μg/mL or blasticidin = 10 μg/mL) was done for at least one week to establish stable lines for downstream assays.

Additionally, CRISPR interference (CRISPRi) targeting *HNF4A* in KOR259 organoids was performed using previously validated dual sgRNAs against the P1 and P2 *HNF4A* isoforms, as described previously (13).

BMS-986508 treatment in autochthonous and allografts mouse models

BMS-986508 was provided by BMS and formulated at 10% (w/v) Captisol in 50 mM citrate buffer, pH 5.0, by mixing Captisol and citrate buffer, adjusting pH with NaOH, and dissolving drug by vortexing and heating to 37°C prior to use. For allografts studies, NSG mice were subcutaneously implanted with either 1311G KN or KNH organoids suspended in matrigel. Tumor-bearing mice were randomized once tumors reached ~150 mm^3. BMS-986508 or vehicle control (10% Captisol in 50 mM citrate buffer) was administered by daily intraperitoneal (IP) injection at a dose of 10 mg/kg for 7 days, followed by dose escalation to 30 mg/kg for an additional 14 days.

For autochthonous tumor models, tumors were initiated in KN and KNH GEMMs as described above (see "Tumor initiation and tamoxifen administration in vivo"). BMS-986508 was delivered by daily IP injection at 30 mg/kg in 10% Captisol in 50 mM citrate buffer (pH 5.0), beginning at 12 weeks post-tumor initiation, for a total duration of 14 consecutive days prior to sacrifice.

Drug treatments and PrestoBlue organoid viability assays

Cisplatin (MedChemExpress, Cat. no. HY-17394), RMC-9805 (MedChemExpress, Cat. no. HY-156819), ML385 (MedChemExpress, Cat. no. HY-100523), and KI696 (MedChemExpress, Cat. no. HY-101140) were purchased from MedChemExpress. Hydrogen peroxide (H_2O_2; Thermo Fisher Scientific, Cat. no. 033323-AP; 30% [8.80 M] in H_2O) was purchased from Thermo Fisher Scientific. The ERK inhibitor GDC-0994 and cobimetinib (GDC-0973) were obtained from

Genentech. Doxycycline (Sigma-Aldrich, D9891) was used where indicated. Cisplatin was dissolved in sterile H_2O, and RMC-9805, ML385, KI696, GDC-0994, and cobimetinib were dissolved in DMSO according to manufacturer recommendations.

Organoids were dissociated into single-cell suspensions as described above and seeded at equal density in 96-well, clear-bottom plates in 5 μL of Matrigel per well with 100 μL of L-WRN medium per well. For indicated experiments, 429A organoids were pretreated with 4-OHT or ethanol vehicle control for 48 hours before seeding, whereas chronically deleted *Hnf4a* 1311G organoids were used as indicated. For KOR259 experiments, organoids were seeded after lentiviral transduction with empty vector or dual sgRNAs targeting the P1 and P2 *HNF4A* isoforms, followed by 4 days of antibiotic selection.

Baseline viability was measured by adding 10 μL of PrestoBlue HS Cell Viability Reagent (Invitrogen) to each well and incubating at 37°C for 30 minutes. Fluorescence was measured using a Synergy HTX plate reader (excitation, 528/20 nm; emission, 590/20 nm). The reagent was then removed, wells were washed with warm PBS, and fresh L-WRN medium containing the indicated drugs or corresponding vehicle controls was added. For doxycycline-inducible experiments, doxycycline-containing media were replenished every 24 hours. Cell viability was assessed using the PrestoBlue assay after 72 hours of treatment unless otherwise specified. Fluorescence values were normalized to vehicle-treated controls, and dose-response curves were generated using nonlinear regression in GraphPad Prism. Data are presented as mean ± SEM of technical replicates unless otherwise stated.

Edu incorporation and cell cycle analysis

Cell proliferation and cell cycle distribution were assessed using the Click-iT EdU assay kit (Thermo Fisher Scientific) according to the manufacturer's instructions, with modifications for organoid culture. Briefly, organoids were treated with the indicated drugs for 48 hours, and EdU

was added directly to the existing culture medium during the final 4 hours of treatment to a final concentration of 0.01 mM. The domes were then washed twice with PBS, and organoids were dissociated into single cells using the organoid dissociation protocol described above. Cells were washed in PBS containing 1% BSA, fixed in 4% formaldehyde for 15 minutes, and washed twice more in PBS containing 1% BSA. EdU detection was performed the following day. Immediately before flow cytometric analysis, cells were permeabilized in 1X saponin (Invitrogen) in PBS containing 1% BSA, and 500 µL of Click-iT reaction cocktail containing CuSO4, ascorbic acid, and Alexa Fluor 642 azide (Invitrogen) was added to each sample. Cells were incubated for 30 minutes protected from light, washed in 1X saponin in PBS containing 1% BSA, and analyzed by flow cytometry on a BD Fortessa. DAPI (Thermo Fisher Scientific, Cat. no. D1306) was used to measure DNA content. EdU incorporation was used to quantify S-phase cells, and DAPI intensity was used to determine cell cycle phase distribution. FlowJo software was used to analyze the acquired data.

Intracellular ROS measurement

Organoids were cultured in LWRN medium. 30 mins prior to cell collection, culture medium was replaced with prewarmed PBS containing 5 µM H_2DCFDA (MedChemExpress, HY-D0940; excitation/emission 495/529 nm) or with LWRN medium containing 5 µM CellROX Deep Red reagent (Thermo Fisher Scientific, C10422; excitation/emission 644/665 nm). Organoids were incubated for 30 minutes at 37°C in the dark. Following incubation, staining solutions were removed, and organoids were washed twice with pre-warmed PBS before dissociation into single cells using cell recovery solution, as described above. Single-cell suspensions were passed through a 35 µm cell strainer, resuspended in buffer containing DAPI, and analyzed on a BD LSRFortessa flow cytometer to quantify ROS-positive cells within the live (DAPI-negative) population. FlowJo software was used to analyze the acquired data.

RNA extraction, cDNA synthesis, and qPCR

RNA was isolated via Trizol-chloroform extraction followed by column-based purification. The aqueous phase was brought to a final concentration of 35% ethanol, and RNA was purified using the PureLink RNA Mini Kit (Thermo Fisher Scientific) according to the manufacturer's specifications.

cDNA was synthesized from Trizol-extracted RNA using LunaScript RT SuperMix (NEB, M3010) according to manufacturer's specifications. qPCR was performed on cDNA using Luna Universal Probe qPCR Master Mix (NEB, M3004) according to manufacturer's specifications, and 35 cycles were used for the denaturation and extension steps. Transcript levels were normalized to *PPIA* and quantitated by the $\Delta\Delta$Ct method.

Bulk RNA sequencing (RNA-seq)

In vivo bulk RNA-seq

14 weeks after tumor initiation, KN and KNH mice were euthanized, and the ribcage was dissected to reveal the trachea and heart. Cardiac perfusion of the pulmonary vasculature was performed using PBS until the lungs turned pale. Lungs were then removed, digested, and filtered as previously described (see "Establishing primary murine and human LUAD organoids") to obtain a single cell suspension. Samples were resuspended in FACS buffer with DAPI. Cells were sorted on the BD FACSAria with the 85 μm nozzle to obtain a GFP-positive, DAPI-negative population. Samples were sorted into 1 ml of cold PBS with 10% serum. After sorting, cells were centrifuged for 10 min at 4 ^{0}C (300g) and resuspended in 1 ml of Trizol. RNA was isolated as previously described (see "RNA extraction, cDNA synthesis, and qPCR"). Library preparation was performed using the NEBNext Ultra II Directional RNA Library Prep with rRNA Depletion Kit for mouse.

Sequencing was performed using the Illumina NovaSeq 6000 (150 x 150 bp paired-end sequencing, 25 million reads per sample).

In vitro bulk RNA-seq

RNA was collected from 2 biological replicates of the following conditions: 429A KN and KNH isogenic organoid cultures (2 weeks following 4OHT or ethanol treatment) and 1311G KN and KNH isogenic organoids (> 4 weeks following Ad5CMV-Cre treatment). Organoids were collected directly into Trizol and stored at -80°C until purification. For organoids, 4 confluent 20 μL matrigel domes were collected per sample.

RNA was isolated as previously described (see "RNA extraction, cDNA synthesis, and qPCR"). Library preparation was performed using the NEBNext Ultra II Directional RNA Library Prep with poly(A) mRNA isolation. Sequencing was performed using the Illumina NovaSeq 6000 (150 x 150 bp paired-end sequencing, 25 million reads per sample).

Data Processing and Analysis (bulk RNA-seq)

The mouse mm10 and gene feature files were downloaded from Ensembl and a reference database was created using STAR version 2.7.6a (14). Optical duplicates were removed from NovaSeq runs via Clumpify v38.34 (15). Reads were trimmed of adapters and aligned to the reference database using STAR in two-pass mode to output a BAM file sorted by coordinates. Mapped reads were assigned to annotated genes using feature Counts version 1.6.3 (16). Raw counts were filtered to remove features with zero counts and features with five or fewer reads in every sample. Differential expressed genes (DEGs) were identified using the hciR package (https://github.com/HuntsmanCancerInstitute/hciR) with a 5% false discovery rate and DESeq2 version 1.34.0 (17). GSEA-Preranked was run with the differential gene list generated from

DESeq2 and the following gene sets: C2, C5, C8 and Hallmarks gene sets from MsigDB (18, 19). Gene sets smaller than 15 and larger than 500 were excluded from analysis.

Single-cell RNA Sequencing (scRNA-seq)

Sample Preparation

Both KN (n=2) and KNH (n=2) mice were administered tamoxifen at 6 weeks post-intubation for a duration of 2 weeks (one week of IP tamoxifen at 120mg/kg followed by one week on tamoxifen chow). 6 weeks later, single-cell suspensions were prepared as follows: lungs and heart were perfused with PBS, and whole lung filled with microscopic tumors were dissected and dissociated into single cells as outlined in the "Establishing Primary Murine and human LUAD Organoids" protocol. Samples were resuspended in PBS +1% BSA buffer with DAPI. Cells were sorted on the BD FACSAria cell sorter with the 85 μm nozzle to obtain a GFP-positive single cells (DAPI negative population). After sorting, cells were centrifuged for 10 min at 4^0C (300g) and resuspended in PBS with 1% BSA for library preparation on the same day.

Library Preparation and Sequencing

The scRNA-seq libraries were generated using the 10x Genomics Chromium Single Cell Gene Expression Solution with 3’ chemistry (version 3, PN-1000075). This process was conducted at the High-Throughput Genomics Shared Resource, Huntsman Cancer Institute, University of Utah. Single-cell suspensions, filtered through a 40 μm strainer, were assessed for viability and cell count using Countess II (Thermo Scientific) and adjusted to a target recovery of 10,000 cells. The suspension was loaded into Chromium Single Cell A Chip (PN-120236), where Gel Beads-in-Emulsion (GEMs) were formed. Reverse transcription synthesized cDNA from barcoded mRNA within GEMs, followed by subsequent library preparation steps, including A-tailing, end repair, adaptor ligation, and indexing. Library quality was assessed with Agilent D1000 Screen Tape and

quantified via qPCR using KAPA Biosystems Library Quantification Kit for Illumina Platforms (KK4842). Libraries were normalized and sequenced on a NovaSeq 6000 in paired-end mode (2×150).

Data Processing and Analysis (scRNA-seq)

Demultiplexing and Alignment

Demultiplexing of scRNA-seq data from KN (n=2) and KNH (n=2) tumors was performed using Cell Ranger (mkfastq version 3.1.0) to generate fastq files. Reads were aligned to the mouse genome (mm10), supplemented with references for CRE-ERT2 and *Hnf4a* exons, using Cell Ranger count (version 3.1.0). The expected cell count was set to 10,000 per library. For KN samples, approximately 6,497 cells were captured, with an average of 36,677 reads per cell and a median of 3,329 genes per cell. For KNH samples, approximately 5,817 cells were captured, with an average of 41,861 reads per cell and a median of 3,641 genes per cell. Additional details of the primary Cell Ranger data processing can be found at: link to 10x

Quality Control and Clustering

Single cell expression data was subjected to common Seurat workflows for initial quality control and clustering (20). Specifically, Seurat (version 4) workflows were employed for QC and clustering. Cells with feature counts <500 or >7,500 and mitochondrial content >10% were excluded. Data were normalized, scaled, and dimensionally reduced using PCA (25 PCs with a resolution of 1). A KNN graph was constructed, and clusters were identified using the Louvain algorithm. UMAP embedding revealed 17 clusters which included some immune cells that were captured. High-quality tumor cells from KN and KNH were identified, sub-setted, and reclustered based on 19 PCs. Tumor cell barcodes used for downstream analyses are provided in

Supplemental Table 10. To identify KNH complete recombinants, expression of the floxed fourth and fifth exons of *Hnf4a* were quantified.

Differential Gene Expression and Scoring

DEGs in UMAP clusters were identified using Seurat's FindMarkers function. Gene signature scores for published gene signatures including various additional cell types and states (MsigDB) used in this paper were calculated using the AddModuleScore function. Published gene lists for these scores are available in referenced papers. CytoTRACE (21) scores, ranging from 0 (most differentiated) to 1 (least differentiated), were computed using the CytoTRACE R package (v1.0.0).

Data Imputation

The Adaptively thresholded Low-Rank Approximation (ALRA) method was applied to impute low-detection genes in our scRNA-seq dataset, preserving biological zeros and utilizing low-rank approximation (22).

Chromatin immunoprecipitation sequencing (ChIP-seq)

In vitro organoid ChIP-seq

For all organoid ChIP-seq experiments, organoids from two 24-well plates (approximately 4-8 million cells) were collected in cell recovery solution (500 μl per well). For *Hnf4a* deletion studies, 429A organoids were treated with 4-OHT or ethanol (vehicle control) for 48 hours to induce *Hnf4a* recombination. In parallel, 1311G organoids previously transduced in vitro with Ad5CMV-Cre to delete *Hnf4a* were used for complementary analyses. Organoids were incubated in cell recovery solution on ice for 30 minutes and then pelleted by centrifugation at 4°C and 300g. Cells were washed three times in cold PBS. On the second wash, PBS was supplemented with

DNase solution (containing a final concentration of 200U/ml DNase [Worthington], 2.5 mM MgCl2, 500 mM CaCl2). After the third wash, organoids were resuspended in 5 ml of 2 mM DSG buffer (1X PBS, 1 mM MgCl2) and rotated at room temperature for 35 min. Formaldehyde was then added to a final concentration of 1% and cells were crosslinked for 10 min. The cross-linking reaction was stopped with the addition of glycine to a final concentration of 125mM. Cells were washed with cold PBS, then frozen at -80°C. Cell pellets were thawed on ice for 5 minutes then lysed in 1 mL of Farnham lysis buffer (5 mM PIPES pH 8.0, 85 mM KCl, 0.5% NP40) with Pierce Protease Inhibitor (PPI, ThermoFisher Scientific, A32959). Samples were centrifuged at 4°C (1000g) then resuspended in 1mL of RIPA lysis buffer (1X PBS, 1% NP40, 0.5% sodium deoxycholate, 0.1% sodium dodecyl sulfate) with PPI. Chromatin was sonicated with a QSonica Q800R (pulse: 30s on / 30s off; sonication time: 20 minutes; amplitude: 70%). After sonication, samples were centrifuged at 17,000 × g for 15 minutes, and input chromatin was collected from each sample. Chromatin was then immunoprecipitated overnight with 5 µg of antibody per sample prebound to Protein G Dynabeads (for mouse antibodies; Thermo Fisher Scientific; #10004D) or Protein A Dynabeads (for rabbit antibodies; Thermo Fisher Scientific; #10002D). The following antibodies were used: HNF4α (Perseus Proteomics; clone H1415; PP-H1415-0C), FoxA1 (Abcam; ab170933), and FoxA2 (Cell Signaling Technology; #8186). Bead-bound chromatin was washed 5 times with LiCl wash buffer (100 mM Tris-HCl, pH 7.5; 500 mM LiCl; 1% NP-40; 1% sodium deoxycholate), and crosslinks were reversed by incubation in IP elution buffer (1% SDS; 0.1% $NaHCO_3$) overnight at 65°C. DNA was purified using the Zymo ChIP DNA Clean and Concentrator Kit (Zymo Research; #D5205). Library preparation was performed using the ChIP-seq with NEBNext DNA Ultra II library prep kit using Unique Molecular Indexes (UMIs). Sequencing was performed using the Illumina NovaSeq 6000 (150x150 bp paired-end sequencing, 25 million reads per sample).

In vivo nuclei ChIP-seq

14 weeks after tumor initiation, KN and KNH tumor-bearing mice were euthanized, and the ribcage was dissected to reveal the trachea and heart. Lungs were perfused with cold PBS, removed, and snap frozen in liquid nitrogen. Flash-frozen lungs were minced on ice in 2 ml of ice-cold PBS for 3 - 5 min. Minced lungs were dounced 10 times with a large 7 ml homogenizer (Wheaton, item #23ND78) and centrifuged for 5 min at 4^0C (300g). Tissue was crosslinked in 2 mM DSG buffer and 1% formaldehyde as previously described (In vitro organoid ChIP-seq). After fixation, lung samples were resuspended in 2 - 3 ml of TST buffer with protease inhibitors for 5 min. During this time, samples were dounced an additional 5 times in a small 2 ml homogenizer (Wheaton, item #23ND70) to extract nuclei. Lysis was quenched with 5 ml of 1X ST buffer plus protease inhibitors. Nuclei were washed in cold PBS and resuspended in 5 - 10 ml cold PBS supplemented with DAPI and protease inhibitors. Nuclei suspension was then sequentially filtered through a 70 μm and 35 μm cell strainer.

Before sorting, nuclei were evaluated on a fluorescence microscope to assess for GFP positivity and nuclear integrity. Nuclei were sorted using BD FACSAria with the 85 μm nozzle for GFP-positive, DAPI-positive nuclei. Samples were sorted into 1 ml of cold PBS with 1% BSA and 10X protease inhibitor. Approximately 10 million nuclei were sorted for ChIP-seq for transcription factor (TF) ChIP-seq experiments. After sorting, nuclei were pelleted for 10 min at 4^0C (500g). Samples were then resuspended in Chromatrap hypotonic and lysis buffers as previously described (see "In vitro organoid ChIP-seq") and sonicated with QSonica Q800R (pulse: 30s on /30s off; sonication time: 20 min; amplitude: 70%). Chromatin was immunoprecipitated with antibodies prebound to Protein G Dynabeads for mouse antibodies or Protein A Dynabeads for rabbit antibodies. The following antibodies were used: HNF4α (Perseus Proteomics; clone H1415; PP-H1415-0C), FoxA1 (Abcam; ab170933; rabbit monoclonal; 5 μg per ChIP), and FoxA2 (Cell Signaling Technology; clone D56D6; #8186; rabbit monoclonal; 5 μg per ChIP). ChIP-seq libraries were prepared and sequenced as previously described (see "In vitro organoid ChIP-seq").

Data Processing and Analysis(ChIP-seq)

Fastq alignments were pre-processed with the merge_umi_fastq application from the UMIScripts package (https://github.com/HuntsmanCancerInstitute/UMIScripts) to associate the UMI sequence, provided as a third Fastq file, into the read comment. Reads were aligned using Bowtie2 v2.2.9 (23) to the standard chromosomes of the mouse genome (version mm10) or the human genome (version hg38). Duplicate alignments based on the UMI code were removed using the bam_umi_dedup application (UMI Scripts) allowing for 1 mismatch. Peaks were called using MACS2 v2.2.7 (24) a significance of q-value < 0.01. Coverage tracks were generated with MACS2 as Reads Per Million. Input libraries were obtained from all organoid samples and were used as controls for each ChIP-seq experiment. All ChIP-seq experiments were performed in biological duplicates. Peaks called in both biological replicates were identified using Bed tools v2.28.0 (25) with a 1-bp minimum overlap to generate a consensus list of peaks for downstream analysis. Genomic annotation of binding sites was performed using HOMER (26). Motif analysis was performed on 100-bp regions surrounding the summit of identified peaks using the HOMER package.

Differential ChIP-seq peaks were identified using the Diffbind package v3.4.11 (27) with a q-value cutoff < 0.05 using DESeq (17). Motif analysis of differential ChIP-seq peaks was performed using HOMER, using the full 400-bp regions of differential peaks identified by DiffBind. Heat maps and profile plots were generated using deeptools v3.5.1 (28). Pathway analysis was performed on annotated differential ChIP-seq peaks using Enrichr (29).

Integration of HNF4α ChIP-seq and H3K27ac HiChIP Data

Previously published H3K27ac HiChIP data from KN GEMM tumors (30) were integrated with HNF4α ChIP-seq dataset from KN GEMM tumors to identify enhancer- and promoter-associated

chromatin interactions. High-confidence loops (FDR < 0.01, HiChIPcounts > 2) were filtered to remove duplicates and adjacent anchors corresponding to the same transcriptional unit.

For each retained loop, anchor regions were compared to HNF4α peak coordinates (from MACS2 peak calls) to identify direct overlaps. The gene-proximal anchor (within the transcribed region or promoter) was inferred using loop-associated gene annotations. The distal anchor was designated as the putative enhancer. Anchors overlapping HNF4α peaks were flagged as HNF4A-bound, and each loop was classified as promoter, enhancer, or both, depending on the location of HNF4α binding. Genes with both promoter- and enhancer-bound loops were annotated as both. Final annotations included loop coordinates, HNF4α binding status, loop strength (HiChIPcounts), FDR, and associated gene identity.

FoxA1/2 and HNF4α Motif Abundance and Strength Analysis

FOXA and HNF4 motif analyses were performed across four enhancer sets: Class I (union or intersect) and Class II (KN or KNH dynamic). Intersect represent peaks present in both genotypes and union represents the nonredundant set of all peaks detected in either KN or KNH. Enhancer sets were derived from FoxA1 or FoxA2 ChIP-seq peak calls.

Motif scanning was performed using FIMO (MEME Suite v5+) with default Markov background parameters (31) and JASPAR FoxA1 and FoxA2 motifs (MA0148.x and MA0047.x), evaluated at two significance thresholds ($P \leq 1\times10^{-4}$ and $P \leq 1\times10^{-5}$). All analyses were conducted separately for each motif-threshold combination.

Motif abundance was quantified by counting the number of enhancer sequences containing at least one FIMO hit (N_hit) and calculating the fraction of motif-positive sequences (N_hit/N_total), with remaining sequences classified as (N_nohit). Relative motif enrichment was assessed using 2×2 contingency tables across all pairwise comparisons among KN dynamic, KNH dynamic,

intersect, and union enhancer sets. Statistical significance was evaluated using Fisher's exact test, with *P*-values adjusted by the Benjamini-Hochberg method within each motif-threshold combination.

Motif strength was assessed by comparing FIMO log-odds scores using an approach informed by prior motif-strength analyses (32). All individual motif occurrences within each enhancer set were pooled, and for each pairwise comparison the number of occurrences, median log-odds scores, and differences between medians were calculated. Distributions were compared using two-sided Wilcoxon rank-sum tests, with *P*-values adjusted by the Benjamini-Hochberg method within each motif-threshold combination. For visualization, the maximum log-odds score per enhancer was also computed and compared across enhancer sets using the same statistical framework.

Similar analyses were done for the HNF4α motifs using either FoxA1 or FoxA2 ChIP-seq peaks. All statistical analyses were performed in R (version 4 or later) using fisher.test, wilcox.test, and *P*.adjust, and figures were generated using ggplot2.

ATAC-seq for Organoids: Sample processing

ATAC-seq was performed on organoids using an Omni-ATAC-based protocol adapted from published methods (33, 34). Organoids were dissociated into single cells using established protocol, depleted of dead cells by two sequential rounds of magnetic separation using Miltenyi Biotec Dead Cell Removal Kit, and washed in PBS. Cell viability exceeded 90% for all samples. Cells were manually counted, and 100,000 cells per condition were pelleted at 500g for 5 minutes at 4°C in PBS supplemented with 1% BSA. For nuclei isolation, cell pellets were resuspended in cold lysis buffer and incubated on ice for 3 minutes. After addition of wash buffer, nuclei were pelleted at 500g for 10 minutes at 4°C and gently resuspended in transposition mix lacking Tn5. Nuclei were counted, and 50,000 nuclei per reaction were used for transposition. Transposition

was performed with 2.5uL of Illumina Tn5 and incubated at 37°C for 30 minutes with shaking at 1,000 rpm. Transposed DNA was purified using Zymo DNA Clean and Concentrator and eluted in 21 µL elution buffer.

Libraries were amplified using Q5 Hot Start High-Fidelity Master Mix (NEB) and Nextera barcoded primers. Initial amplification was performed for five cycles, followed by SYBR Green–based qPCR on 10% of each library to determine the minimum number of additional cycles required. Final libraries were purified using Zymo DNA Clean and Concentrator kit. Library concentration was quantified by Qubit, and size distribution was assessed by Agilent TapeStation. Libraries were sequenced using paired-end 150 bp reads on an Illumina platform.

ATAC-seq data processing and analysis

ATAC-seq data were processed using the same computational pipeline as ChIP-seq, with modifications appropriate for chromatin accessibility profiling. Adapters were trimmed using Trim Galore v0.6.6 and quality was assessed using FastQC v0.12.1. Reads were aligned to the mouse (mm10) genome using Bowtie2 v2.2.9 (23). Aligned reads were sorted and indexed using Sam Tools v1.16 (26). Duplicates were removed using Picard v2.22.0 and peaks were called using MACS2 v2.2.7 (24). Coverage tracks were generated as reads per million. Differential accessibility analysis was performed using DiffBind v3.4.11 (27) with a false discovery rate q-value cutoff of <0.05 using DESeq2 (17) as the analysis method. Peak annotation and motif enrichment analyses were conducted using HOMER v4.11.1 (26). Heatmaps were generated using deepTools v3.5.1 (28). All experiments were performed in biological duplicates, and reproducible peaks were defined by intersecting peaks present in both replicates using Bedtools v2.28.0 with a minimum 1-bp overlap. Pathway enrichment analysis of genes associated with differentially accessible regions was carried out using Enrichr.

NRF2 and HNF4A Signature Scoring and Stratification Analysis

Gene expression data from 88 NSCLC lung adenocarcinoma (LUAD) tumors with confirmed KRAS mutations and wild-type KEAP1 status were obtained from The Cancer Genome Atlas (TCGA). Normalized transcript abundance (log_2(FPKM + 1)) was used after removing genes with duplicated names or zero expression across all samples. HNF4A and NRF2 signature scores were computed by averaging the log_2-transformed expression of genes in each signature, retaining only those present in the dataset. Signature 1 comprised the top 100 DEGs upregulated in KN GEMM tumors; Signature 2 included 100 DEGs induced upon HNF4A overexpression in H2122 cells and Signature 3 was derived from hybrid human-mouse LUAD models (35). NRF2 activity was assessed using a published gene set utilized in the KRYSTAL study (36, 37). Samples were stratified into NRF2_Low (≤Q1) and NRF2_High (≥Q3) groups based on NRF2 scores, excluding intermediate cases. Two-tailed unpaired Student's *t* tests (α = 0.05) were used to compare HNF4A scores between NRF2_low and NRF2_high tumors. Spearman correlations between NRF2 and HNF4A activity scores were computed across all 88 tumors.

For comparison, gene expression data from 68 KRAS-mutant NSCLC patients enrolled in the KRYSTAL-1 clinical trial (NCT03785249) (36) were obtained from the supplementary materials of a previously published study (38). Genes with zero counts across all samples and those with CPM<1 in >50% of samples were excluded. Remaining counts were normalized using edgeR (v3.36.0), and log_2CPM values were computed with a prior count of one. HNF4A and NRF2 signature scores were calculated as above. Tumors were stratified into NRF2_Low and NRF2_High based on quartiles, and two-tailed unpaired *t* tests were used to compare HNF4A activity scores between groups. Spearman correlation analyses were also performed across the full cohort. Boxplots and scatter plots were generated in R using ggplot2 (via ggpubr v0.4.0 and cowplot v1.1.1), with a single least-squares regression line, 95% confidence intervals, and annotated Spearman R and *P* values.

Synergy Score

ZIP and Bliss Synergy scores were calculated the using the Bioconductor SynergyFinder package (v3.14.0).

SUPPLEMENTAL TABLES

Supplemental Table 1: ChIP-seq peaks and gene annotations for GEMM (KN and KNH) tumors and organoids (1311G, 429A and HCI_ IMA03).

Supplemental Table 2: Raw counts, normalized counts and DEGs in GEMM (KN and KNH) tumors and summary DEGs from the human H2122 2D cell line.

Supplemental Table 3: Integration of HNF4α ChIP-seq with H3K27ac HiChIP in KN GEMM tumors.

Supplemental Table 4: Gene set enrichment analysis (GSEA) of C8 cell type signatures using DEGs from GEMM (KN and KNH) tumors.

Supplemental Table 5: Raw counts, normalized counts and DEGs in 429A murine organoids.

Supplemental Table 6: Raw counts, normalized counts and DEGs in 1311G murine organoids.

Supplemental Table 7: GSEA cell-type (C8) analysis of DEGs in 429A and 1311G murine organoids.

Supplemental Table 8: Halmark, KEGG and Reactome analysis of DEGs from GEMM (KN and KNH) tumors.

Supplemental Table 9: GSEA cell-type (C8) analysis of pooled DEGs from 429A and 1311G murine organoids.

Supplemental Table 10: scRNA-seq tumor cell barcodes, cluster associations, cluster-specific DEGs, HNF4α exon counts, and pathway analysis between Group A and B.

Supplemental Table 11: Differentially bound FoxA1/2 peaks and gene annotations in GEMM (KN and KNH) tumors and 1311G organoids.

Supplemental Table 12: Abundance and Motif strength analysis for FoxA1/2 and HNF4α motifs

Supplemental Table 13: Published NRF2 gene signature, HNF4A Signature 1-3 and TCGA gene expression data for KRAS mutant, KEAP1 wild-type tumors.

Supplemental Figure 1

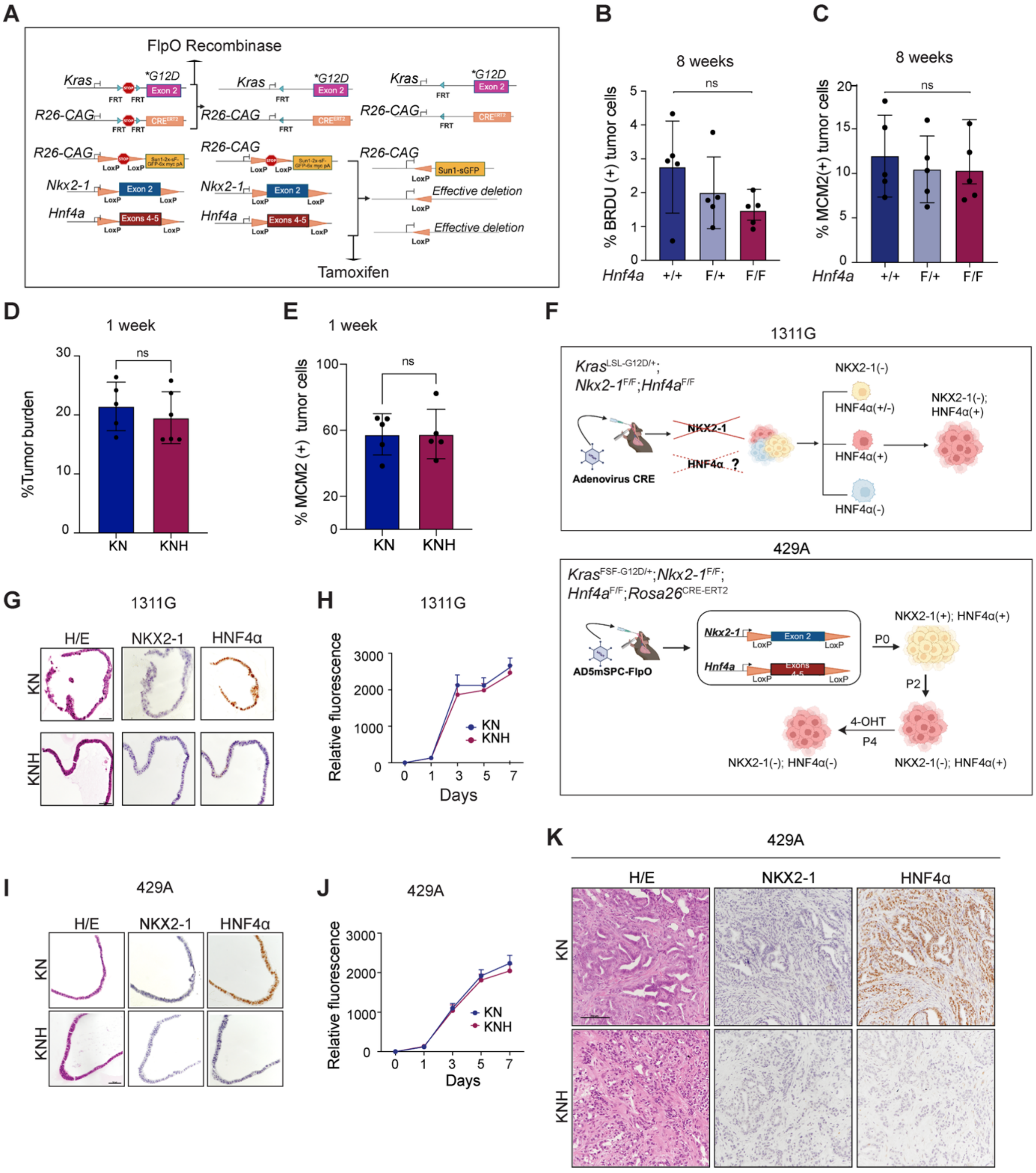

Supplemental Figure 1. Generation and characterization of GEMMS and organoid models used in this study.

(**A**) Graphical schematic showing generation of GEMMs of IMA. Created in BioRender. (**B–C**) Quantification of BrdU incorporation (B) and MCM2 expression (C) by IHC in the indicated genotypes at 14 weeks post-tumor initiation (8 weeks post first IP dose of tamoxifen). One-way ANOVA showed no significant differences in BrdU incorporation (F (2, 12) = 1.99, P = 0.1799, R^2 = 0.25) or MCM2 expression (F (2, 12) = 0.2512, P = 0.7818, R^2 = 0.04) between genotypes. (**D–E**) Mice were intubated with Ad5mSPC-FlpO virus, and lungs were harvested at 7 weeks post-tumor initiation (1 week post first IP dose of tamoxifen) in KN and KNH GEMMs for quantification of overall tumor burden (D, unpaired Student's *t* test; P = 0.48) and MCM2 expression by IHC (E, unpaired Student's *t* test; P = 0.98). (**F**) Schematic of organoid generation. Top: 1311G organoid line from a $Kras^{LSL\text{-}G12D/+}$; $Nkx2\text{-}1^{F/F}$; $Hnf4a^{F/F}$ mouse. Bottom: 429A organoid line from a $Kras^{FSF\text{-}G12D/+;}$ $Rosa26^{FSF\text{-}CreERT2}$; $Nkx2\text{-}1^{F/F}$; $Hnf4a^{F/F}$ mouse. Created in BioRender. (**G**) Representative H&E and IHC images for NKX2-1 and HNF4α in 1311G KN and KNH organoids. Scale bar: 100 µm. (**H**) PrestoBlue viability assay measuring growth of 1311G KN and KNH organoids. Data shown represent 1 representative replicate of 3 independent biological replicates. Error bars indicate SD of technical replicates. (**I**) Representative H&E and IHC images for NKX2-1 and HNF4α in 429A KN and KNH organoids. Scale bar: 100 µm. (**J**) PrestoBlue viability assay measuring growth of 429A KN and KNH organoids. Data shown represent 1 representative replicate of 3 independent biological replicates. Error bars indicate SD of technical replicates. (**K**) Representative H&E and IHC images for NKX2-1 and HNF4α in subcutaneous tumors derived from allograft transplantation of 429A KN and KNH organoids into NRG mice, harvested at endpoint. Scale bar: 250 µm.

Supplemental Figure 2

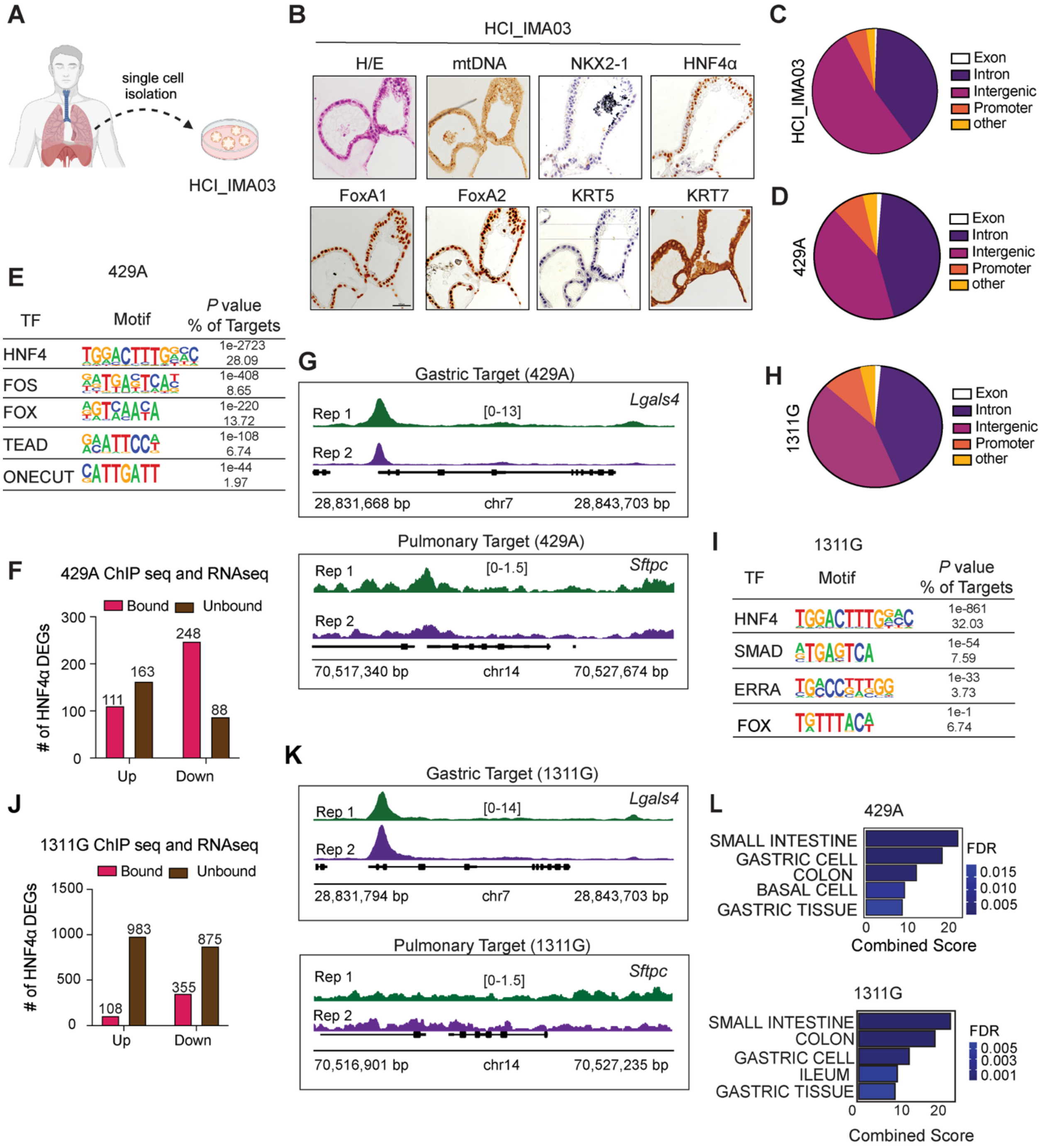

Supplemental Figure 2. HNF4α ChIP-seq and motif analyses in human and murine IMA models.

(**A**) Schematic of the derivation of HCI_IMA03 from an IMA tumor resected from a patient at Huntsman Cancer Institute. Created in BioRender. (**B**) Representative H&E and IHC images for human mitochondrion (mtDNA), NKX2-1, HNF4α, FoxA1, FoxA2, Cytokeratin 5 (KRT5), and Cytokeratin 7 (KRT7) in HCI_IMA03. Scale bar: 100 μm. (**C–D**) Genome-wide distribution of HNF4α ChIP-seq peaks in HCI_IMA03 (C) and 429A (D). (**E**) HOMER motif enrichment analysis of HNF4α-bound peaks in 429A, ranked by enrichment score and *P* value. (**F**) Overlap between HNF4α-bound regions and differentially expressed genes (DEGs) from bulk RNA-seq in 429A. (**G**) ChIP-seq tracks showing representative HNF4α binding at the gastric marker *Lgals4* and the pulmonary marker *Sftpc* in 429A. (**H–I**) Genome-wide distribution (H) and HOMER motif enrichment (I) of HNF4α ChIP-seq peaks in 1311G. (**J**) Overlap between HNF4α-bound regions and DEGs from bulk RNA-seq in 1311G. (**K**) ChIP-seq tracks showing representative HNF4α binding at *Lgals4* and *Sftpc* in 1311G. (**L**) ENRICHR ARCHS4 tissue enrichment of genes annotated from HNF4α peaks in 429A and 1311G.

Supplemental Figure 3

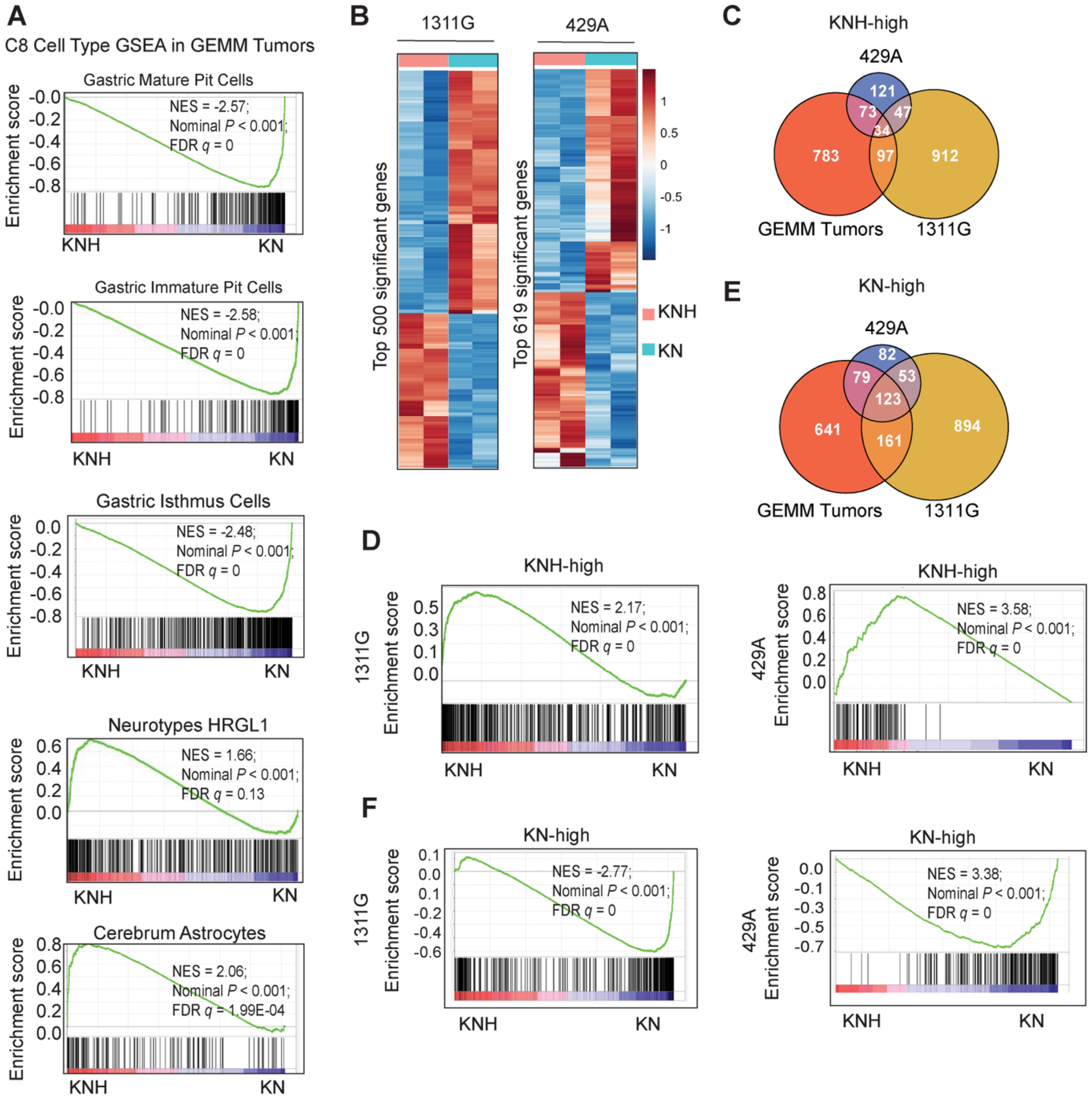

Supplemental Figure 3. Conserved transcriptional programs between GEMMs and organoid IMA models.

(**A**) GSEA of DEGs from bulk RNA-seq of KNH vs KN GEMM tumors showing enrichment of gene signatures corresponding to cell types gained or lost upon *Hnf4a* deletion, related to Figure 3A. NES and FDR are indicated. (**B**) Heatmaps of significant DEGs in isogenic IMA organoid lines: 1311G (Left: top 500 genes) and 429A (Right: top 619 genes). (**C**) Venn diagram illustrating the overlap of KNH-high DEGs identified in GEMM tumors and organoid lines (1311G and 429A). (**D**) GSEA plots showing enrichment of GEMM tumor-derived KNH-high DEGs in 1311G and 429A organoids. NES and FDR are indicated. (**E**) Venn diagram illustrating the overlap of KN-high DEGs identified in GEMM tumors and organoid lines (1311G and 429A). (**F**) GSEA plots showing enrichment of GEMM tumor-derived KN-high DEGs in 1311G and 429A organoids. NES and FDR are indicated.

Supplemental Figure 4

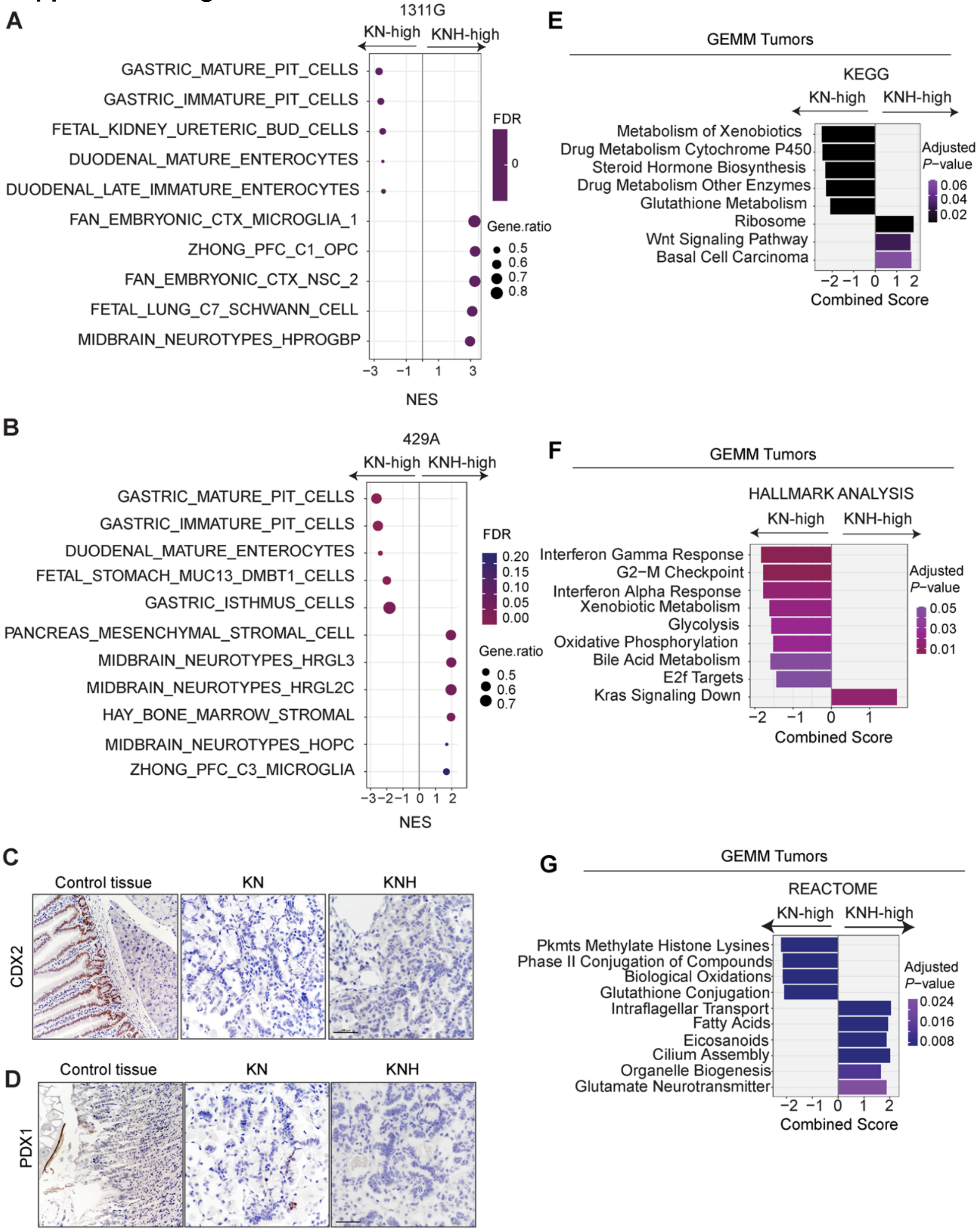

Supplemental Figure 4. Transcriptional and lineage alterations following HNF4α loss in IMA.

(**A–B**) GSEA of C8 cell type signatures using DEGs from 1311G (A) and 429A (B) organoids. (**C**) Representative IHC images for CDX2 in KN and KNH GEMM tumors. Representative control tissue is shown. Scale bar: 100 μm. (**D**) Representative IHC images for PDX1 in KN and KNH GEMM tumors. Representative control tissue is shown. Scale bar: 100 μm. (**E**) KEGG pathway enrichment of DEGs from KNH vs KN GEMM tumors. (**F**) Hallmark pathway enrichment of DEGs from KNH vs KN GEMM tumors. (**G**) Reactome pathway enrichment of DEGs from KNH vs KN GEMM tumors.

Supplemental Figure 5

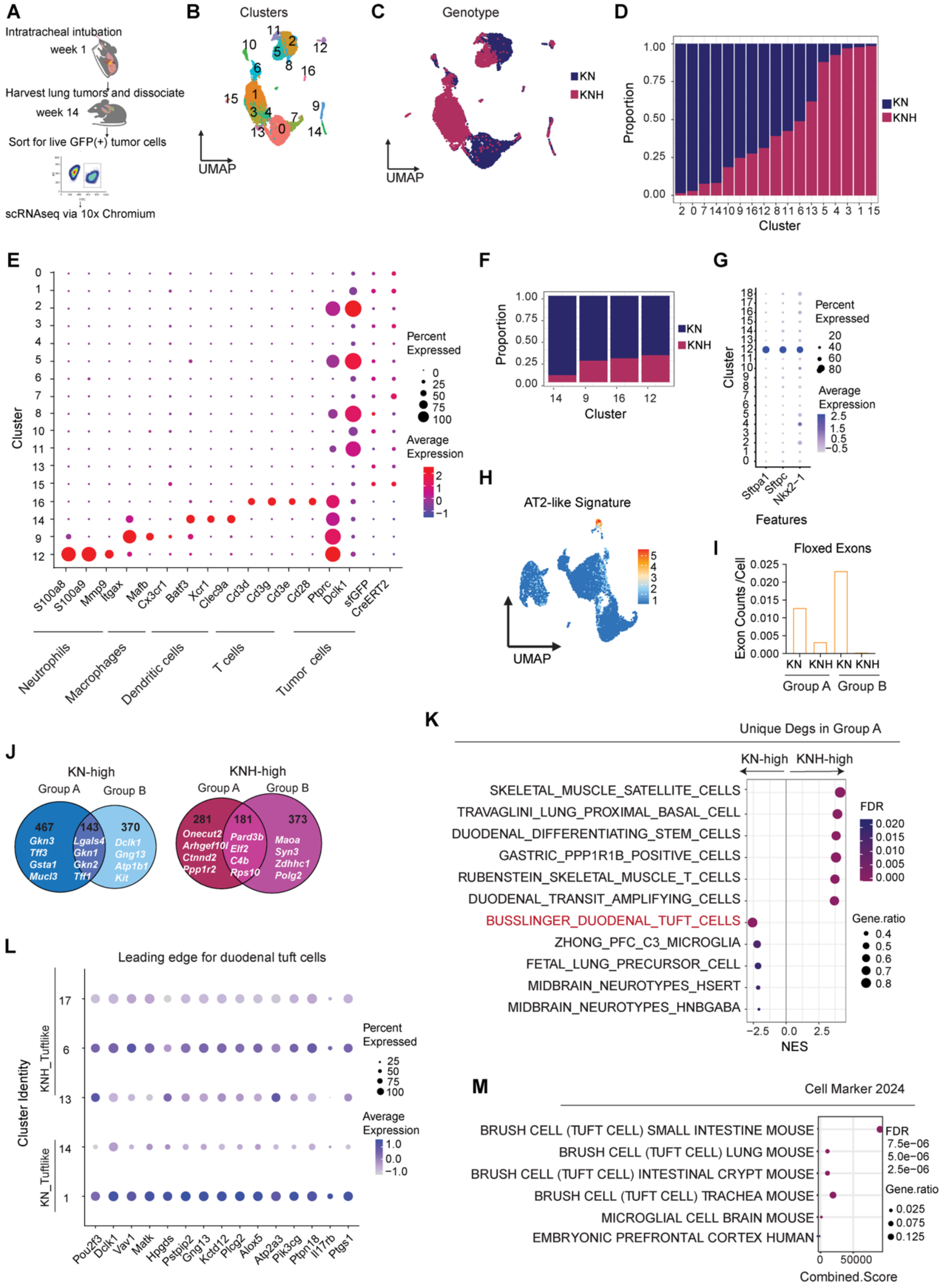

Supplemental Figure 5. Single-cell profiling reveals that loss of HNF4α alters gastric and tuft-like subpopulations in IMA tumors.

(**A**) Schematic of the experimental workflow for scRNA-seq of GFP-positive tumor cells sorted from KN and KNH GEMMs. Created in BioRender. (**B**) UMAP of all QC-passed cells (malignant and nonmalignant) captured from KN and KNH GEMM tumors (n = 2 mice per genotype; multiple tumors per mouse), colored by Seurat-defined clusters. (**C**) UMAP of the same dataset colored by genotype. (**D**) Proportion of KN and KNH cells across all captured cells. (**E**) Dot plot showing expression of immune-related and tumor-associated marker genes across Seurat-defined clusters. (**F**) Proportion of KN and KNH cells within immune-enriched clusters excluded from downstream analysis. (**G**) Dot plot showing the expression of alveolar type 2 (AT2) markers (*Sftpa1*, *Sftpc*, and *Nkx2-1*) across all bona fide tumor cells. (**H**) UMAPs showing AT2-like gene module scores based on a previously defined gene set (PMID: 32707077). (**I**) Number of reads per cell aligning to *Hnf4*a floxed exons 4 and 5 in KN vs KNH cells. (**J**) Venn diagrams showing overlapping DEGs between group A (tuft-like IMA) and group B (gastric-like IMA). Left: KNH-high DEGs. Right: KN-high DEGs. (**K**) GSEA-based C8 cell type enrichment analysis of unique DEGs in tuft-like IMA cells comparing KN and KNH GEMM tumors. (**L**) Dot plot showing expression of leading-edge genes contributing to the downregulation of the duodenal tuft cell gene signature in KNH vs KN GEMM tumors. (**M**) ENRICHR cell type enrichment analysis using Cell Marker 2024 on the leading-edge genes identified in panel L.

Supplemental Figure 6

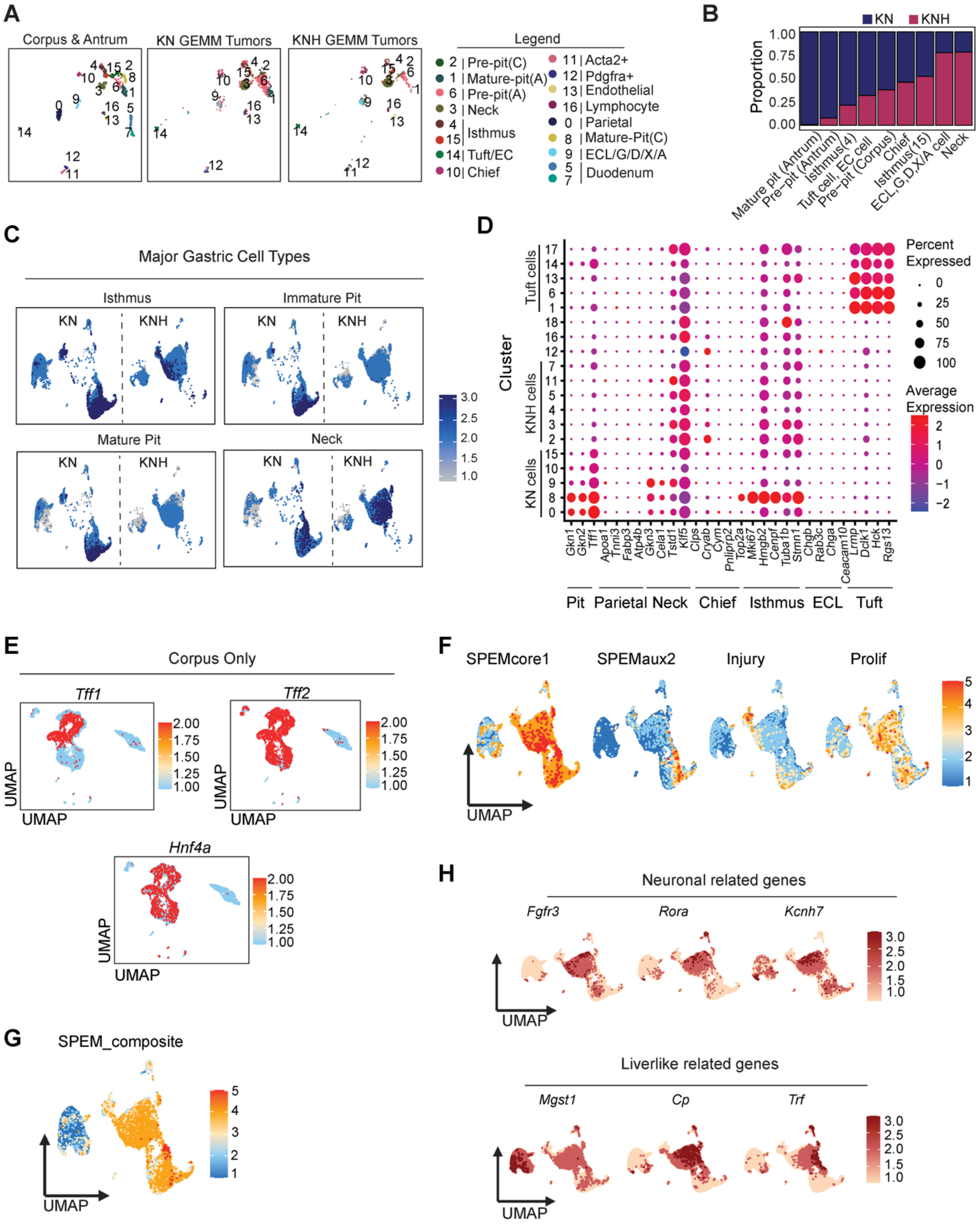

Supplemental Figure 6. Mapping KN and KNH IMA tumor cells to reference gastric datasets.

(**A**) UMAP of the reference corpus and antrum dataset (PMID: 37386010) showing annotated gastric epithelial and stromal cell types (left), alongside a UMAP of KN and KNH GEMM tumor cells mapped into the same reference space using Seurat label transfer. (**B**) Proportion of KN and KNH GEMM tumor cells mapping to each cluster in the corpus and antrum dataset shown in panel A. (**C**) UMAPs of gene module scores for major gastric cell types, including isthmus, immature pit, mature pit, and neck cells. (**D**) Dot plot of representative marker genes across major gastric cell types in IMA. (**E**) Feature plots of *Tff1*, *Tff2,* and *Hnf4a* expression in the corpus-only dataset (PMID: 37386010). (**F**) UMAPs showing gene module scores for individual SPEM-related gene signatures including SPEMcore, auxiliary secretory genes (SPEMaux2), injury/stress-response genes (Injury), and proliferation markers (Prolif). (**G**) UMAP of gene module scores for the composite SPEM signature. (**H**) Feature plots showing expression of neuronal-associated genes (*Fgfr3, Rora,* and *Kcnh7*) and liver-like related genes (*Mgst1, Cp,* and *Trf*) in KN and KNH GEMM tumors.

Supplemental Figure 7

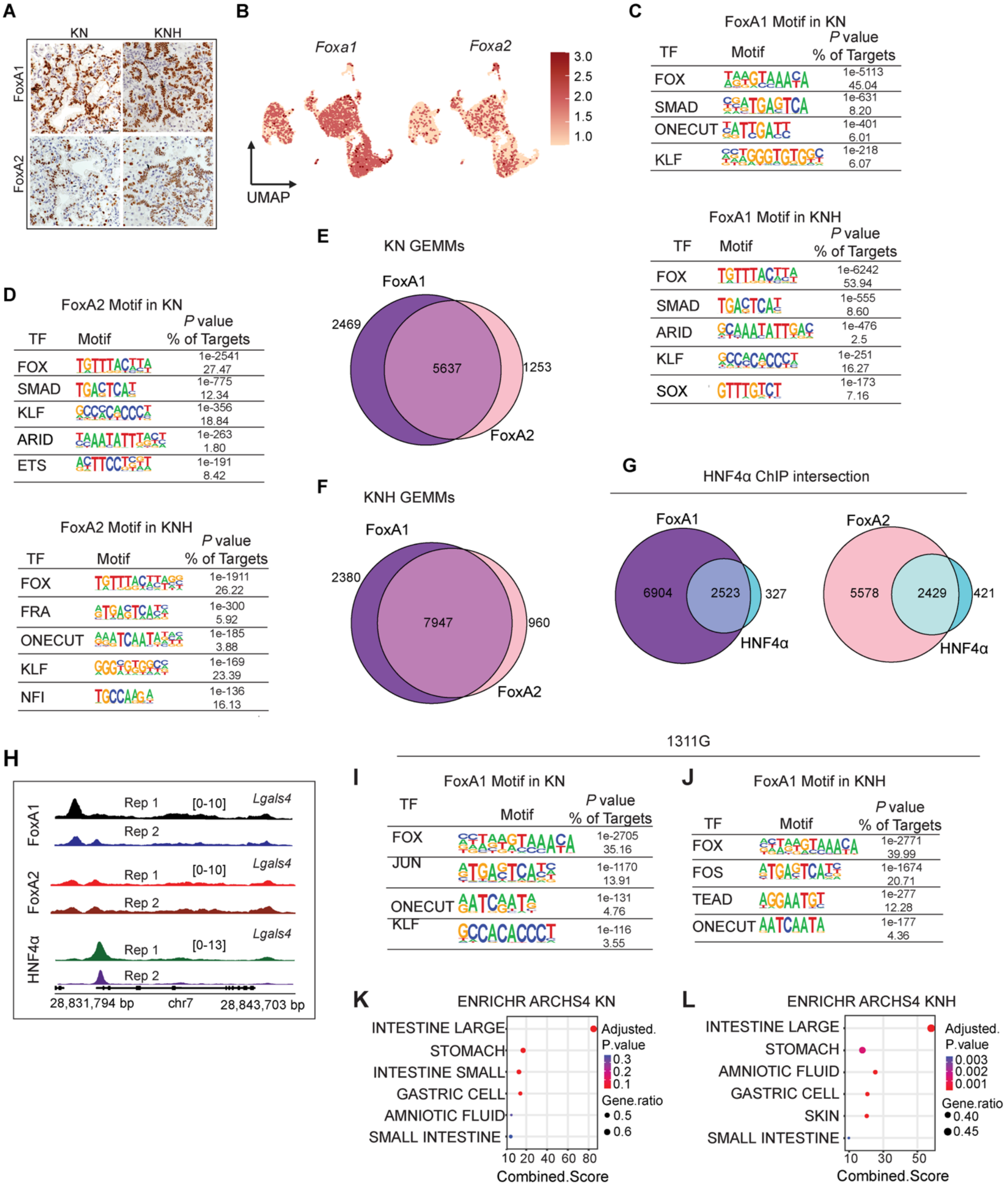

Supplemental Figure 7. HNF4α coordinates FoxA1/2 chromatin binding and gastric gene regulation in IMA.

(**A**) Representative IHC images for FoxA1 and FoxA2 in KN and KNH GEMM tumors at 14 weeks post-tumor initiation. Scale bar: 100 µm. (**B**) UMAP visualization of *FoxA1* and *FoxA2* expression across tumor cells, showing no obvious selective enrichment in either gastric or non-gastric regions. (**C–D**) HOMER motif enrichment of FoxA1-bound (C) and FoxA2-bound (D) peaks in KN and KNH GEMM tumors. Top, motifs enriched in KN-specific peaks; bottom, motifs enriched in KNH-specific peaks. (**E–F**) Overlap of annotated genes from FoxA1 and FoxA2 peaks in KN (E) and KNH (F) GEMM tumors. (**G**) Overlap of genes annotated from FoxA1 or FoxA2 peaks with those associated with HNF4α binding sites in KN GEMM tumors. (**H**) ChIP-seq tracks for FoxA1, FoxA2, and HNF4α in KN tumors showing co-occupancy at the gastric gene *Lgals4* (n = 2). (**I–J**) HOMER motif enrichment of genes annotated from FoxA1-bound peaks in 1311G KN (I) and KNH (J) organoids. (**K–L**) ENRICHR ARCHS4 tissue enrichment of genes annotated from FoxA1-bound peaks in 1311G KN (K) and KNH (L) organoids.

Supplemental Figure 8

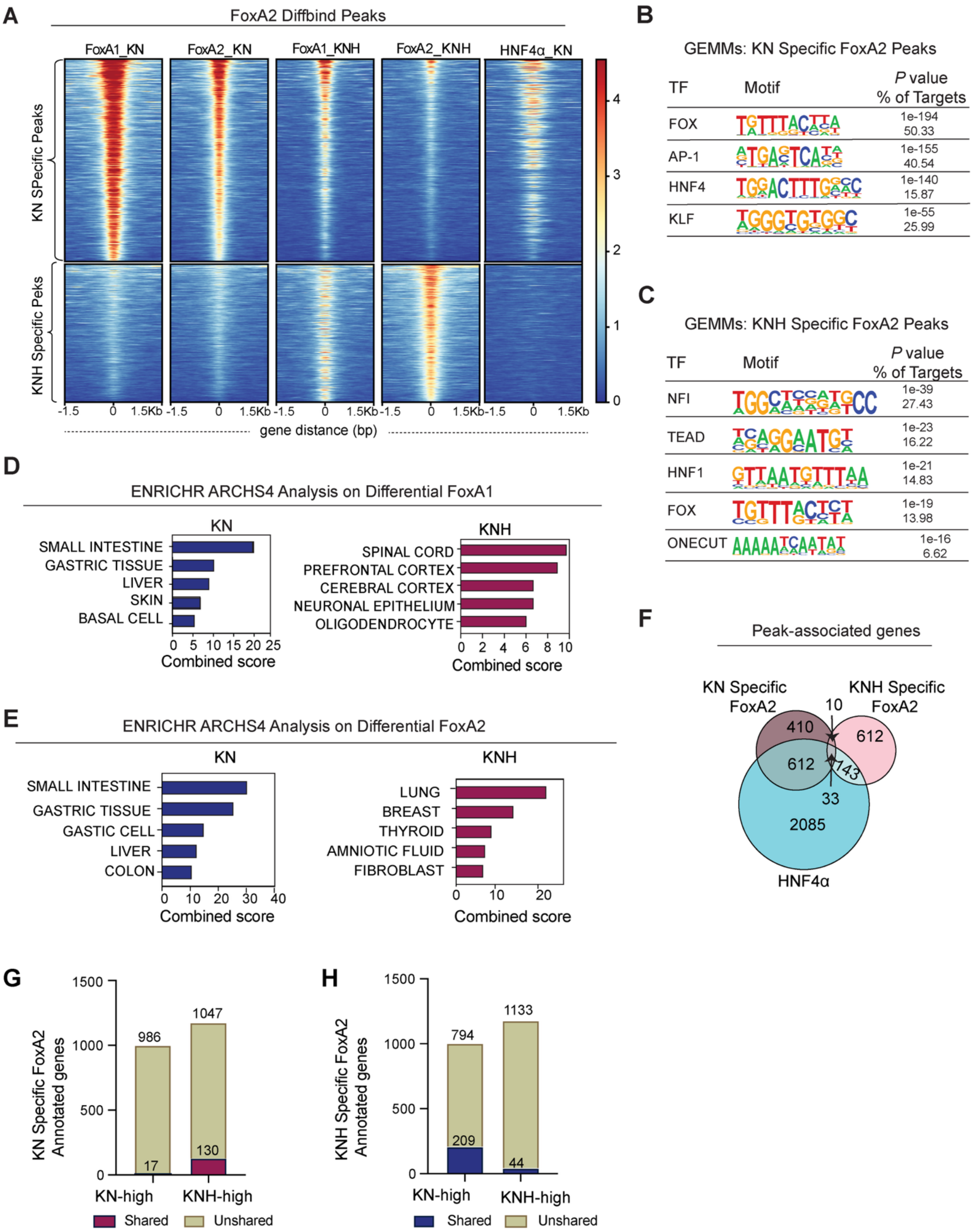

Supplemental Figure 8. FoxA2 phenocopies FoxA1 chromatin occupancy in vivo.

(**A**) Heatmap showing differential FoxA2 signal enrichment between KN and KNH GEMM tumors, identified using DiffBind (adjusted $p < 0.05$). Signal intensities were quantified over merged peak regions defined as significant peaks (MACS2, adjusted $P < 0.05$) detected in at least 1 condition, encompassing HNF4α, FoxA1, and FoxA2 peaks in KN tumors and FoxA1 and FoxA2 peaks in KNH tumors. (**B**) HOMER motif enrichment analysis of differential FoxA2 peaks in KN GEMM tumors. (**C**) HOMER motif enrichment analysis of differential FoxA2 peaks in KNH GEMM tumors. (**D**) ENRICHR ARCHS4 tissue enrichment of genes annotated from differential FoxA1 peaks. Left: KN-specific. Right: KNH-specific. (**E**) ENRICHR ARCHS4 tissue enrichment analysis of genes annotated from differential FoxA2 peaks. Left: KN-specific peaks. Right: KNH-specific peaks. (**F**) Venn diagram showing overlap between genes associated with differential FoxA2 peaks and genes linked to HNF4α-bound regions in KN GEMM tumors. (**G**) Bar plot showing overlap between genes annotated from KN-specific FoxA2 peaks and DEGs identified by bulk RNA-seq in GEMM tumors. (**H**) Bar plot showing overlap between genes annotated from KNH-specific FoxA2 peaks and DEGs identified by bulk RNA-seq in GEMM tumors.

Supplemental Figure 9

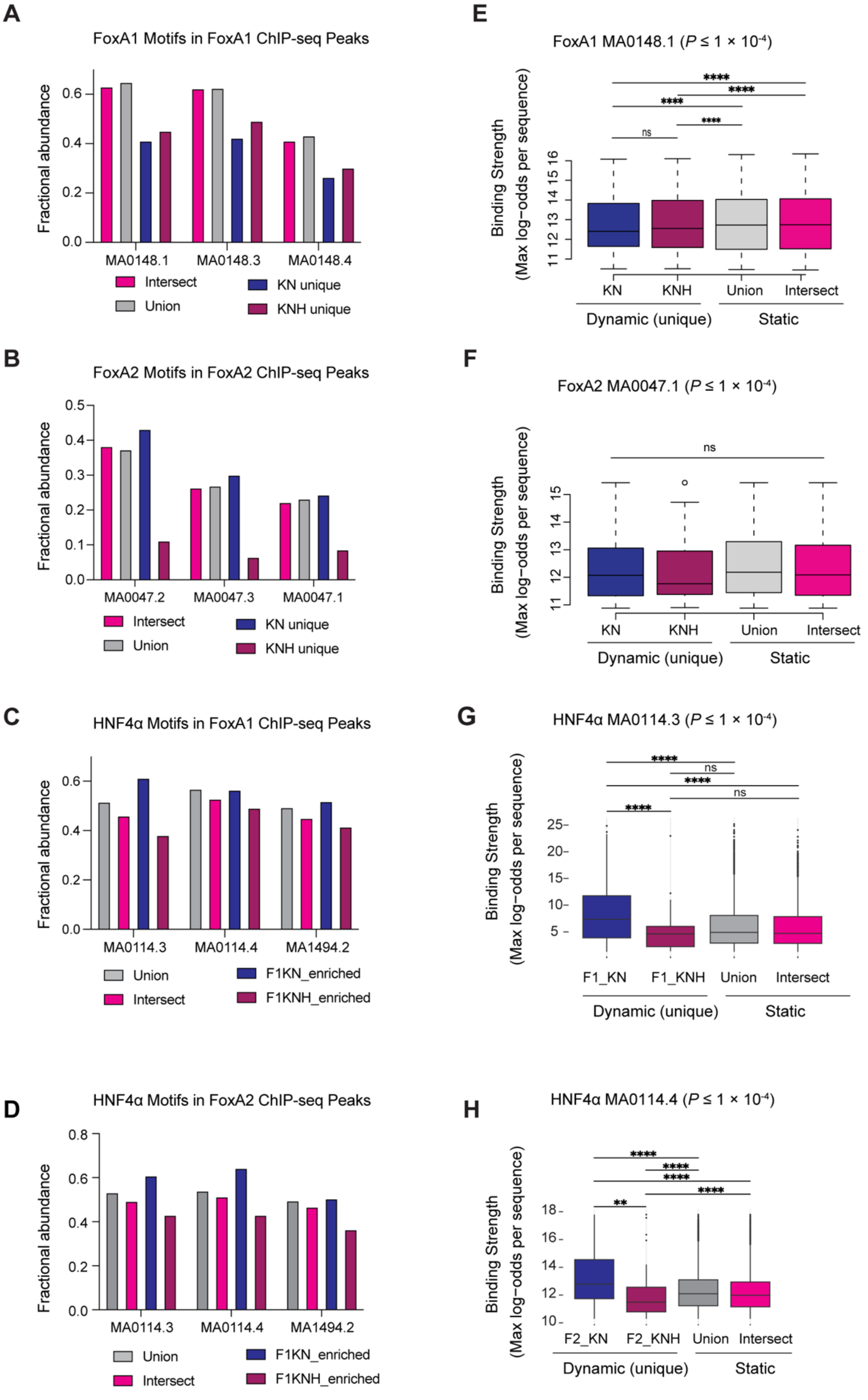

Supplemental Figure 9. Abundance and motif strength analysis of FoxA1, FoxA2 and HNF4α motifs in IMA.

(**A–D**) Fractional abundance of sequences containing representative FoxA1 (A) and FoxA2 (B) motifs, as well as HNF4α motifs within FoxA1 peaks (C) and FoxA2 peaks (D), across union, intersect, KN-unique, and KNH-unique peak sets. Fractional abundance was calculated as the proportion of sequences containing at least 1 motif identified by FIMO. Asterisks denote FDR-adjusted q values: ns, $q \geq 0.05$; * $q < 0.05$; ** $q < 0.01$; *** $q < 0.001$; **** $q < 0.0001$. (**E–H**) Motif strength analysis showing the maximum log-odds score per sequence for representative FoxA1 (E) and FoxA2 (F) motifs, as well as HNF4α motifs within FoxA1 peaks (G) and FoxA2 peaks (H), across KN-unique, KNH-unique, union, and intersect peak sets. Motifs were identified using FIMO with a significance threshold of $P \leq 1 \times 10^{-4}$. For each sequence, the highest log-odds score among all detected motif instances was used as the motif strength metric. Asterisks denote FDR-adjusted q values: ns, $q \geq 0.05$; * $q < 0.05$; ** $q < 0.01$; *** $q < 0.001$; **** $q < 0.0001$.

Supplemental Figure 10

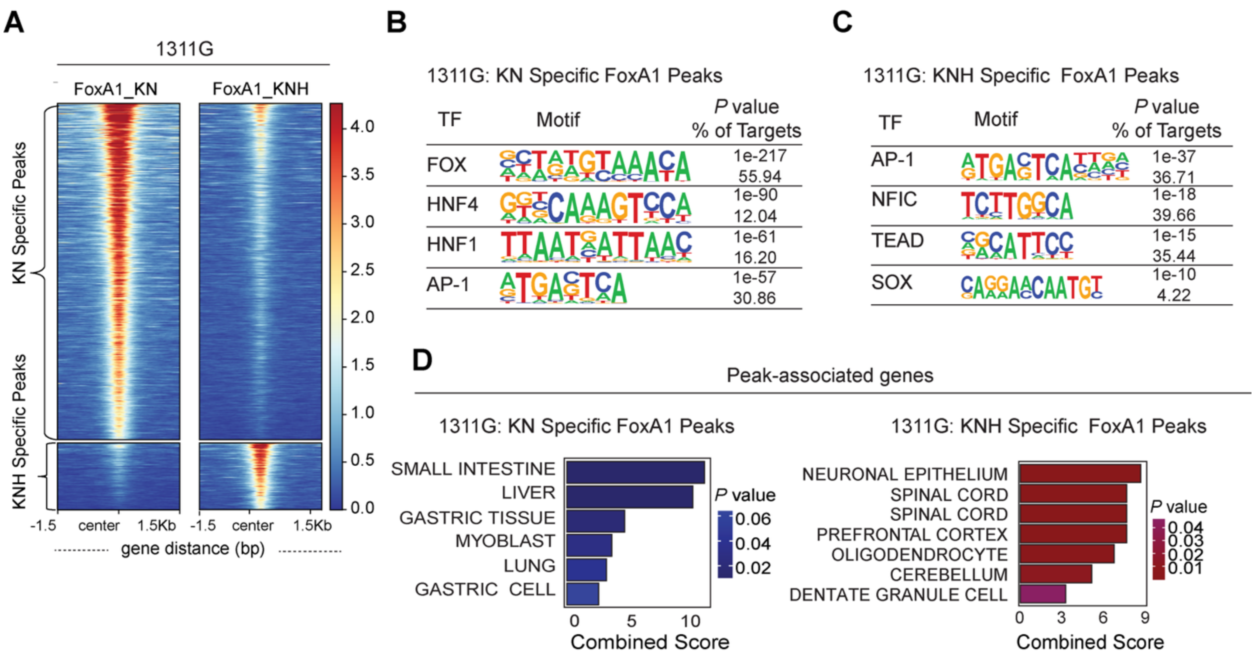

Supplemental Figure 10. HNF4α loss redistributes FoxA1 occupancy in 1311G organoids.

(**A**) Heatmap showing FoxA1 occupancy at differential binding sites in 1311G KN and KNH organoids. DiffBind analysis identified 1,799 KN-specific and 337 KNH-specific peaks. (**B**) HOMER motif enrichment analysis of differential FoxA1 peaks identified in 1311G KN organoids. (**C**) HOMER motif enrichment analysis of differential FoxA1 peaks identified in 1311G KNH organoids. (**D**) ENRICHR ARCHS4 tissue enrichment of genes annotated from differential FoxA1 peaks. Left: 1311G KN-specific peaks. Right: 1311G KNH-specific peaks.

Supplemental Figure 11

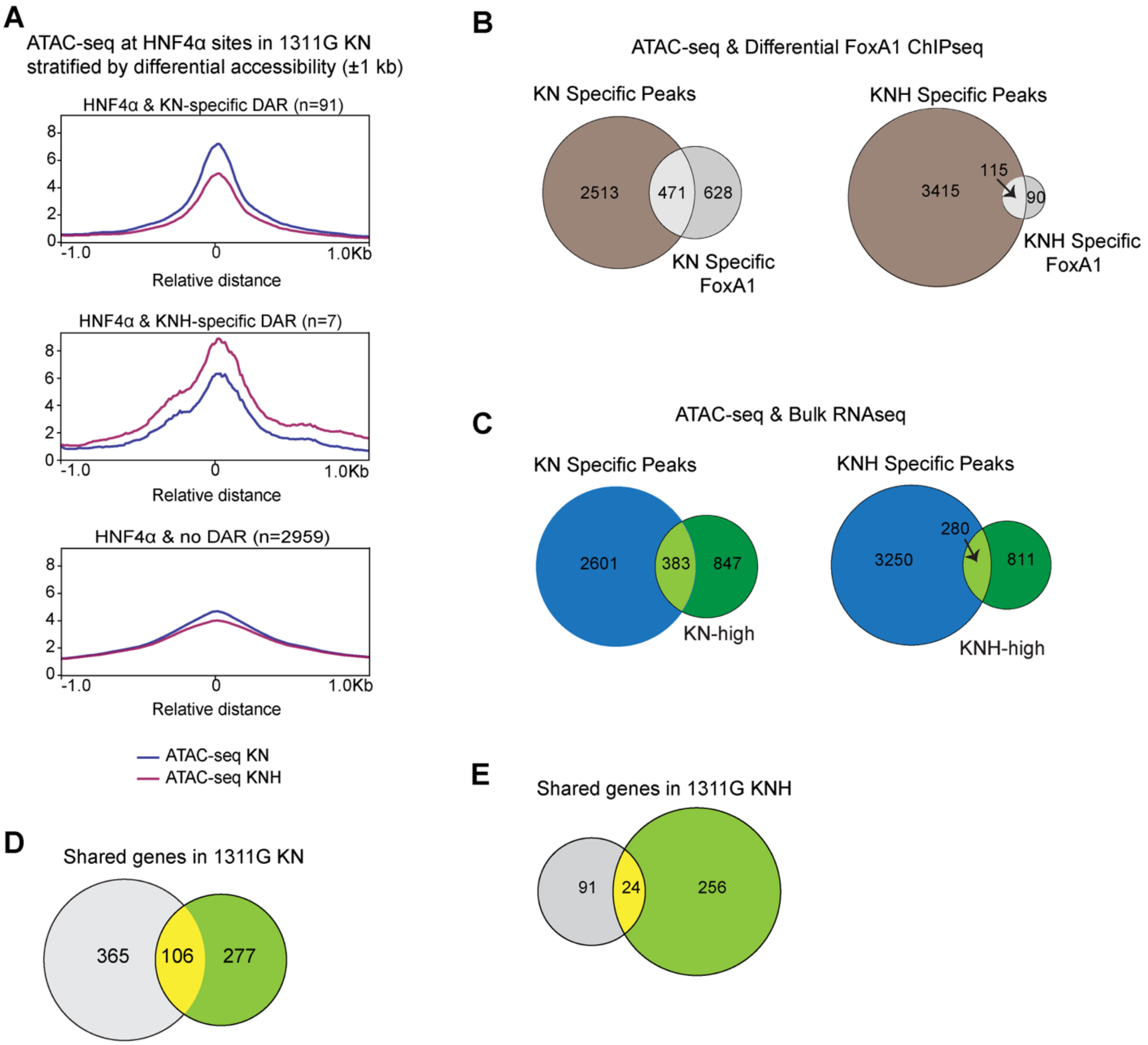

Supplemental Figure 11. Integrated epigenomic and transcriptomic analyses define genotype-specific regulatory networks in IMA.

(**A**) Mean ATAC-seq signal in 1311G KN and KNH centered on HNF4α summits in 1311G KN (±1 kb) after stratifying summits by overlap with DiffBind-defined ATAC differentially accessible regions, including KN-specific DARs (n = 91), KNH-specific DARs (n = 7), and sites not overlapping any DAR (n = 2959). (**B**) Venn diagrams showing overlap between genes annotated from KN-specific ATAC-seq peaks and KN-specific FoxA1-bound peaks in 1311G (left), and between genes annotated from KNH-specific ATAC-seq peaks and KNH-specific FoxA1-bound peaks in 1311G (right). (**C**) Venn diagrams showing overlap between genes annotated from KN-specific ATAC-seq peaks and KN-high DEGs from bulk RNA-seq in 1311G (left), and between genes annotated from KNH-specific ATAC-seq peaks and KNH-high DEGs from bulk RNA-seq in 1311G (right). (**D**) Venn diagram showing overlap between genes shared in 1311G KN tumors identified in panels B (471 shared genes) and C (383 shared genes), integrating ATAC-seq, bulk RNA-seq, and ChIP-seq datasets. Only genes common across all three modalities are shown. (**E**) Venn diagram showing overlap between genes shared in 1311G KNH tumors identified in panels B (115 shared genes) and C (280 shared genes), integrating ATAC-seq, bulk RNA-seq, and ChIP-seq datasets. Only genes common across all three modalities are shown.

Supplemental Figure 12

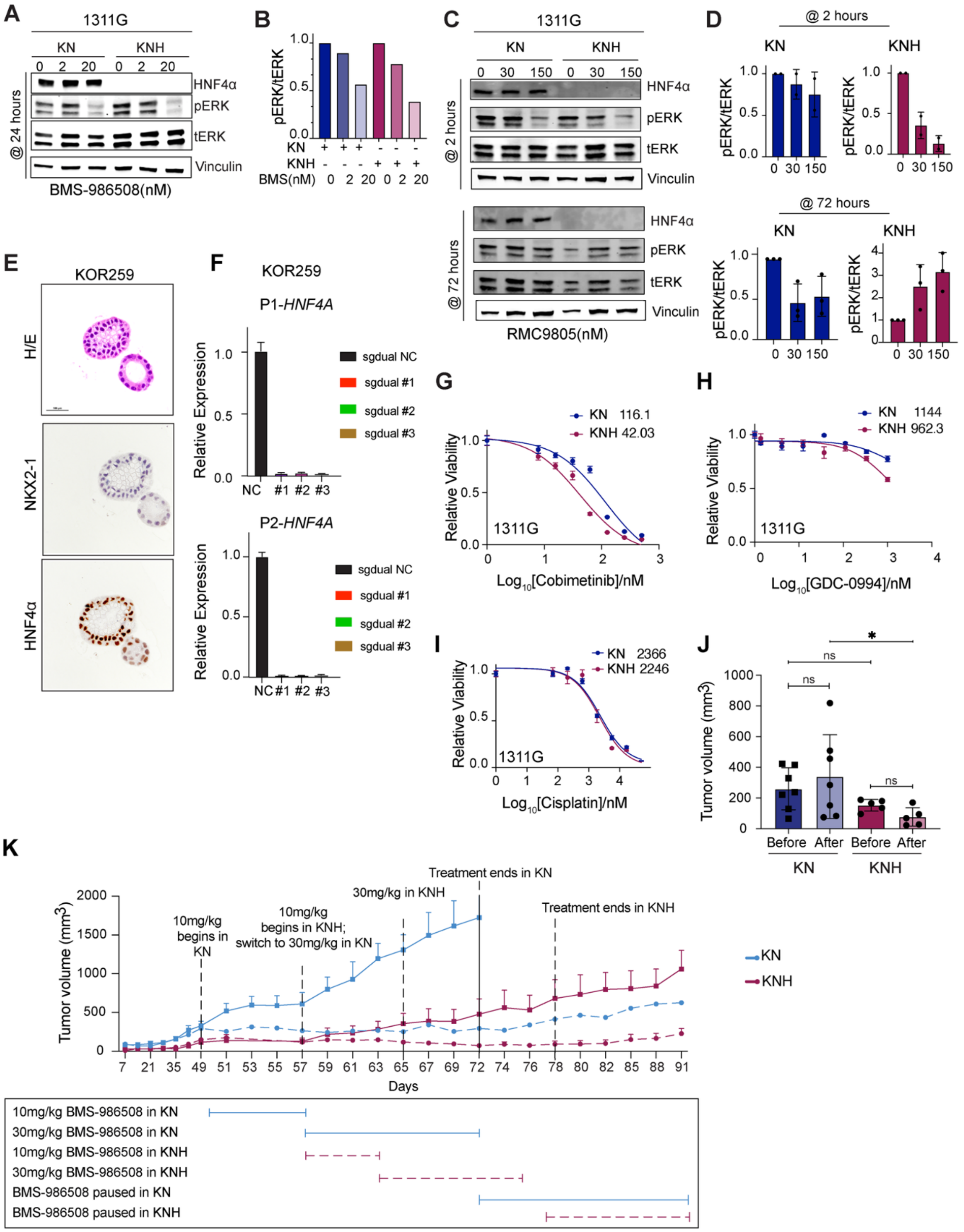

Supplemental Figure 12. HNF4α loss enhances sensitivity to MAPK pathway inhibition in IMA.

(**A**) Representative immunoblot analysis of the indicated proteins in 1311G KN and KNH organoids treated for 24 hours with vehicle or increasing doses of BMS-986508. (**B**) Quantification of pERK normalized to total ERK from panel A. (**C**) Representative immunoblot analysis of the indicated proteins in 1311G KN and KNH organoids treated with RMC-9805 for 2 or 72 hours. (**D**) Quantification of pERK normalized to total ERK from panel C. Data represent n = 2 (2 hours) or n = 3 (72 hours) independent biological replicates and are shown as mean ± SD. (**E**) Representative H&E and IHC images showing NKX2-1 and HNF4α expression in KOR259 organoids. Scale bar: 100 µm. (**F**) qPCR analysis of P1 and P2 *HNF4A* isoforms in KOR259 organoids following lentiviral transduction with dual guide RNAs targeting P1/P2 *HNF4A* or a nontargeting control. Selection was performed for 4 days. (**G**) Dose-response curve for 1311G KN and KNH organoids treated with cobimetinib. Calculated IC_{50} values were 116.1 nM for KN and 42.03 nM for KNH. Data shown represent one of three independent biological replicates; error bars indicate SEM. (**H**) Dose-response curves for 1311G KN and KNH organoids treated with GDC-0994. Calculated IC_{50} values were 1144 nM for KN and 962.3 nM for KNH. Data shown represent one of three independent biological replicates; error bars indicate SEM. (**I**) Dose-response curves for 1311G KN and KNH organoids treated with cisplatin. Calculated IC_{50} values were 2366 nM for KN and 2246 nM for KNH. Data shown represent one of three independent biological replicates; error bars indicate SEM. (**J**) Tumor volumes from individual NSG mice bearing 1311G KN or KNH allografts measured before and after treatment with BMS-986508. Baseline tumor volumes were comparable between genotypes (2-tailed Mann-Whitney test, ns, P = 0.2020), whereas tumor volumes after treatment were significantly lower in KNH than in KN allografts (2-tailed Mann-Whitney test, P = 0.0480). Comparisons of tumor volume before and after treatment within each genotype were assessed by 2-tailed Wilcoxon matched-pairs signed-rank test (KN: ns, P = 0.3750; KNH: P = 0.0625). Error bars represent SD.(**K**) Longitudinal tumor volume measurements from NSG mice implanted subcutaneously with 1311G KN or KNH organoids (4×10^5 cells). Treatment was initiated when tumors reached approximately 150 mm^3. Mice received vehicle or BMS-986508 (10 mg/kg for 7 days followed by 30 mg/kg for 14 days), followed by a drug-paused period. Error bars represent SEM.

Supplemental Figure 13

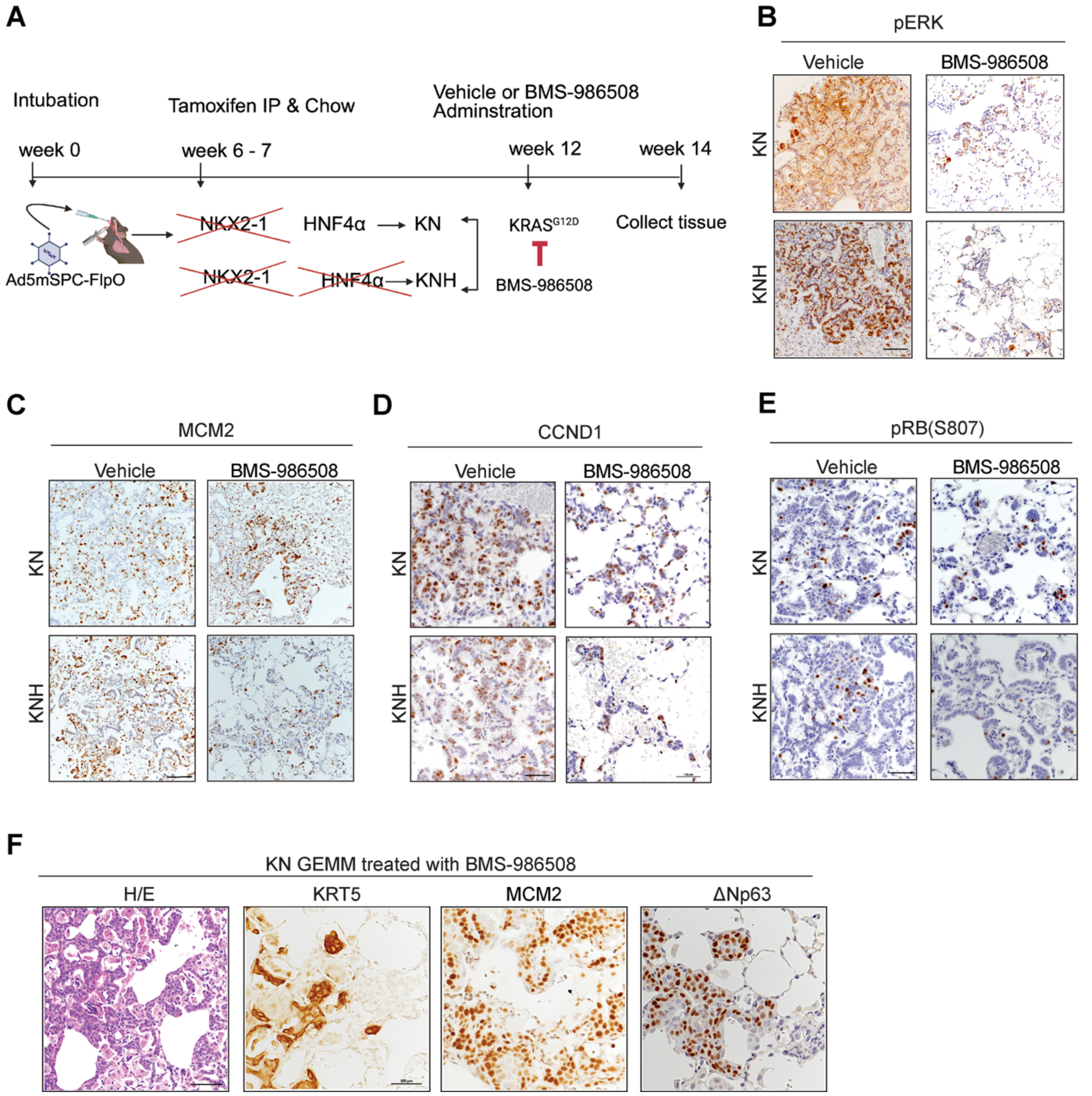

Supplemental Figure 13. HNF4α sustains proliferative persistence during KRAS^{G12D} inhibition in vivo.

(**A**) Schematic of the in vivo BMS-986508 treatment protocol in GEMMs. Created in BioRender. (**B**) Representative IHC images of phospho-ERK (pERK) in tumors from KN and KNH GEMMs treated with vehicle or BMS-986508 (30 mg/kg) for 14 days. Scale bar: 100 μm. (**C–E**) Representative IHC images of proliferation and cell cycle markers in tumors from KN and KNH GEMMs treated with vehicle or BMS-986508 (30 mg/kg) for 14 days. Markers shown are MCM2 (C), cyclin D1 (CCND1) (D), and phospho-RB (Ser807) (pRB(S807)) (E). Scale bar: 100 μm. (**F**) Representative H&E and IHC images of cytokeratin 5 (KRT5), MCM2, and ΔNp63 in a KN GEMM tumor treated with BMS-986508 (30 mg/kg) for 14 days. Scale bar: 100 μm.

Supplemental Figure 14

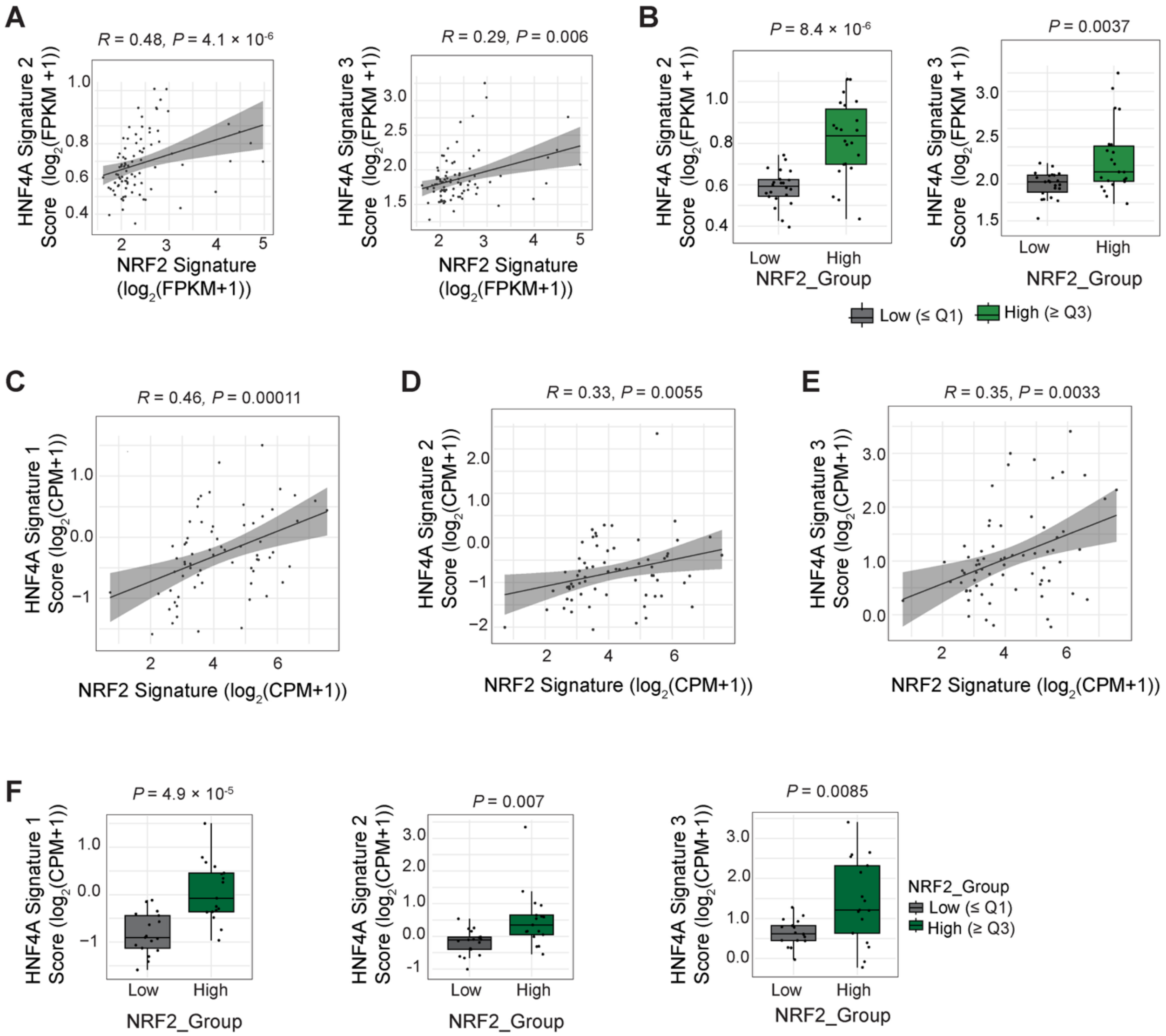

Supplemental Figure 14. Association between NRF2 and HNF4A activity scores and functional impact of NRF2 expression in IMA.

(**A**) Spearman correlation between NRF2 activity and HNF4A signature 2 and 3 scores in KRAS-mutant NSCLC (TCGA); each dot represents a tumor. Spearman correlation coefficient (*R*) and *P* values are shown. Linear regression lines with 95% confidence intervals are overlaid. (**B**) Boxplots comparing HNF4A signature 2 and 3 scores in NRF2-low and NRF2-high tumors; *P* values were determined using unpaired two-tailed Student's *t* test. (**C–E**) Spearman correlation analyses between NRF2 activity and HNF4A signature scores across 68 KRAS-mutant non–small cell lung cancer tumors from the KRYSTAL-1 dataset (PMID: 39804166). NRF2 activity was quantified using a published NRF2 gene signature. HNF4A activity was assessed using three independent gene signatures: Signature 1 (C), Signature 2 (D), and Signature 3 (E). Each dot represents an individual tumor. Gray lines indicate linear regression with 95% confidence intervals. Spearman correlation coefficients (*R*) and *P* values are shown. (**F**) Boxplots comparing HNF4A signature 1–3 activity scores between NRF2-low and NRF2-high tumors. *P* values were determined using unpaired two-tailed Student's *t* test.

Supplemental Figure 15

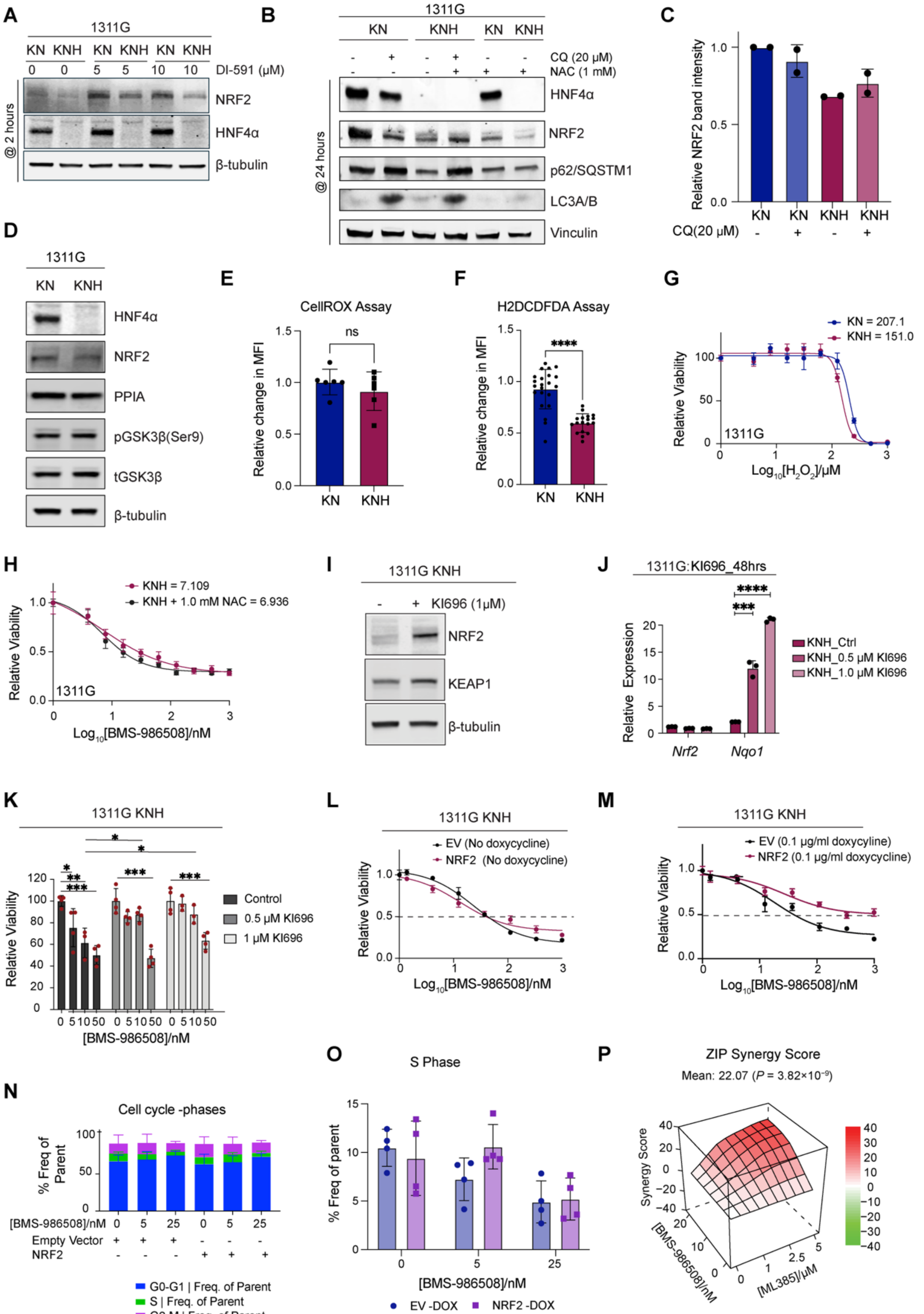

Supplemental Figure 15. Pharmacological activation and genetic modulation of the NRF2 pathway.

(**A**) Representative immunoblot of the indicated proteins in 1311G KN and KNH organoids treated with vehicle (0), 5 μM, or 10 μM DI-591 for 2 hours. Data are representative of 3 independent biological replicates. (**B**) Representative immunoblot of the indicated proteins in KN and KNH organoids following treatment with 20 μM chloroquine (CQ) or 1 μM N-acetylcysteine (NAC) for 24 hours. (**C**) Quantification of NRF2 protein levels normalized to the loading control and untreated organoids from panel B. (**D**) Representative immunoblot of the indicated proteins in KN and KNH organoids. Data are representative of 3 independent biological replicates. (**E**) Quantification of intracellular reactive oxygen species (ROS) levels in 1311G KN and KNH organoids measured using the CellROX assay. Data are pooled from 3 independent biological replicates and are shown as mean ± SD. (**F**) Quantification of intracellular ROS levels in 1311G KN and KNH organoids measured using the H_2DCFDA assay. Data are pooled from 4 independent biological replicates and are shown as mean ± SD. Unpaired Student's *t* test (*****P* < 0.0001). (**G**) Cell viability of 1311G KN and KNH organoids following 72-hour treatment with serial dilutions of H_2O_2. Calculated IC_{50} values were 207.1 μM (KN) and 151 μM (KNH). Data represent 1 of 2 independent biological replicates; error bars indicate SEM of technical replicates. (**H**) Cell viability of 1311G KNH organoids treated with BMS-986508 ± 1 mM NAC for 72 hours. IC_{50} values are indicated. Data represent 1 of 2 independent biological replicates; error bars indicate SEM of technical replicates. (**I**) Representative immunoblot of the indicated proteins in 1311G KNH organoids treated with 1 μM KI696 for 48 hours. Data are representative of 3 independent biological replicates. (**J**) qRT-PCR analysis of *NFE2L2* and *Nqo1* in 1311G KNH organoids treated with increasing doses of KI696 for 48 hours (n = 2; representative shown). One-way ANOVA (****P* = 0.0003, **** *P* < 0.0001). (**K**) NRF2 activation by KI696 reduces sensitivity of 1311G KNH organoids to BMS-986508. Organoids were pretreated with vehicle (0), 0.5 μM or 1 μM KI696 for 7 days, followed by combination treatment with KI696 and BMS-986508 for 3 days (**P* < 0.05, ***P* < 0.01, ****P* < 0.001). Data are representative of 3 independent biological replicates. (**L–M**) Effect of NRF2 expression on BMS-986508 sensitivity in 1311G KNH organoids. Organoids expressing empty vector (EV) or doxycycline-inducible NRF2 were treated with BMS-986508 for 72 hours in the absence (L) or presence (M) of doxycycline (0.1 μg/mL). IC_{50} values increased from 2.5 nM (EV) to 14.2 nM upon NRF2 induction with doxycycline. Data shown are representative of 1 of 3 independent experiments. Error bars represent SEM. (**N**) Quantification of cell cycle distribution in 1311G KNH organoids transduced with a doxycycline-inducible NRF2 expression construct or empty vector (EV) control. Data are shown as mean ± SD from n = 4 independent biological replicates. (**O**) S phase frequency (%) of the parent population in EV or NRF2-expressing cells treated with increasing concentrations of BMS-986508 (0, 5, and 25 nM). 2-way ANOVA showed a significant effect of concentration (*P* = 0.0015), with no effect of group (*P* = 0.98) or interaction (*P* = 0.75). Data are shown as mean ± SD from n = 4 independent biological replicates. (**P**) ZIP synergy score calculated for the data shown in Main Figure 9L using SynergyFinder.

Supplemental Figure 16

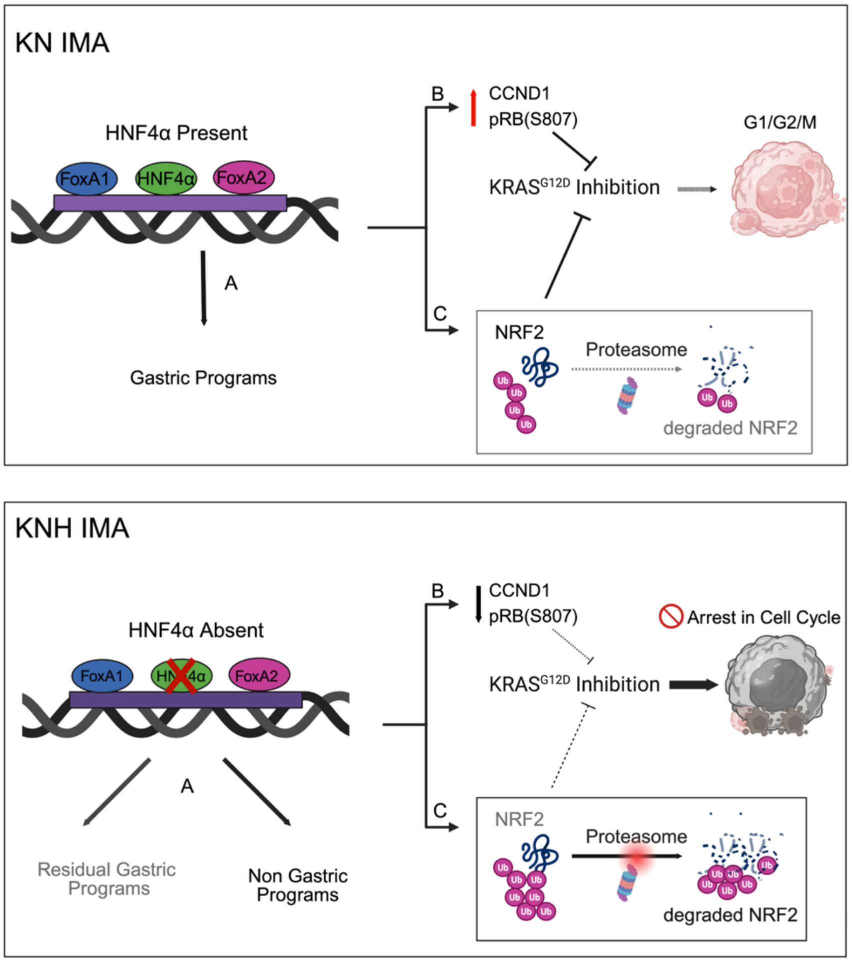

Supplemental Figure 16. Working model of HNF4α role in IMA.

Schematic working model illustrating the role of HNF4α as a central regulator of gastric lineage identity (A) and primary response to $KRAS^{G12D}$ inhibition via the NRF2 axis in IMA (B and C).
KN IMA: HNF4α maintains gastric lineage programs and stabilizes NRF2, which provides a cytoprotective buffer against $KRAS^{G12D}$ inhibition, allowing continued cell cycle progression.
KNH IMA: Loss of HNF4α reprograms transcriptional networks, promotes ubiquitin-mediated proteasomal degradation of NRF2, and sensitizes cells to $KRAS^{G12D}$ inhibitors, resulting in cell cycle arrest. Created in BioRender.

REFERENCES

1. Jackson EL, et al. Analysis of lung tumor initiation and progression using conditional expression of oncogenic k-ras. *Genes Dev.* 2001;15:3243-8.
2. Young NP, et al. Uncoupling cancer mutations reveals critical timing of p53 loss in sarcomagenesis. *Cancer Research.* 2011;71:4040-7.
3. Schönhuber N, et al. A next-generation dual-recombinase system for time- and host-specific targeting of pancreatic cancer. *Nat Med.* 2014;20:1340-7.
4. Kusakabe T, et al. Thyroid-specific enhancer-binding protein/nkx2.1 is required for the maintenance of ordered architecture and function of the differentiated thyroid. *Mol. Endocrinol.* 2006;20:1796-809.
5. Hayhurst GP, et al. Hepatocyte nuclear factor 4α (nuclear receptor 2a1) is essential for maintenance of hepatic gene expression and lipid homeostasis. *Mol. Cell. Biol.* 2001;21:1393-403.
6. Mo A, et al. Epigenomic signatures of neuronal diversity in the mammalian brain. *Neuron.* 2015;86:1369-84.
7. Lee CL, et al. Generation of primary tumors with flp recombinase in frt-flanked p53 mice. *Dis Model Mech.* 2012;5:397-402.
8. Li F, et al. Efficient genetic manipulation of the nod-rag1-/-il2rgammac-null mouse by combining in vitro fertilization and crispr/cas9 technology. *Sci. Rep.* 2014;4:5290.
9. Shultz LD, et al. Human lymphoid and myeloid cell development in nod/ltsz-scid il2r gamma null mice engrafted with mobilized human hemopoietic stem cells. *J. Immunol.* 2005;174:6477-89.
10. Miyoshi H, Stappenbeck TS. In vitro expansion and genetic modification of gastrointestinal stem cells in spheroid culture. *Nat. Protoc.* 2013;8:2471-82.
11. Ebisudani T, et al. Genotype-phenotype mapping of a patient-derived lung cancer organoid biobank identifies nkx2-1-defined wnt dependency in lung adenocarcinoma. *Cell Rep.* 2023;42:112212.
12. Pleguezuelos-Manzano C, et al. Establishment and culture of human intestinal organoids derived from adult stem cells. *Curr. Protoc. Immunol.* 2020;130:e106.
13. Fang P, et al. Differential control of growth and identity by hnf4α isoforms in pancreatic ductal adenocarcinoma. *Mol. Cancer Res.* 2025;23:936-52.
14. Dobin A, et al. Star: Ultrafast universal rna-seq aligner. *Bioinformatics.* 2012;29:15-21.
15. Bushnell B. Bbtools software packag. *e.* 2014;
16. Liao Y, et al. The r package rsubread is easier, faster, cheaper and better for alignment and quantification of rna sequencing reads. *Nucleic Acids Res.* 2019;47:e47-e.
17. Love MI, et al. Moderated estimation of fold change and dispersion for rna-seq data with deseq2. *Genome Biol.* 2014;15:1-21.
18. Subramanian A, et al. Gene set enrichment analysis: A knowledge-based approach for interpreting genome-wide expression profiles. *Proceedings of the National Academy of Sciences.* 2005;102:15545-50.
19. Liberzon A, et al. Molecular signatures database (msigdb) 3.0. *Bioinformatics.* 2011;27:1739-40.
20. Hao Y, et al. Integrated analysis of multimodal single-cell data. *Cell.* 2021;184:3573-87.e29.
21. Gulati GS, et al. Single-cell transcriptional diversity is a hallmark of developmental potential. *Science.* 2020;367:405-11.

22. Linderman GC, et al. Zero-preserving imputation of single-cell rna-seq data. *Nature Commun.* 2022;13:192.
23. Langmead B, Salzberg SL. Fast gapped-read alignment with bowtie 2. *Nature Methods.* 2012;9:357-9.
24. Zhang Y, et al. Model-based analysis of chip-seq (macs). *Genome Biol.* 2008;9:R137.
25. Quinlan AR, Hall IM. Bedtools: A flexible suite of utilities for comparing genomic features. *Bioinformatics.* 2010;26:841-2.
26. Heinz S, et al. Simple combinations of lineage-determining transcription factors prime cis-regulatory elements required for macrophage and b cell identities. *Mol. Cell.* 2010;38:576-89.
27. Ross-Innes CS, et al. Differential oestrogen receptor binding is associated with clinical outcome in breast cancer. *Nature.* 2012;481:389-93.
28. Ramírez F, et al. Deeptools2: A next generation web server for deep-sequencing data analysis. *Nucleic Acids Res.* 2016;44:W160-W5.
29. Chen EY, et al. Enrichr: Interactive and collaborative html5 gene list enrichment analysis tool. *BMC Bioinformatics.* 2013;14:128.
30. Granja JM, et al. Single-cell multiomic analysis identifies regulatory programs in mixed-phenotype acute leukemia. *Nat. Biotechnol.* 2019;37:1458-65.
31. Grant CE, et al. Fimo: Scanning for occurrences of a given motif. *Bioinformatics.* 2011;27:1017-8.
32. Geusz RJ, et al. Sequence logic at enhancers governs a dual mechanism of endodermal organ fate induction by foxa pioneer factors. *Nat Commun.* 2021;12:6636.
33. Corces MR, et al. An improved atac-seq protocol reduces background and enables interrogation of frozen tissues. *Nature Methods.* 2017;14:959-62.
34. Buenrostro JD, et al. Transposition of native chromatin for fast and sensitive epigenomic profiling of open chromatin, DNA-binding proteins and nucleosome position. *Nature Methods.* 2013;10:1213-8.
35. Fort G, et al. Opposing lineage specifiers induce a protumor hybrid identity state in lung adenocarcinoma. *Genes Dev.* 2025;39:1081-105.
36. Negrao MV, et al. Impact of co-mutations and transcriptional signatures in non-small cell lung cancer patients treated with adagrasib in the krystal-1 trial. *Clin. Cancer Res.* 2025;31:1069-81.
37. Singh A, et al. Nrf2 activation promotes aggressive lung cancer and associates with poor clinical outcomes. *Clin. Cancer Res.* 2021;27:877-88.
38. Tong X, et al. Adeno-to-squamous transition drives resistance to kras inhibition in lkb1 mutant lung cancer. *Cancer Cell.* 2024;42:413-28.e7.
