# Supplementary material for: HNF4α controls growth, identity, and KRAS inhibitor response in invasive mucinous adenocarcinoma of the lung
Source: J Clin Invest. 2026 May 12;136(13):e198282. doi: 10.1172/JCI198282 (PMC13318121; doi:10.1172/JCI198282)

A

Figure 1D - 1311G

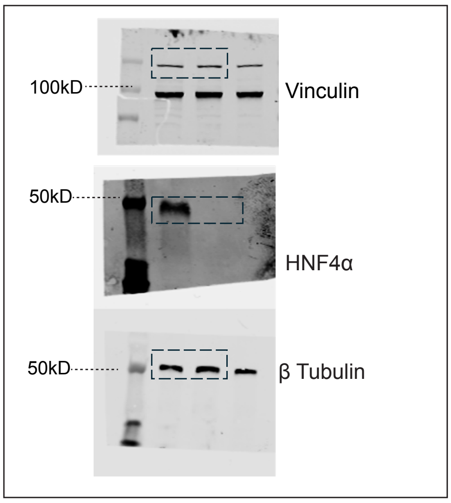

B

Figure 1D - 429A

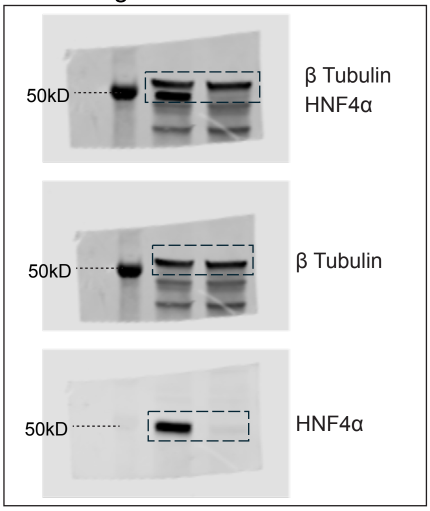

C

Figure 8D

BMS968508(nM) @ 2 hours

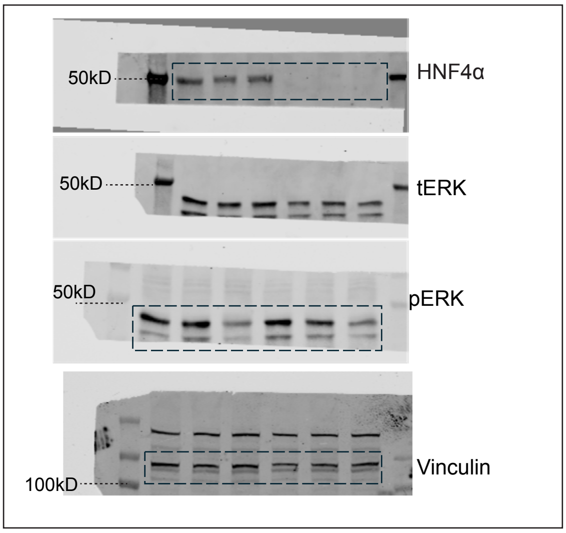

BMS968508(nM) @ 72 hours

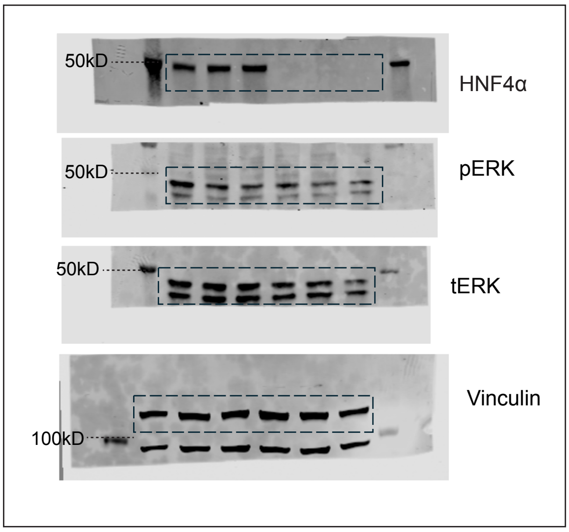

D

Figure 9F

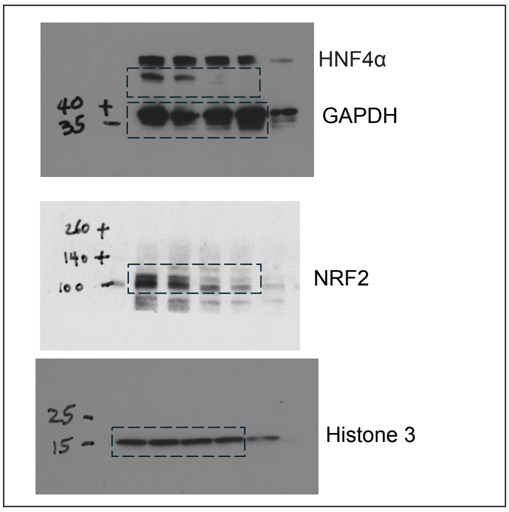

E

Figure 9G

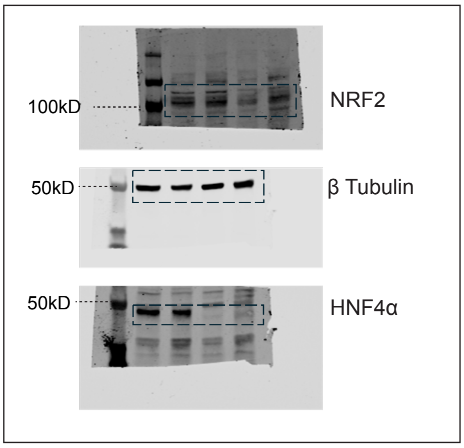

F

Figure 9I

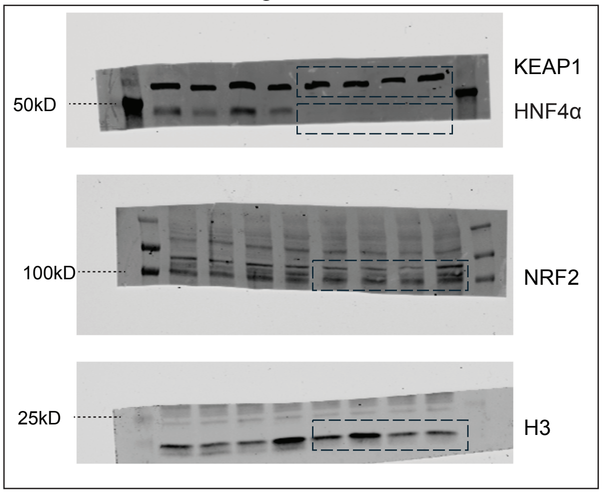

G

Supplemental 12 A

BMS968508(nM) @ 24 hours

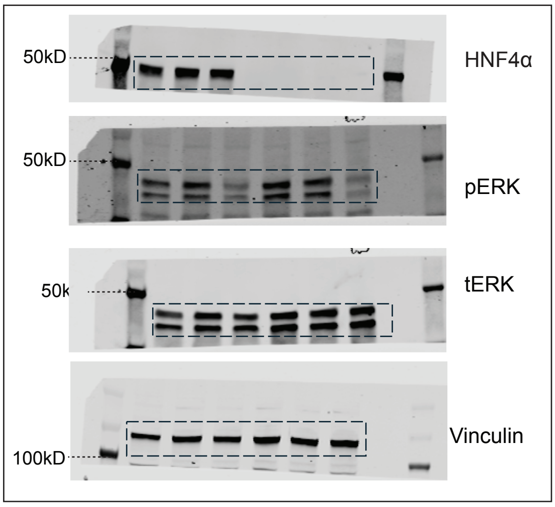

H

Supplemental 12 C

RMC9805(nM) @ 2 hours

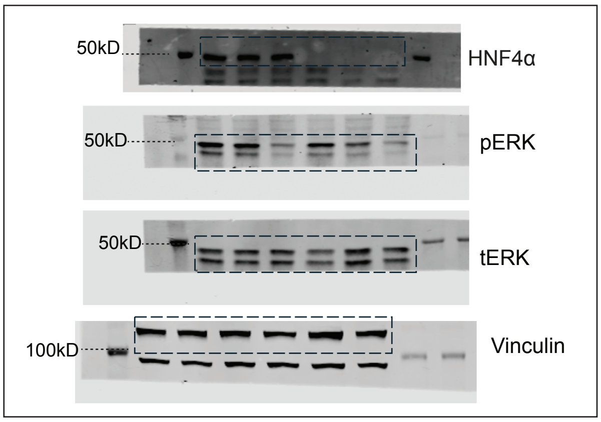

RMC9805(nM) @ 72 hours

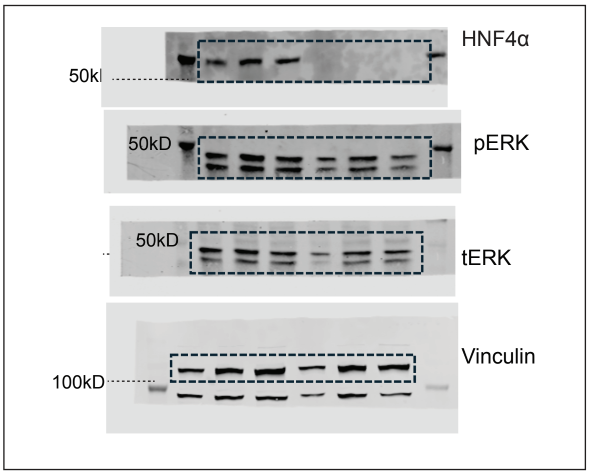

I

Supplemental 15A

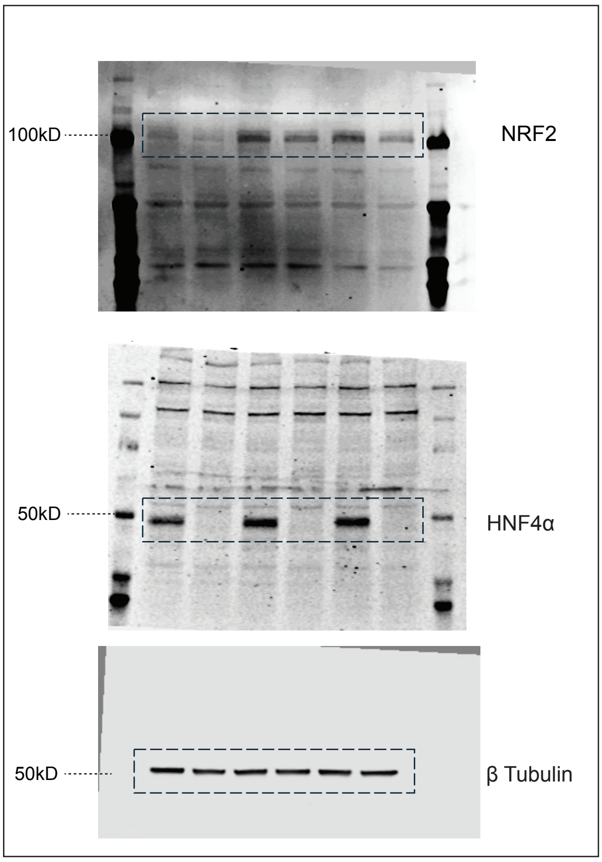

J

Supplemental 15B

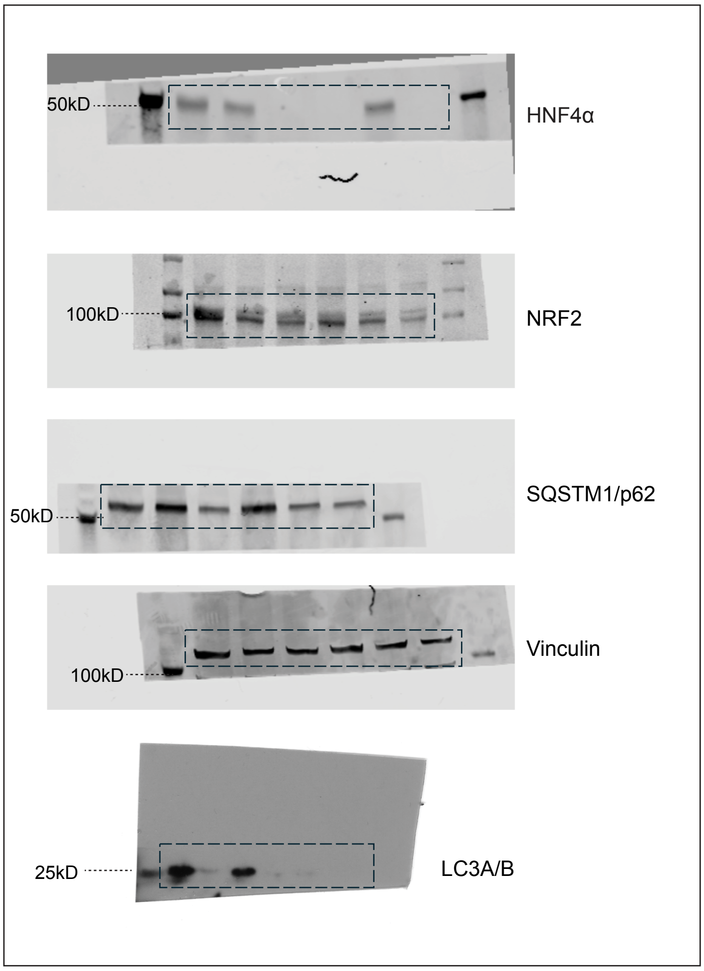

K

Supplemental 15D

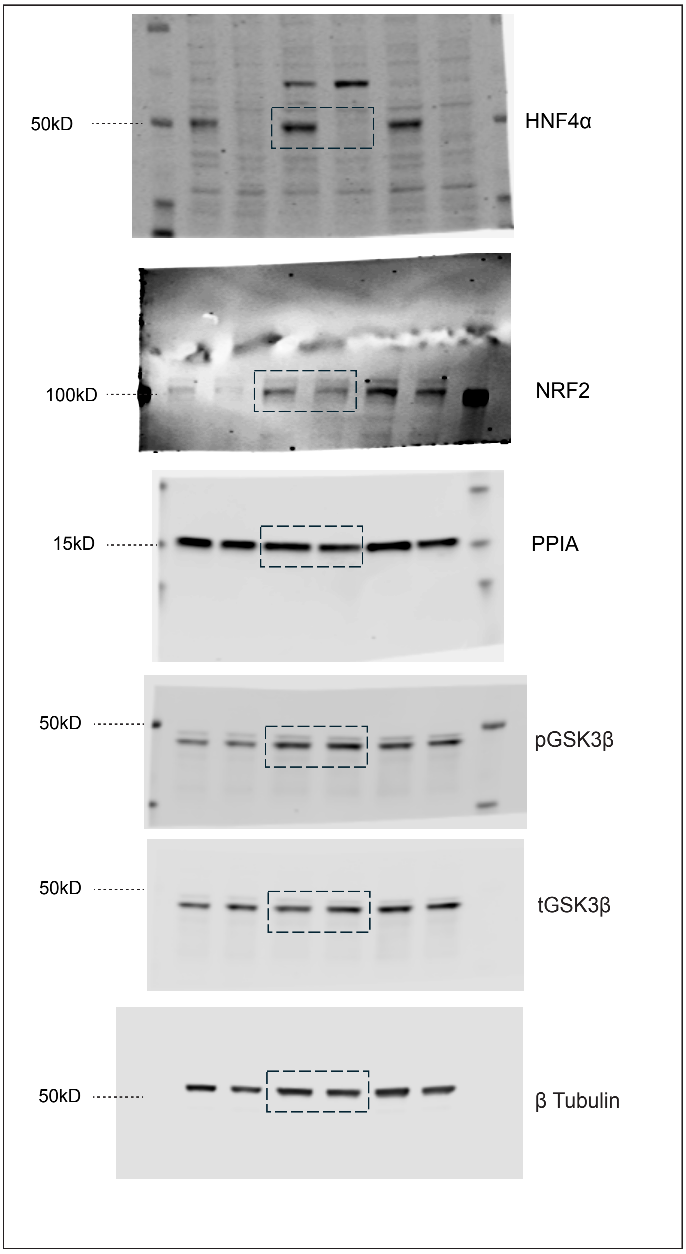

L

Supplemental 15I

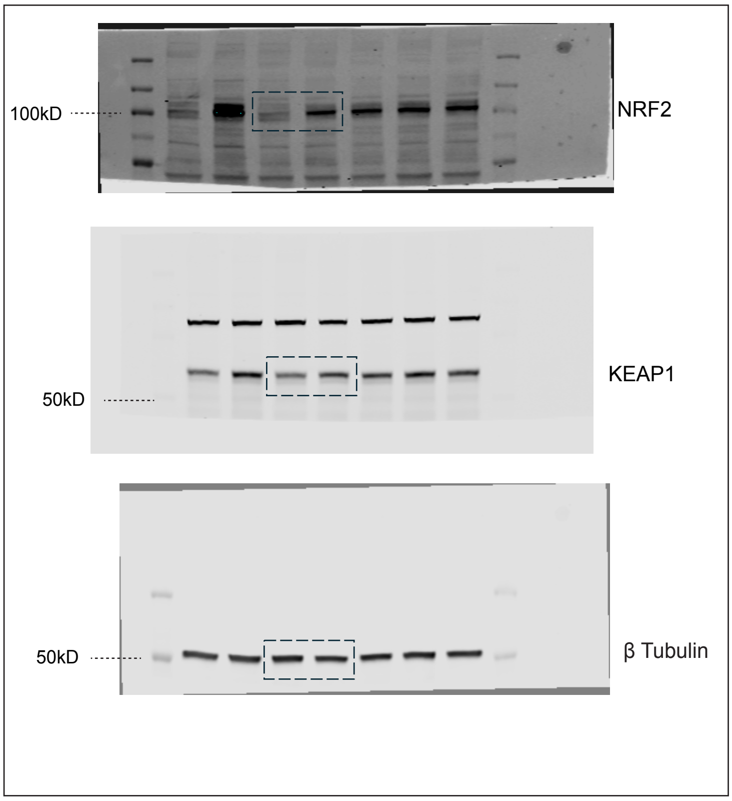

Supplement: Unedited blot and gel images [file jci-136-198282-s177.pdf]
